# Resistance to *Bacillus thuringiensis* Cry1Ac toxin requires mutations in two *Plutella xylostella* ATP-binding cassette transporter paralogs

**DOI:** 10.1371/journal.ppat.1008697

**Published:** 2020-08-10

**Authors:** Zhaoxia Liu, Shu Fu, Xiaoli Ma, Simon W. Baxter, Liette Vasseur, Lei Xiong, Yuping Huang, Guang Yang, Shijun You, Minsheng You

**Affiliations:** 1 State Key Laboratory of Ecological Pest Control for Fujian-Taiwan Crops and College of Life Science, Fujian Agriculture and Forestry University, Fuzhou, China; 2 Institute of Applied Ecology, Fujian Agriculture and Forestry University, Fuzhou, China; 3 Joint International Research Laboratory of Ecological Pest Control, Ministry of Education, Fuzhou, China; 4 Xinjiang Key Laboratory of Biological Resources and Genetic Engineering, College of Life Science and Technology, Xinjiang University, Urumqi, China; 5 School of BioSciences, The University of Melbourne, Melbourne, Victoria, Australia; 6 Department of Biological Sciences, Brock University, St. Catharines, Ontario, Canada; University of Cambridge, UNITED KINGDOM

## Abstract

The diamondback moth, *Plutella xylostella*, is a cosmopolitan pest and the first species to develop field resistance to toxins from the gram-positive bacterium *Bacillus thuringiensis* (Bt). Although previous work has suggested that mutations of ATP-binding cassette transporter subfamily C2 (ABCC2) or C3 (ABCC3) genes can confer Cry1Ac resistance, here we reveal that *P*. *xylostella* requires combined mutations in both *PxABCC2* and *PxABCC3* to achieve high-level Cry1Ac resistance, rather than simply a mutation of either gene. We identified natural mutations of *PxABCC2* and *PxABCC3* that concurrently occurred in a Cry1Ac-resistant strain (Cry1S1000) of *P*. *xylostella*, with a mutation (*R*_*A2*_) causing the mis-splicing of *PxABCC2* and another mutation (*R*_*A3*_) leading to the premature termination of PxABCC3. Genetic linkage analysis showed that *R*_*A2*_ and *R*_*A3*_ were tightly linked to Cry1Ac resistance. Introgression of *R*_*A2*_ and *R*_*A3*_ enabled a susceptible strain (G88) of *P*. *xylostella* to obtain high resistance to Cry1Ac, confirming that these genes confer resistance. To further support the role of *PxABCC2* and *PxABCC3* in Cry1Ac resistance, frameshift mutations were introduced into *PxABCC2* and *PxABCC3* singly and in combination in the G88 strain with CRISPR/Cas9 mediated mutagenesis. Bioassays of CRISPR-based mutant strains, plus genetic complementation tests, demonstrated that the deletion of *PxABCC2* or *PxABCC3* alone provided < 4-fold tolerance to Cry1Ac, while disruption of both genes together conferred >8,000-fold resistance to Cry1Ac, suggesting the redundant/complementary roles of PxABCC2 and PxABCC3. This work advances our understanding of Bt resistance in *P*. *xylostella* by demonstrating mutations within both *PxABCC2* and *PxABCC3* genes are required for high-level Cry1Ac resistance.

## Author summary

*Bacillus thuringiensis* (Bt) foliar sprays and transgenic crops expressing Bt toxins are used extensively to control insect pests, but the evolution of resistance limits their efficacy. Multiple studies have reported that ATP-binding cassette (ABC) transporters are important Bt receptors, and mutations in either *ABCC2* or *ABCC3* can lead to Cry1Ac-toxin resistance, although this process is not fully understood. In this study, we applied both forward and reverse genetic analyses to demonstrate that high-level Bt-Cry1Ac resistance in *Plutella xylostella* requires concurrent mutations in both *PxABCC2* and *PxABCC3*. We identified inactivating mutations in these two genes from a Cry1Ac-resistant strain (Cry1S1000) of *P*. *xylostella* and conducted genetic linkage analysis, which supported the role that *PxABCC2* and *PxABCC3* were the causal genes of Cry1Ac resistance. We then knocked out *PxABCC2* and *PxABCC3* in a *P*. *xylostella* susceptible reference strain (G88) to confirm that high-level Cry1Ac resistance requires mutation of *PxABCC2* and *PxABCC3*, rather than a mutation of either one gene. This finding expands our understanding of complex Bt resistance processes and may be relevant to Bt-Cry1Ac resistance in other lepidopteran insects.

## Introduction

*Bacillus thuringiensis* (Bt) is a ubiquitous gram-positive bacterium that can produce insecticidal protein toxins used for the control of many lepidopteran, coleopteran and dipteran pests [1,2]. Bt toxins often show high insect specificity and are safe for non-target organisms [1], however, like many synthetic agrochemicals, evolution of resistance among pests is threatening their efficacy [3]. By 2019, practical field-evolved resistance to Bt has been documented in at least 22 cases and nine insect species [3–7]. Complete understanding of the mechanisms causing Bt resistance remains limited, although mutations or expression changes in toxin-binding proteins, such as cadherin-like protein, alkaline phosphatase (ALP), aminopeptidase N (APN), and ABC transporters, appear to play important roles in mediating the resistance level [8,9].

ATP-binding cassette transporters, or ABC transporters, consist of two cytosolic nucleotide-binding domains (NBDs) and two integral transmembrane domains (TMDs), each with six membrane-spanning helices [10]. Mutations in ABC transporter subfamily C2 (ABCC2) are associated with Bt (Cry1A toxins) resistance in a number of lepidopteran species [11–17]. The receptor functionality of ABCC2 for Cry1A toxins has been well characterized using heterologous expression in cultured cell lines, manipulating mRNA abundance with RNA interference and with transgenic *Drosophila* [14,18–22]. ABC transporter subfamily C3 (ABCC3), a paralog of ABCC2, has also been reported to mediate Cry1A toxicity [14,22–24]. Recently, Wang et al. [25] reported mutations in ABCC2 plus ABCC3 were required to achieve high-level Cry1Ac resistance in *Helicoverpa armigera*, suggesting functional redundancy as toxin receptors.

The diamondback moth, *Plutella xylostella*, is a notorious pest of cruciferous crops worldwide. Field resistance to Bt spray formulas first arose in this species within a population from Hawaii, USA [5,26]. Baxter et al. [17] have identified a 30-bp deletion in ABCC2 within a Cry1Ac-resistant strain (NO-QAGE) derived from this Hawaiian population. Reduced expressions in ABCC2 and ABCC3 have also been observed in resistant *P*. *xylostella* strains from other countries, including the DBM1Ac-R strain collected from Loxahatchee (Florida, USA) [22]. A recent gene-editing-based investigation provides further evidence of the roles of ABCC2 and ABCC3 in Bt resistance: knockout of either of the two *P*. *xylostella* genes, *PxABCC2* and *PxABCC3*, confers high levels of resistance to Bt Cry1Ac (724 and 413 folds compared to the reference strain DBM1Ac-S) [16]. However, these resistance levels to Cry1Ac were more than 4,000-fold lower than a selected resistant strain [16].

In this study, we tested the hypothesis that mutations in both *P*. *xylostella ABCC2* and *ABCC3* are required to achieve a high-level Cry1Ac resistance in natural populations, while mutations in either gene confer low-level resistance. To do so, the sequences of *PxABCC2* and *PxABCC3* were compared between a Cry1Ac-resistance strain (Cry1S1000) and a susceptible strain (G88) of *P*. *xylostella* to identify the potential mutations. We then tested the genetic linkage of these two genes with Cry1Ac resistance and analyzed their contribution to Bt resistance by introgressing *PxABCC2* and *PxABCC3* from Cry1S1000 into G88. Finally, we introduced mutations into these ABCC genes separately and together in a reference strain with the CRISPR/Cas9 system, and then assessed their capacity to confer resistance to Cry1Ac using bioassays and genetic complementation tests.

## Results

### Resistance of the Cry1S1000 strain to Cry1Ac protoxin

Bioassays performed with artificial diet overlay assays indicated that the concentration of Cry1Ac protoxin capable of killing 50% of resistant Cry1S1000 larvae (LC_50_) was higher than 1,000 μg/ml (S1 Table). The Cry1S1000 strain was > 8,000 fold more resistant to Cry1Ac than the susceptible reference, G88 (S1 Table). In subsequent generations without Bt selection, Cry1S1000 maintained stable resistance to Cry1Ac (LC_50_ > 1,000 μg/ml) and showed 100% survival at a diagnostic concentration of 0.5 μg/ml of Cry1Ac protoxin (S2 Table).

### ABCC2 and ABCC3 sequence comparison between *P*. *xylostella* strains

**Fig 1:**
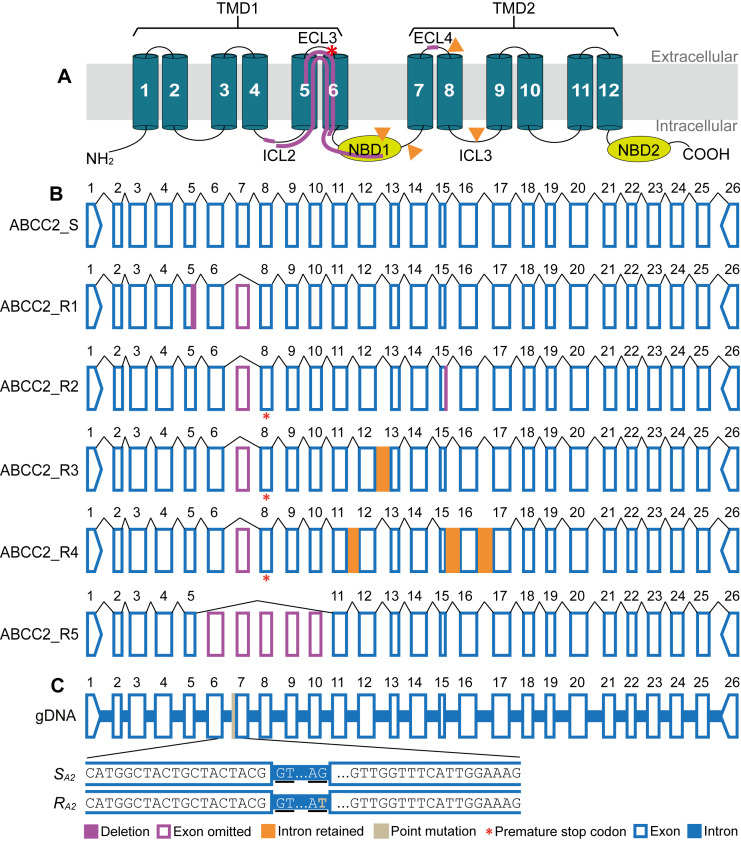
ABCC2 in the Cry1Ac-susceptible and -resistant strains of *P*. *xylostella*. (**A**) Schematic structure of PxABCC2 protein showing the position of deletions (path lines in purple) and insertions (orange triangles) in the Cry1S1000 strain. The predicted protein contains two transmembrane domains (TMD1 and TMD2) each with 6 transmembrane helices (dark green cylinders) and two nucleotide-binding domains (NBD1 and NBD2, yellow ovals). ECL: extracellular loop; ICL: intracellular loop. Transmembrane helices were predicted by Phobius (http://phobius.sbc.su.se/). (**B**) Schematic representation of *PxABCC2* exonic sequence of the susceptible G88 strain (ABCC2_S) and five transcript variants (ABCC2_R1-5) of the resistant Cry1S1000 strain. Exons are numbered (1–26). (**C**) Genomic structure of *PxABCC2* illustrating sequences of *S*_*A2*_ and *R*_*A2*_ allele. Underlined letters GT/AG indicate the splice sites.

Full-length *PxABCC2* transcripts were sequenced using midgut cDNA template generated from Bt-susceptible G88 larvae. The coding sequence was 4,044 bp and encoded a predicted 1,347 aa protein (S1 and S2 Figs) that displayed a typical structure among known lepidopteran ABCCs [11], including two transmembrane domains (TMD1 and TMD2), each with six transmembrane helices, and two nucleotide-binding domains (NBD1 and NBD2) (Fig 1A) containing conserved Walker A, B and C motifs (S2 Fig).

Five independent *PxABCC2* transcript variants from the resistant Cry1S1000 strain were found to contain a range of insertions or deletions, however, all lacked exon 7 (ABCC2_R1-5 in Fig 1B, Table 1, S1 Fig). Two isoforms (ABCC2_R1 and ABCC2_R5) had in-frame deletions in exon 5 (Fig 1B), disrupting the portion of PxABCC2 protein from intracellular loop 2 to NBD1 (Fig 1A, Table 1, S2 Fig). The remaining three isoforms (ABCC2_R2-4) had frameshift mutations that introduced premature stop codons at extracellular loop 3 (Fig 1A, Table 1, S2 Fig).

**Table 1:** *PxABCC2* isoforms of *P*. *xylostella* strain Cry1S1000.

Isoform	Predicted protein length^*a*^	Premature stop codon	Exon(s) or intron(s) affected	Size(s) of deletion (bp)^*b*^	Size(s) of insertion (bp)^*c*^
ABCC2_R1	1,302	No	Exon 5 (splicing error in intron 5), exon 7 omitted	8, 127	-
ABCC2_R2	362	Yes	Exon 7 omitted, exon 15 (splicing error in intron 14)	127, 29	-
ABCC2_R3	362	Yes	Exon 7 omitted, intron 12 retained	127	75
ABCC2_R4	362	Yes	Exon 7 omitted, introns 11, 15 and 16 retained	127	313, 256, 345
ABCC2_R5	1,079	No	Exons 6–10 omitted	804	-

^*a*^ Wild type length is 1,347 amino acids.

^*b*^ Deletions indicate the partial or complete exon(s) missing caused by alternative splicing or mis-splicing.

^*c*^ Insertions indicate the complete intron(s) retained caused by alternative splicing.

All Cry1S1000 *PxABCC2* isoforms lacked exon 7, however, genomic DNA amplicon sequencing revealed the coding sequence was present in the genome and not simply a chromosomal deletion. A point mutation (G>T) in the 3′ splice junction of intron 6 was identified, which would disrupt pre-mRNA processing and prevent the incorporation of exon 7 into the mature mRNA transcript. Hereafter, we refer to this genomic DNA mutation as “*R*_*A2*_” (Fig 1C and S3 Fig).

Alignments between gDNA and cDNA sequences also found an 8-bp deletion in ABCC2_R1 was caused by an alternative 5′ splice site in intron 5, and a 29-bp deletion in ABCC2_R2 was caused by an alternative 3′ splice site in intron 14 (Table 1, S3 and S4 Figs). The insertions were found in two other isoforms, which were caused by the retention of introns in the mature mRNA (intron 12 in ABCC2_R3 and introns 11, 15 and 16 in ABCC2_R4, S4–S6 Figs). Finally, an 804-bp deletion in ABCC2_R5 was caused through skipping exons 6–10 (Fig 1B).

Intron 6 varied in size between G88 (581 bp, *S*_*A2*_) and Cry1S1000 (429 bp, *R*_*A2*_), which enabled development of an allele-specific PCR assay (AS-PCR) capable of quickly differentiating between susceptible and resistant alleles (S7 Fig).

**Fig 2:**
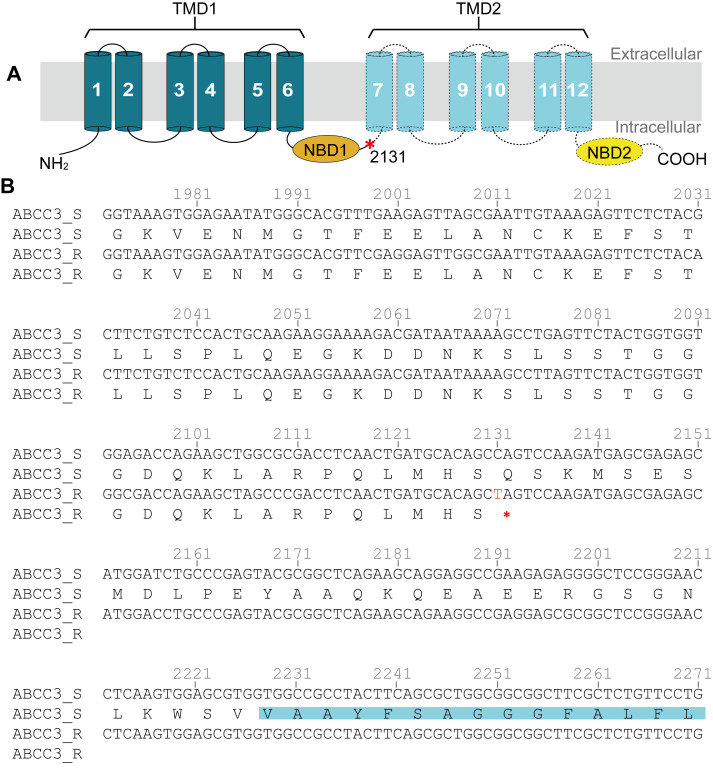
ABCC3 in the Cry1Ac-susceptible and -resistant strains of *P*. *xylostella*. (**A**) Schematic illustration of PxABCC3 protein showing the position of the premature stop codon (asterisk in red) from the resistant Cry1S1000 strain. The PxABCC3 protein is predicted to contain two transmembrane domains with 6 transmembrane helices (dark green cylinders) for TMD1 and 6 transmembrane helices (light blue cylinders) for TMD2, and two nucleotide-binding domains (NBD1 and NBD2). Transmembrane helices, except for TM1 and TM2, were predicted by Phobius. The dotted lines, TMD2 in light blue and NBD2 in yellow represent the predicted region missing in the PxABCC3 from Cry1S1000. (**B**) The partial cDNA sequences and the deduced protein sequences from the G88 and Cry1S1000 strains show the point mutation of C2131T (the letter in red) in the Cry1S1000 strain that leads to the premature termination of PxABCC3. Amino acid residues highlighted in light blue are the portion of transmembrane helix 7.

*PxABCC3* cDNA from the G88 (ABCC3_S) contained an open reading frame (ORF) of 4,047 bp and encoded a predicted protein of 1,348 aa (S8 and S9 Figs). Similar to PxABCC2, the PxABCC3 protein also consisted of two transmembrane domains (TMD1 and TMD2) and two nucleotide-binding domains (NBD1 and NBD2) (Fig 2A and S9 Fig). A point mutation (C>T) identified at nucleotide 2131 in Cry1S1000 *PxABCC3* cDNA (ABCC3_R) (Fig 2B and S8 Fig) introduced a premature stop codon just prior to transmembrane helix 7, truncating the protein (Fig 2A and S9 Fig). This allele was referred as “*R*_*A3*_”, and the susceptible *PxABCC3* allele “*S*_*A3*_”. Size variation was again used to develop an AS-PCR in *PxABCC3* using primers spanning intron 13 (480 bp in G88 and 546 bp in Cry1S1000) (S10 Fig).

### Genetic linkage between *R*_*A2*_/*R*_*A3*_ and Cry1Ac resistance

**Fig 3:**
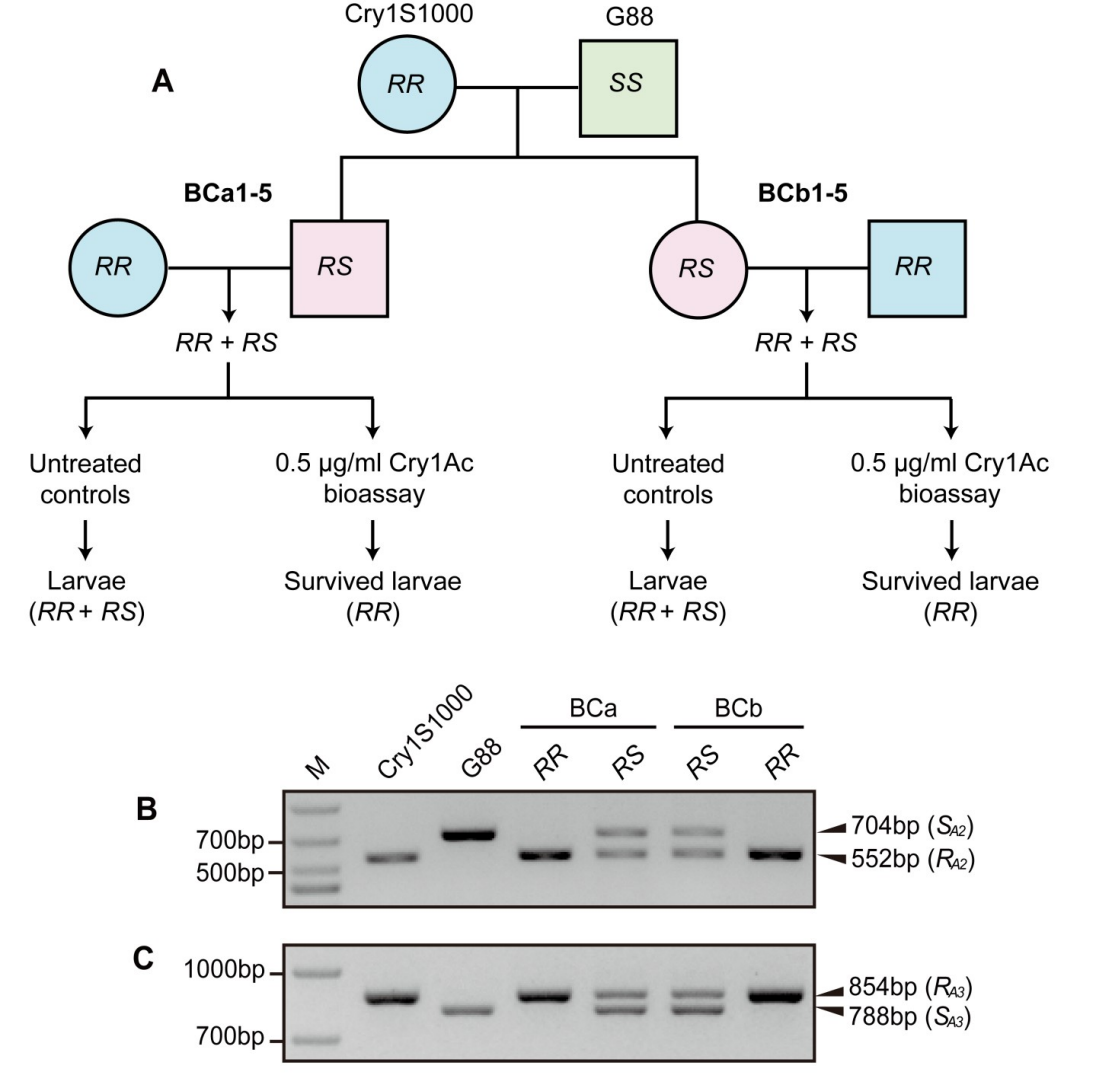
Genetic linkage analysis of *R*_*A2*_ and *R*_*A3*_ with Cry1Ac resistance. (**A**) Diagram showing the strategy for backcrossing and bioassays. A resistant female (Cry1S1000) was crossed with a susceptible male (G88) to produce F_1_ progeny. Two types of backcrosses were made between F_1_ and Cry1S1000: BCa1-5 families were produced by single-pair crosses between an F_1_ male and Cry1S1000 female, and BCb1-5 families were produced by single-pair cross between an F_1_ female and Cry1S1000 male. Only the resistant backcross progeny (*RR*) survived the discriminating 0.5 μg/ml Cry1Ac bioassay. (**B**,**C**) Genotyping of *PxABCC2* (**B**) and *PxABCC3* (**C**) in Cry1S1000 (*R*_*A2*_*R*_*A2*_ or *R*_*A3*_*R*_*A3*_) or G88 (*S*_*A2*_*S*_*A2*_ or *S*_*A3*_*S*_*A3*_) individuals, and their F_1_ progeny (*R*_*A2*_*S*_*A2*_ or *R*_*A3*_*S*_*A3*_) using AS-PCR.

We tested for genetic linkage between *R*_*A2*_/*R*_*A3*_ alleles and Cry1Ac resistance using 10 backcross families, which were obtained from single-pair crosses between Cry1S1000 (*RR*) and an F_1_ (*RS*, from Cry1S1000♀ × G88♂) (Fig 3A). Bioassays were performed on 50 larvae from each of 10 backcross families (n = 500) and 224 survived after exposed to 0.5 μg/ml Cry1Ac protoxin. A total of 447 untreated control larvae were also collected (S3 Table).

We first genotyped *PxABCC2* and *PxABCC3* in all cross parents using AS-PCRs (Fig 3B and 3C) and confirmed Cry1S1000 individuals were homozygous for the *R*_*A2*_ and *R*_*A3*_ alleles and only detected *S*_*A2*_ and *S*_*A3*_ alleles among G88 individuals. All F_1_ progeny from Cry1S1000 × G88 mating were heterozygous for the *R*_*A2*_/*S*_*A2*_ and *R*_*A3*_/*S*_*A3*_ alleles (Fig 3B and 3C). Among untreated control backcross progeny, we expected half the individuals to be heterozygous (*R*_*A2*_/*S*_*A2*_, *R*_*A3*_/*S*_*A3*_) and half to be homozygous for ABCC mutations (*R*_*A2*_/*R*_*A2*_, *R*_*A3*_/*R*_*A3*_) and observed numbers did not significantly differ (216 heterozygous, 231 homozygous, Fisher’s exact test *P* > 0.41 for each control family) (S3 Table). Among insecticide treated larvae, no heterozygous *R*_*A2*_/*S*_*A2*_, *R*_*A3*_/*S*_*A3*_ individuals were detected, indicating the Cry1Ac discriminating dose was sufficient to kill carriers of ABCC2 and ABCC3 G88 alleles (Fisher’s exact test for each treated family, *P* < 0.0005) (S3 Table). Therefore, Cry1Ac resistance was genetically linked to *R*_*A2*_ and *R*_*A3*_ alleles in the Cry1S1000 strain.

### Introgression of *R*_*A2*_/*R*_*A3*_ alleles into the genetic background of G88

To eliminate potential differences in fitness between strains, and assess whether loci that were not linked to ABCC mutations contributed to Cry1Ac resistance, *R*_*A2*_ and *R*_*A3*_ alleles were introgressed into G88. Cry1S1000 males were crossed to G88 females, then F_1_ progeny backcrossed to G88 for a further six generations. Selection of the *R*_*A2*_ allele was performed in each generation backcrossing using AS-PCR. Random mating between progeny of backcross six with heterozygous genotypes (*R*_*A2*_*S*_*A2*_) were used to establish a near-isogenic line to the G88 strain (G88-R_A2_) that was homozygous for both the *R*_*A2*_ and *R*_*A3*_ alleles (S4 Table). Diet bioassays confirmed the G88-R_A2_ strain was equally resistant to Cry1S1000 (>1,000 μg/ml Cry1Ac protoxin, S1 Table).

Reciprocal crosses between resistant strains (Cry1S1000 and G88-R_A2_) and G88 were performed and susceptibility of F_1_ progeny to the diagnostic concentration (0.5 μg/ml) of Cry1Ac protoxin was assessed. The mass crosses between each of the two resistant strains with G88 resulted in no survival of F_1_ progeny (i.e., Cry1S1000♀ × G88♂, G88♀ × Cry1S1000♂, G88-R_A2_♀ × G88♂, and G88♀ × G88-R_A2_♂) (S5 Table). This indicated that the Cry1S1000 and G88-R_A2_ strains exhibited autosomal and completely recessive (*h* = 0) resistance to Cry1Ac at the diagnostic concentration. Third-instar larval progeny from ten single-pair crosses between Cry1S1000 and G88-R_A2_ were assayed and showed 100% survival after a 5-day bioassay (n = 385), confirming the strains shared a common, recessive resistance locus (Index of commonality = 1) (S5 Table).

### Mutagenesis of *PxABCC2* and *PxABCC3* mediated by CRISPR/Cas9

To knock out *PxABCC2* and *PxABCC3*, we injected mixtures of sgRNA and Cas9 protein (or mRNA) into fresh eggs from the wild type G88 strain. Successful site-specific mutations within *PxABCC2* and *PxABCC3* were identified within mosaic G_0_ moths (S6 Table).

**Fig 4:**
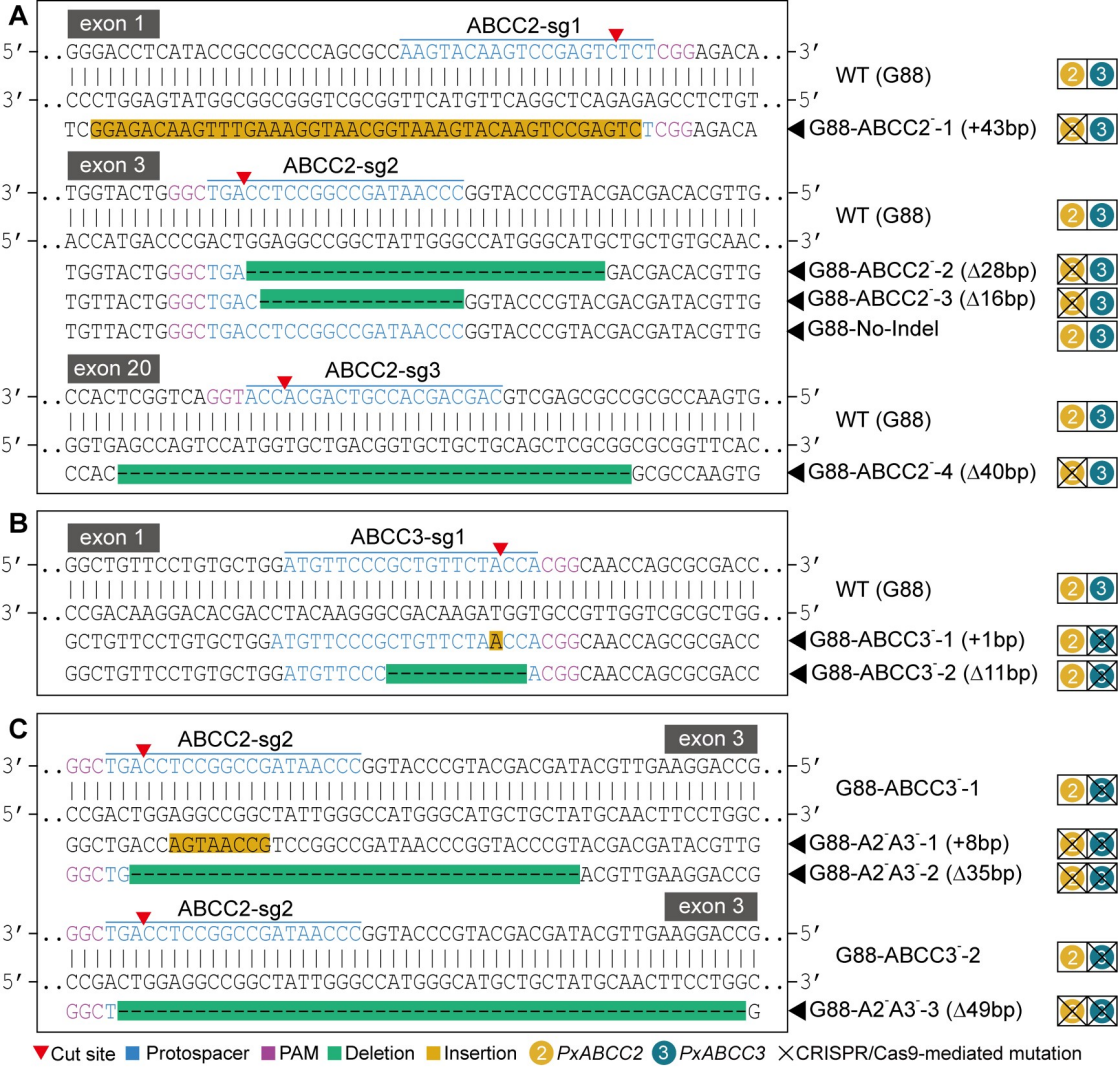
Mutagenesis of *PxABCC2* and *PxABCC3* induced by CRISPR/Cas9. (**A**) Representative sequences from the wild type (G88) and four homozygous *PxABCC2* mutant strains, showing the deletions and insertions (indels) in exon 1, 3 and 20 of *PxABCC2*. (**B**) Partial sequences from the G88 and two homozygous *PxABCC3* mutants showing the indels at the target sequence (ABCC3-sg1) in exon 1 of *PxABCC3*. (**C**) Representative sequences from two *PxABCC3* mutants and three *PxABCC2*/*PxABCC3* double mutant strains showing the indels at the target sequence (ABCC2-sg2) in exon 3 of *PxABCC2*.

Four homozygous mutant *PxABCC2* strains were generated: G88-ABCC2^-^-1 had a 43-bp insertion in exon 1, G88-ABCC2^-^-2 a 28-bp deletion in exon 3, G88-ABCC2^-^-3 a 16-bp deletion in exon 3, and G88-ABCC2^-^-4 a 40-bp deletion in exon 20 (Fig 4A and S11 Fig). Embryos microinjected with *PxABCC2-exon 3* sgRNA that failed to generate targeted indel mutation were used to generate the line G88-No-Indel and acted as a negative control to analyze potential off-target effect (Fig 4A and S11B Fig). The four ABCC2 CRISPR/Cas9 strains were all predicted to carry *PxABCC2* frameshift mutations producing truncated proteins (S12A Fig).

Nano-LC-MS/MS sequencing of brush border membrane vesicles (BBMV) proteins ~ 120–200 kDa in the G88 strain midgut identified 1,685 peptides, 34 of which were specific to PxABCC2 (S12B–S12D Fig). Although 1,179 peptides were detected from the G88-ABCC2^-^-1 mutant, none of them were PxABCC2 sequences (S7 Table), supporting total disruption of PxABCC2 protein in this *PxABCC2*-knockout strain.

For *PxABCC3*, two homozygous mutant strains with either a 1-bp insertion or a 11-bp deletion in exon 1 were generated (G88-ABCC3^-^-1 and G88-ABCC3^-^-2) (Fig 4B and S13 Fig). Nano-LC-MS/MS analysis for ~ 90–200 kDa midgut BBMV proteins detected 2,239 tryptic peptides from the G88 strain, 33 of which were specific to the PxABCC3 protein (S14 Fig). A total of 2,553 and 1,968 peptides were detected from G88-ABCC3^-^-1 (S8 Table) and G88-ABCC3^-^-2 (S9 Table), respectively, and none were PxABCC3 peptides. This supported a complete loss of PxABCC3 protein in both *PxABCC3*-knockout strains.

To produce *PxABCC2*/*PxABCC3* double mutant strains, frameshift mutations in *PxABCC2* exon 3 were then introduced through microinjecting embryos from the G88-ABCC3^-^-1 and G88-ABCC3^-^-2 CRISPR/Cas9 strains. Two double mutant strains were established from G88-ABCC3^-^-1 strain, including G88-A2^-^A3^-^-1 (8-bp *PxABCC2* exon 3 insertion) and G88-A2^-^A3^-^-2 (35-bp exon 3 deletion) (Fig 4C), and one double mutant strain was established from G88-ABCC3^-^-2 that we name G88-A2^-^A3^-^-3 (49-bp *PxABCC2* exon 3 deletion) (Fig 4C). Nano-LC-MS/MS analysis of ~ 120–250 kDa midgut BBMV proteins from G88 larvae yielded 2,048 peptides, 27 of which were PxABCC2 and 40 PxABCC3 (S15 Fig). However, we found no evidence of PxABCC2 and PxABCC3 proteins in the double mutant G88-A2^-^A3^-^-1 when the 3,159 peptides sequenced were examined (S10 Table), indicating complete knockout of both proteins in this strain.

### Effect of *PxABCC2* and *PxABCC3* mutations on *P*. *xylostella* susceptibility to Cry1Ac protoxin

**Fig 5:**
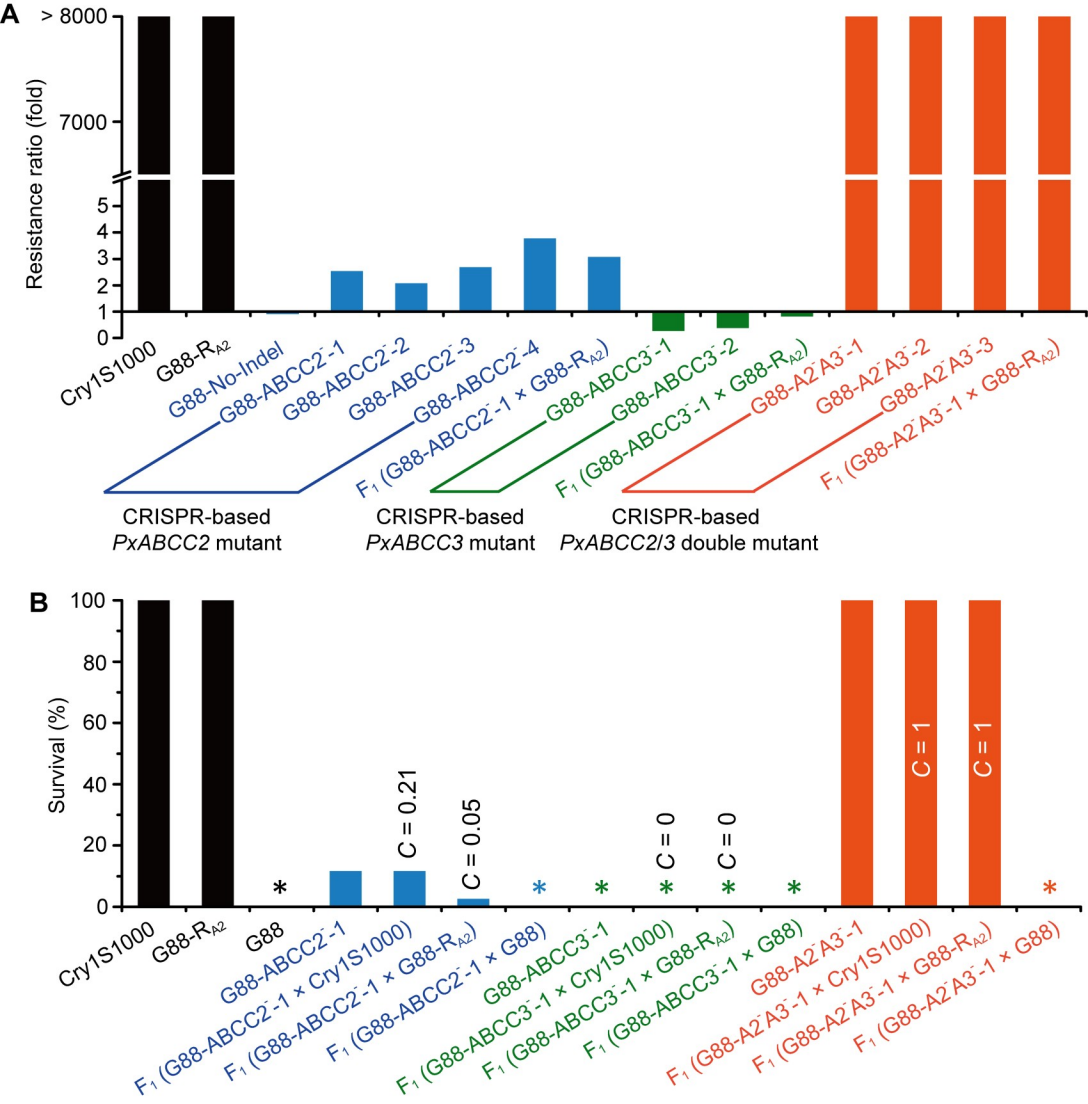
Response of different *P*. *xylostella* strains to Cry1Ac protoxin. (**A**) Resistance level (fold) of two resistant strains (Cry1S1000 and G88-R_A2_), a negative control (G88-No-Indel), four CRISPR-based *PxABCC2* mutants, two CRISPR-based *PxABCC3* mutants, three CRISPR-based *PxABCC2*/*PxABCC3* double mutants and the F_1_ progeny from mass crosses between each of three CRISPR-based mutants and G88-R_A2_ compared to the G88 strain. The resistance ratio (fold) for G88 is 1. (**B**) Survivals at the diagnostic concentration (0.5 μg/ml) of Cry1Ac protoxin of *P*. *xylostella* larvae from two resistant strains (Cry1S1000 and G88-R_A2_), a susceptible strain (G88), a CRISPR-based *PxABCC2* mutant (G88-ABCC2^-^-1), a CRISPR-based *PxABCC3* mutant (G88-ABCC3^-^-1), a CRISPR-based *PxABCC2*/*PxABCC3* double mutant (G88-A2^-^A3^-^-1), and the F_1_ progeny from single-pair crosses between each of three CRISPR-based mutants and each of two resistant strains or G88. Asterisks indicate 0% survival. Index of commonality (*C*) indicates the proportion of the two strains sharing the common resistance locus or loci.

The bioassays indicated that knockout of *PxABCC2* in four mutant strains (i.e., G88-ABCC2^-^-1, G88-ABCC2^-^-2, G88-ABCC2^-^-3 and G88-ABCC2^-^-4) resulted in a decline in susceptibility to Cry1Ac protoxin between 2.45–3.78 folds relative to the G88 reference strain. The negative control of G88-No-Indel strain showed similar Cry1Ac susceptibility as the G88 strain. The two *PxABCC3* mutant strains, G88-ABCC3^-^-1 and G88-ABCC3^-^-2, surprisingly showed a slight increase in susceptibility in comparison to G88. The three *PxABCC2*/*PxABCC3* double mutant strains showed > 8,000-fold resistance to Cry1Ac compared with the G88 strain (Fig 5A, S1 Table).

To further determine the contribution of recessive mutations in two *ABCC* loci to Cry1Ac resistance, we performed complementation tests for *PxABCC2* and *PxABCC3* between resistant strains and CRISPR-based mutants. The average survival rates when exposed to 0.5 μg/ml Cry1Ac protoxin were 11.7% (7/60) for G88-ABCC2^-^-1, 11.7% (0.0% - 20.0%) for the F_1_ progeny from single-pair crosses between G88-ABCC2^-^-1 and Cry1S1000, and 2.6% (0.0% - 21.7%) for the F_1_ progeny from single-pair crosses between G88-ABCC2^-^-1 and G88-R_A2_ (Fig 5B). The results suggested that two mutations within *PxABCC2* conferred a low level of resistance to Cry1Ac. Similarly, no survival of F_1_ progeny from single-pair crosses between G88-ABCC3^-^-1 and each of two resistant strains was observed, indicating that two mutations at the *PxABCC3* locus failed to confer Cry1Ac resistance. However, the F_1_ progeny produced from crossing the double mutant strain G88-A2^-^A3^-^-1 and Cry1S1000 or G88-R_A2_ were highly resistant to Cry1Ac like their parent strains (Fig 5B). These results showed that two mutations at each of *PxABCC2* and *PxABCC3* loci were required for high levels of resistance.

Consistent with the results from single-pair crosses, the F_1_ progeny from mass crosses between G88-ABCC2^-^-1 and G88-R_A2_, and between G88-ABCC3^-^-1 and G88-R_A2_ retained comparable susceptibility to Cry1Ac as G88 (Fig 5A, S1 Table). In addition, the bioassay test showed that F_1_ progeny from a mass cross between the G88-A2^-^A3^-^-1 CRISPR generated strain and G88-R_A2_ introgression strain exhibited > 8,000-fold resistance to Cry1Ac compared with G88 (Fig 5A, S1 Table).

## Discussion

The complex mode of action of Bt insecticides requires activated toxins to interact with receptors on the brush border membrane of the insect midgut [27]. In this study, we provide rigorous support for the duel involvement of two ABC transporter subfamily genes, *PxABCC2* and *PxABCC3*, in mediating Bt Cry1Ac protoxin susceptibility in *P*. *xylostella*. CRISPR/Cas9-mediated mutagenesis verified that high-level resistance to Cry1Ac only occurred after introducing mutations into both *PxABCC2* and *PxABCC3* alleles, while mutations in one gene alone had little effect. Therefore, PxABCC2 and PxABCC3 appear to function as redundant Cry1Ac receptors for determining susceptibility of Cry1Ac in *P*. *xylostella*. This finding supports recent research by Wang et al. [25] demonstrating that the knockout of *ABCC2* and *ABCC3* are required for Cry1Ac resistance in *H*. *armigera*.

Here we identified five mutated transcripts of *PxABCC2* (ABCC2_R1-5) in the resistant Cry1S1000 strain, and although variation in transcripts sequence was observed, all appeared to be directly caused by the *R*_*A2*_ splice-site mutation of intron 6. One of these transcripts, ABCC2_R1 was previously identified by Guo et al. and reported as isoform VIIa from a resistant strain (DBM1Ac-R) of *P*. *xylostella* [22], and the loss of exon 7 from sequenced transcripts was a common feature of both these studies [22].

The *PxABCC2 R*_*A2*_ splice-site mutation described here in Cry1S1000 is independent from another 30-bp deletion in exon 20 of another Bt resistant *P*. *xylostella* NO-QAGE strain [17]. This suggests that multiple mutations can occur in Bt-selected populations. Expressing PxABCC2 in *Drosophila* midgut confirmed the transmembrane protein can act as a Cry1Ac toxin receptor [21], however, mutations in *PxABCC3* have never been reported in NO-QAGE nor any *P*. *xylostella* Bt resistant strain to the best of our knowledge. A premature stop codon predicted in *PxABCC3* (*R*_*A3*_) of Cry1S1000 here appeared to prevent translation.

Reducing expression levels of either *PxABCC2* or *PxABCC3* mRNA through RNAi knockdown has previously been shown to reduce mortality in wild type *P*. *xylostella* larvae when challenged with discriminating doses of Cry1Ac protoxin [22]. Simultaneous reduction of *PxABCC2*, *PxABCC3* and membrane-bound ALP (*PxmALP*) transcripts using RNAi reduces mortality more than *ABCC2* or *ABCC3* alone [22], providing some support for a combined role of receptors in Cry1Ac mode of action. Reducing or eliminating *PxABCC2* and *PxABCC3* through transcriptional regulators has also been proposed as a mechanism for Cry1A resistance development without requiring genetic mutations within the genes themselves [22].

Although many lepidopteran insects are susceptible to Cry1Ac toxins, it is currently unclear whether mutations in *ABCC2* and *ABCC3* are required for high-level resistance in all species. Some of the first examples with *Heliothis virescens* and *Bombyx mori* [11,12] provide functional support that ABCC2 is required for Cry1A susceptibility. The *B*. *mori* Cry1Ab/c resistant strain, “C2”, was rescued to a susceptible phenotype through piggyback transformation that introduced a wild-type copy of BmABCC2 [11]. Mutations in BmABCC3 have not been reported, however, only rescue of one receptor may be required for function. CRISPR/Cas9-mediated double knockout of *H*. *armigera* ABCC2 and ABCC3 confers a >15,000-fold resistance to Cry1Ac [25]. Knockout of *ABCC2* in *Ostrinia furnacalis* causes fairly high level of resistance to Cry1F, but not Cry1A toxins [28]. In *Spodoptera exigua*, however, ABCC2 mutation appears to be sufficient to confer resistance to Cry1Ac [15].

Expressing ABCC2 in cell lines of both *H*. *virescens* and *B*. *mori* cause cells to swell or lyse and the effect is synergized when co-expressed with another well-characterized Bt toxin receptor, a cadherin-like protein (CaLP) [19,20]. However, the involvement of *P*. *xylostella* CaLP in Cry1Ac toxicity is still controversial [29–31]. Synergism between ABCC2 and CaLP has also been demonstrated *in vivo*. Mutations in either the *Trichoplusia ni* cadherin or ABCC2 only slightly reduce larval susceptibility to a variant of Cry1Ac, while deletion of both proteins confers much higher levels of resistance [32]. Deficiencies of *H*. *virescens* 12-cadherin-domain protein (HevCaLP) and ABCC2 (HevABCC2) offer higher resistance to Cry1Ac than a defect in either one [12].

The results here support redundant roles between PxABCC2 and PxABCC3 in conferring Cry1Ac susceptibility, and differ from two previous studies with diamondback moth [16,22]. In their cases, reduced expression of either *PxABCC2* or *PxABCC3* using RNAi decreased Cry1Ac susceptibility of *P*. *xylostella* larvae [22] and knockout of either *PxABCC2* or *PxABCC3* using CRISPR/Cas9 caused high levels of resistance to Cry1Ac [16]. Although we used artificial-diet bioassay and the previous studies used leaf-dip bioassay, this seems unlikely to be responsible for such differences in these results. Transcripts of ABCC2 and ABCC3 sequenced from our susceptible reference strain (G88) all contained full-length open reading frames, however, Guo et al. [22] reported a low frequency of alternatively spliced isoforms from the control strain in their study. Control strains that express different ABCC2 and ABCC3 alleles, or control strains that produce different mRNA isoforms could potentially explain the discrepancies between our work and the earlier works of Guo et al. [16,22].

Generating *PxABCC2* mutation in the G88 wild-type strain led to a slightly higher resistance to Cry1Ac protoxin, yet *PxABCC3* mutations caused higher susceptibility. Similarly, cultured cells of *B*. *mori* and *S*. *exigua* exhibit higher Cry1Aa susceptibility when expressing ABCC2 than ABCC3 [24,33]. Contribution of ABCC2 in determining toxicity of Cry1Ac may therefore be more significant than that of ABCC3. In *B*. *mori*, the extracellular loop 1 (ECL1) in BmABCC2/3 transporters determines receptor specificity for Cry1A toxins [33]. The loop 1 Q125 residue of SfABCC2 is also the key factor for the high toxicity of Cry1Ac to Hi5 cells expressing this protein [34]. Structure-based evidence therefore is necessary to further verify the roles of ECL1 in determining the specificity of PxABCC2 and PxABCC3 for Cry1Ac toxicity, and to better address their potential synergism in terms of functional complementation or redundancy. In addition, PxABCC2 has been associated with Cry1Ac oligomerization and oligomer membrane insertion [35]. Our knockout mutants of *PxABCC2*, *PxABCC3* or both genes provide new opportunities to test this hypothesis and investigate whether PxABCC3 plays a similar role in Cry1Ac oligomerization.

Our work indicates that *P*. *xylostella* has evolved resistance to Cry1Ac protoxin through mutations in both *PxABCC2* and *PxABCC3*. DNA-based screening may be a useful tool for monitoring the frequency of *P*. *xylostella* field resistance to Cry1A toxins, caused by mutations in ABCC genes. However, additional allelic variants causing mutations that affect toxin binding are likely to exist in field populations, and this needs to be considered. The present finding provides strong evidence for understanding the roles of ABC transporters in Bt mode of action and the genetic mechanisms that lead to Bt resistance in *P*. *xylostella*.

## Materials and methods

### Insect sample

The *Plutella xylostella* strains were established by Dr. Anthony M. Shelton (Cornell University, USA) and provided to the Institute of Applied Ecology, Fujian Agriculture and Forestry University, in 2016. The insecticide susceptible reference strain, Geneva 88 (G88), was collected from the New York State Agricultural Experiment Station in 1988 and maintained on artificial diet without exposure to insecticides [36]. A population collected from Loxahatchee, Florida, in 1992 was resistant to *B*. *thuringiensis* subsp. *kurstaki* spray formula [37]. It was subsequently reselected with transgenic broccoli that only expressed the Cry1Ac toxin [38] and referred to as Cry1Ac-R [39]. The resistant strain Cry1Ac-R was then selected using 1000 μg/ml Cry1Ac protoxin in 2016 and reared on the artificial diet for over 50 generations without further insecticide exposure. We refer to this resistant strain as Cry1S1000. Both of the *P*. *xylostella* strains were kept at 26±1°C, 65% RH, and photoperiod of 16: 8 h (L: D). Adults were provisioned with 10% honey solution.

### Bioassays

Cry1Ac protoxin was prepared from *B*. *thuringiensis* subsp. *kurstaki* strain HD-73 (provided by Key Laboratory of Biopesticide and Chemical Biology, Ministry of Education, Fujian Agriculture and Forestry University) as previously described by Kain et al. [40] and serially diluted in ultrapure water. An artificial diet overlay assay, similar to that described by Zhao et al. [39] and Kain et al. [40], was used to determine the susceptibility of *P*. *xylostella* larvae to Cry1Ac. Bioassays were performed on five replicates for five different concentrations of Cry1Ac protoxin, plus an insecticide free control. For each replicate, pre-warmed liquid artificial diet (5 mL) was poured into a 30 mL plastic cup, and 0.3 mL aliquot of Cry1Ac protoxin was then evenly distributed over the surface of the diet (surface area ≈ 7 cm^2^). The assay was maintained at room temperature and after 1.5–2 hours of air-drying, ten early-molted 3^rd^-instar larvae were placed in the cup. Larval mortality was recorded at day 5, and mortality rate was calibrated according to Abbott’s formula [41].

### Cloning and sequencing of cDNA and genomic DNA

Midguts were dissected from G88 (n = 5) and Cry1S1000 (n = 5) 4^th^-instar larvae and total RNA was extracted from each individual using the TransZol Up Plus RNA Kit (TranGen, Beijing, China). cDNA synthesis was performed with the TransScript One-Step gDNA Removal and cDNA Synthesis Super Mix (TranGen, Beijing, China) according to the manufacturer’s instructions. PCR amplification of full length *PxABCC2* and *PxABCC3* transcripts were performed in a 25-μl reaction system containing 0.5 μl of cDNA template, 0.4 μM of primer (S11 Table), 12.5 μl of 2 × Phanta Max Buffer, and 0.5 μl of Phanta Max Super-Fidelity DNA Polymerase (Vazyme, Nanjing, China). Reactions were carried out with an initial denaturation at 95°C for 3 min and followed by 35 cycles of denaturation at 95°C for 15 s, annealing at 55°C for 15 s and extension at 72°C for 5 min. After examination by agarose gel electrophoresis, the amplified products were purified using the Gel Extraction Kit (Omega, Morgan Hill, GA, USA), cloned into the pJET1.2/blunt Cloning Vector using the CloneJET PCR Cloning Kit (Thermo Scientific, Waltham, MA, USA), and sequenced by Biosune Biotech Company (Fuzhou, China).

Genomic DNA (gDNA) was isolated from each of the individual carcasses remaining after midgut dissection using the Tissue DNA Kit (Omega, Morgan Hill, GA, USA). The gDNA was used as template for PCR amplification of four *PxABCC2* fragments (gDNA_PF1—PF4). Amplification reactions (25 μl) contained 50 ng of gDNA template, 0.4 μM of primer (S11 Table), 12.5 μl of 2 × Phanta Max Buffer, and 0.5 μl of Phanta Max Super-Fidelity DNA Polymerase (Vazyme, Nanjing, China). All fragments were amplified with the extension time adjusted according to the fragment size, then cloned and sequenced as previously described.

### Allele-specific PCR assay

Based on the size variation of *PxABCC2* intron 6 and *PxABCC3* intron 13 between Cry1S1000 and G88, to detect resistant (*R*_*A2*_ or *R*_*A3*_) alleles, we used allele-specific PCR and two pairs of primers (S11 Table) that flank either *R*_*A2*_ or *R*_*A3*_ mutations. All adults that needed to be genotyped for *R*_*A2*_ or *R*_*A3*_ allele were sacrificed and gDNA was isolated individually using the Tissue DNA Kit (Omega, Morgan Hill, GA, USA). PCR amplification was performed in a 25-μl reaction system under the conditions described in section [Cloning and sequencing of cDNA and genomic DNA]. PCR products and DL1000 DNA marker (TAKARA, Dalian, China) were separated using 3% agarose gel electrophoresis to differentiate the size of DNA bands of resistant (*R*_*A2*_ and *R*_*A3*_) and susceptible (*S*_*A2*_ and *S*_*A3*_) alleles. To validate allele-specific PCR, amplified products were cloned and sequenced from Cry1S1000 (n = 5) and G88 (n = 5) as described in section [Cloning and sequencing of cDNA and genomic DNA].

### Genetic linkage analysis of *R*_*A2*_ and *R*_*A3*_ with Cry1Ac resistance

To test for genetic linkage between Cry1Ac resistance in the Cry1S1000 strain and *R*_*A2*_ and *R*_*A3*_, a single-pair cross was performed between a Cry1S1000 female and G88 male to generate F_1_ progeny. Ten single pair backcrosses (BC) were performed by crossing an F_1_ male and Cry1S1000 female (BCa1—BCa5) or an F_1_ female and Cry1S1000 male (BCb1—BCb5). For each backcross family, 36–48 3^rd^-instar larvae were reared on the artificial diet (control), while 50 larvae were reared on artificial diet supplemented with 0.5 μg/ml of Cry1Ac protoxin for five days. Mortality was recorded and all surviving larvae were allowed to complete development before gDNA was isolated.

### Introgression of the *R*_*A2*_ and *R*_*A3*_ alleles from Cry1S1000 into G88

The Cry1S1000 Cry1Ac resistance locus was introgressed into the genetic background of G88. First, males from Cry1S1000 (*R*_*A2*_*R*_*A3*_/*R*_*A2*_*R*_*A3*_) were mass crossed with G88 females (*S*_*A2*_*S*_*A3*_/*S*_*A2*_*S*_*A3*_) to produce heterozygous F_1_’s (*R*_*A2*_*R*_*A3*_/*S*_*A2*_*S*_*A3*_) and the male progeny were then mass crossed with G88 females to generate BC1 progeny. BC1 males were individually crossed with G88 females (single-pair mating) to generate BC2 progeny and sacrificed after mating for genotyping using allele-specific PCR assays. Only lines containing an *R*_*A2*_ allele (*R*_*A3*_ was not genotyped during introgression crosses as the two genes are tightly linked) were retained. Additional four single-pair backcrosses (BC3—BC6) and corresponding molecular assays were performed using the same method of backcrossing between BC1 males and G88 females. Subsequently, BC6 siblings were crossed in single pairs to generate BC6F_1_ progeny and lines containing heterozygous parents (*R*_*A2*_*S*_*A2*_) were retained after genotyping. Similarly, BC6F_1_ siblings were crossed (single-pair mating) to generate BC6F_2_ progeny and genotyped. Only lines containing homozygous parents (*R*_*A2*_*R*_*A2*_) were retained and constituted a homozygous strain of G88-R_A2_ (S16 Fig). Finally, the G88-R_A2_ strain was genotyped to confirm it was fixed for the *R*_*A3*_ allele.

### Determination of inheritance mode of Cry1Ac resistance

Reciprocal crosses were made between each of two resistant strains (Cry1S1000 and G88-R_A2_) and G88 to generate F_1_ heterozygotes using 15 virgin females from one strain and 15 males from the other. Bioassays were performed using 0.5 μg/ml Cry1Ac protoxin on Cry1S1000, G88-R_A2_, G88 and their F_1_ progeny from reciprocal crosses. To evaluate maternal effects and sex linkage of resistance in Cry1S1000 and G88-R_A2_, we compared the survival of F_1_ progeny from reciprocal crosses between Cry1S1000 and G88, and between G88-R_A2_ and G88. Dominance (*h*), ranging from 0 (completely recessive resistance) to 1 (completely dominant resistance), was estimated based on the survival of each resistant strain (Cry1S1000 and G88-R_A2_), G88 and their F_1_ progeny using the single-concentration method [42].

### Preparation of sgRNA and Cas9

sgRNA target sites containing 5'-N20NGG-3' (with the PAM sequence underlined) were selected in *PxABCC2* exons 1, 3, and 20 and in *PxABCC3* exon 1. A template for *in vitro* transcription of sgRNA’s was obtained using PCR that included two oligonucleotides. The first contained the T7 polymerase binding site and sgRNA target sequence indicated by twenty underlined N’s (5′-TAATACGACTCACTATAGGNNNNNNNNNNNNNNNNNNNNGTTTTAGAGCTAGAAATAGCAAGTTAA-3′), and the second contained the remaining sequence common to all sgRNAs (5′-AAAAGCACCGACTCGGTGCCACTTTTTCAAGTTGATAACGGAC TAGCCTTATTTTAACTTGCTATTTCTAGCTCTAAAA-3′). All sgRNA target sequences for *PxABCC2* and *PxABCC3* are listed in S12 Table. PCR amplification was performed in a 50-μl reaction system containing 0.4 μM of primer, 5 μl of 10× PCR buffer for KOD-Plus-Neo, 5 μl of 2 mM dNTPs, 3 μl of 25 mM MgSO_4_, and 1 μl of KOD-Plus-Neo (TOYOBO, Osaka, Japan) with the following program: 98°C for 2 min, 35 cycles at 98°C for 10 s, 55°C for 30 s, and 68°C for 30 s. The PCR products were examined by agarose gel electrophoresis and purified using the Gel Extraction Kit (Omega, Morgan Hill, GA, USA). sgRNAs were generated via *in vitro* transcription using the HiScribe T7 Quick High Yield RNA Synthesis Kit (New England Biolabs, Ipswich, MA, USA) and purified through phenol-chloroform extraction and ethanol precipitation. sgRNAs were then stored at -80°C until usage.

Cas9 mRNA was generated by *in vitro* transcription using the HiScribe T7 ARCA mRNA Kit (with tailing) (New England Biolabs, Ipswich, MA, USA) with the template of a linearized PTD1-T7-Cas9 vector [43], and purified as described for sgRNA. Cas9 protein (GenCrispr Cas9-N-NLS Nuclease) was purchased from the GeneScript Corporation (Nanjing, China).

### Microinjection of *P*. *xylostella* embryos

A parafilm sheet (5 cm × 15 cm) precoated with dry radish seedling powder was provided to G88 females for 15–20 minutes to facilitate egg collection. The parafilm sheet was retrieved and fixed to a glass slide to inject the embryos with a mixture containing Cas9 (300 ng/μl of Cas9 mRNA or 600 ng/μl of Cas9 protein) and 100 ng/μl of sgRNA. Injections were performed within 1–2 hours of lay egg collection using the IM 300 Microinjector (Narishige, Tokyo, Japan) mounted on the SZX16 Stereo Microscope (Olympus, Tokyo, Japan). Injected embryos were incubated without light at 26±1°C in a Petri dish containing a moist tissue paper until hatching. Hatched 1^st^-instar larvae were transferred to the artificial diet (representing the G_0_).

### Screening and development of homozygous mutant strains

Virgin G_0_ adults that survived microinjection were individually sexed and coupled with virgin G88 adults for single-pair mating. After successful oviposition, G_0_ individuals were sacrificed and gDNA isolated. PCR amplicons spanning sgRNA target sites were generated then sequenced from the G_0_’s, and only lines containing mutations were retained. G_1_ siblings were crossed in single pairs to produce G_2_ progeny and only lines that contain heterozygous parents sharing the same allelic mutation based on molecular identification were retained. Finally, G_2_ siblings were crossed (single-pair mating) again to generate G_3_ progeny and genotyped. Only lines containing homozygous mutant parents were kept to generate the homozygous mutant strains. If homozygous individuals could not be obtained from the G_2_ progeny, additional sibling crosses were performed (S17 Fig).

Identification of CRISPR/Cas9 mediated mutations first required isolating gDNA from whole adults after mating using the Tissue DNA Kit (Omega, Morgan Hill, GA, USA). Amplicons spanning the sgRNA target sites for *PxABCC2* and *PxABCC3* were generated using primers listed in S11 Table. PCR amplification was performed as described in section [Cloning and sequencing of cDNA and genomic DNA] and products were Sanger sequenced (Biosune Biotech Company, Fuzhou, China).

### Preparation of midgut brush border membrane vesicles (BBMV)

BBMV were prepared using the differential magnesium precipitation method [44]. The 4^th^-instar larvae were dissected longitudinally in cold buffer A (300 mM mannitol, 5 mM EGTA, 17 mM Tris-HCl, pH 7.5) on ice to isolate the midgut epithelium. For each strain analyzed, 10 dissected midguts were pooled and homogenized in 750 μl of buffer A containing 1 mM PMSF using the TissueLyser II mixer (Qiagen, Dusseldorf, Germany) and a 3 mm stainless-steel bead at 22 Hz for two 1-min periods, separated by a 1-min cooling interval on ice. Subsequently, an equal volume of 24 mM MgCl_2_ solution was added to the midgut homogenate and mixed. The mixture was incubated on ice for 15 min, and then centrifuged at 2,500 g for 15 min at 4°C. The supernatant was collected and stored on ice. A second extraction was performed by resuspending the pellet using half the volumes of cold buffer A solution, then the same process as the first extraction with equal volume of 24 mM MgCl_2_. The second 2,500 g supernatant was combined with the first one and centrifuged at 30,000 g for 30 min at 4°C. The final pellet was resuspended in 50 μl of cold half-strength buffer A. Protein concentrations were measured by the BCA assay kit (Solarbio, Beijing, China) with bovine serum albumin (BSA) as a standard.

### Protein identification

PxABCC2 protein from the midgut BBMV was determined by western blotting. BBMV proteins (20 μg) were separated using 7.5% SDS-PAGE and transferred onto polyvinylidene difluoride (PVDF) membranes (Millipore, Bedford, MA, USA) using transfer buffer (192 mM Glycine, 24.8 mM Tris, 20% methanol). Subsequently, the membranes were blocked in PBS (137 mM NaCl, 2.7 mM KCl, 10 mM Na_2_HPO_4_, 1.76 mM KH_2_PO_4_, pH 7.4) containing 4% BSA at 4°C overnight. Membranes were probed separately with antibodies in PBS containing 1% BSA for 1 hour. All incubation steps were performed on a TS-100 shaker (Qilinbeier, Haimen, China) at room temperature and PVDF membranes were washed four times (5 min each time) with PBST (PBS containing 0.25% Tween-20) between steps. The antibodies were rabbit polyclonal anti-PxABCC2 antibody (1: 1,000 dilution, ABclonal, Wuhan, China) and anti-rabbit IgG (1: 10,000 dilution, Affinity, Cincinnati, OH, USA), or mouse monoclonal anti-alpha-tubulin (control, 1: 5000 dilution, Sigma-Aldrich, Saint Louis, MO, USA) and anti-mouse IgG (1: 50,000 dilution, Sigma, Saint Louis, MO, USA). Membranes were visualized with the Clarity Western ECL Substrate (Bio-Rad, Hercules, CA, USA).

PxABCC2 protein was further confirmed by nano-liquid chromatography-tandem mass spectrometry (nano-LC-MS/MS). Since PxABCC3 lacked primary antibody for the western blotting, its protein from the midgut BBMV was identified by nano-LC-MS/MS. Total BBMV proteins (30 μg) were separated on 7.5% SDS-PAGE. The regions expected to contain PxABCC2 and PxABCC3 proteins (~150 kDa) were excised from Coomassie blue stained gels, preserved in PBS buffer and shipped with ice packs to Huada Protein Research Center (Shenzhen, China) for tryptic digestion and analysis by nano-LC-MS/MS.

### Allelic complementation test

Allelic complementation tests were performed between Cry1S1000 and introgression strain G88-R_A2_ using 10 single-pair reciprocal crosses and challenged their F_1_ progeny (25 to 50 larvae per family) with 0.5 μg/ml Cry1Ac protoxin. To determine whether the homozygous mutations in *PxABCC2* or *PxABCC3* alone or combined together confer Cry1Ac resistance, we performed 8 single-pair reciprocal crosses between three CRISPR/Cas9 lines and Cry1S1000, G88-R_A2_ or G88. Subsequently, the F_1_ progeny from each of these 8 single-pair crosses (20 to 45 larvae per family), along with six parental strains (three CRISPR/Cas9 lines, Cry1S1000, G88-R_A2_ and G88; 50 to 60 larvae per strain), were assayed with 0.5 μg/ml Cry1Ac protoxin. We then used the surviving individuals to calculate the index of commonality (*C*), which varies from close to or < 0 (resistance conferred by alleles at different loci) to close to or > 1 (resistance conferred by alleles at a shared locus or loci) [45].

In another additional experiment, each of three CRISPR/Cas9 mutant lines were mass crossed with G88-R_A2_ (mass mating, 15 pairs) to generate F_1_ progeny. These F_1_ progeny individuals were tested with at least five concentrations of Cry1Ac protoxin to get the LC_50_ using artificial diet overlay assay as described in section [Bioassays].

### Data analyses

All statistical analyses were performed using IBM SPSS Statistics 22. LC_50_ values for Cry1Ac larval bioassays were calculated using probit analysis. Significant differences in the LC_50_ values were defined by non-overlapping 95% confidence limits [46]. Fisher’s exact test was used to determine significant differences between observed and expected genotypes among genetic crosses.

## Supporting information

S1 Table Susceptibility of different *P*. *xylostella* strains to Cry1Ac protoxin. (DOC)

S2 Table Resistant response of the Cry1S1000 strain to Cry1Ac protoxin at different generations. (DOC)

S3 Table Genetic linkage of the *R*_*A2*_ and *R*_*A3*_ alleles with Cry1Ac resistance. (DOC)

S4 Table Genotype of the *R*_*A2*_ and *R*_*A3*_ alleles in *P*. *xylostella* during introgression. (DOC)

S5 Table Inheritance of Cry1Ac resistance in the Cry1S1000 and G88-R_A2_ strains of *P*. *xylostella*. (DOC)

S6 Table Targeted mutagenesis of *PxABCC2* and *PxABCC3* induced by CRISPR/Cas9. (DOC)

S7 Table A total of 1,179 peptides identified from the G88-ABCC2^-^-1 mutant strain. (XLS)

S8 Table A total of 2,553 peptides identified from the G88-ABCC3^-^-1 mutant strain. (XLS)

S9 Table A total of 1,968 peptides identified from the G88-ABCC3^-^-2 mutant strain. (XLS)

S10 Table A total of 3,159 peptides identified from the G88-A2^-^A3^-^-2 mutant strain. (XLS)

S11 Table Sequences of primers used to amplify *PxABCC2* and *PxABCC3* in this study. (DOC)

S12 Table Target sequences of sgRNAs for *PxABCC2* and *PxABCC3*. (DOC)

S1 Fig Alignment of *PxABCC2* cDNA sequences from the G88 and Cry1S1000 strains. Sequence alignment of *PxABCC2* cDNA from G88 (ABCC2_S, GenBank accession no. MN652064) and five isoforms from Cry1S1000 (ABCC2_R1-5, GenBank accession no. MN652066-MN652070). Asterisks denote consensus sequences. Exon regions (yellow and green) are shown in the ABCC2_S. Deletions and insertions are respectively highlighted in gray and blue. Codons highlighted in red indicate the premature stop codons.
(DOC)

S2 Fig Alignment of predicted amino acid sequences of PxABCC2 from G88 (ABCC2_S) and Cry1S1000 (ABCC2_R1-5). Asterisks denote consensus sequences. Transmembrane domains (TM) (blue), Walker A and B sequences (yellow), C motif (green) and region for antibody recognition (underlined) are shown in the sequence of PxABCC2 protein encoded by ABCC2_S. Deletions are highlighted in gray.
(DOC)

S3 Fig Alignment of partial gDNA sequence (gDNA_PF1, GenBank accession no. MN660237) of *PxABCC2* and partial sequence of ABCC2_R1 cDNA from Cry1S1000. Asterisks denote consensus sequences. Exons (yellow) and introns (gray) are shown in the sequence of gDNA_PF1. Deletions in cDNA are highlighted in green. Letter highlighted in red indicates the point mutation in gDNA. The primer sequences are underlined in red.
(DOC)

S4 Fig Alignment of partial gDNA sequence (gDNA_PF2, GenBank accession no. MN660238) of *PxABCC2* and partial sequences of ABCC2_R2/4 cDNA from Cry1S1000. Asterisks denote consensus sequences. Exons (yellow) and introns (gray) are shown in the fragment sequence of gDNA_PF2. Deletions and insertions in cDNA are respectively highlighted in green and blue. The primer sequences are underlined in red.
(DOC)

S5 Fig Alignment of partial gDNA sequence (gDNA_PF3, GenBank accession no. MN660239) of *PxABCC2* and partial sequences of ABCC2_R3/4 cDNA from Cry1S1000. Exons (yellow) and introns (gray) are shown in the sequence of gDNA_PF3. Insertions in cDNA are highlighted in blue. The primer sequences are underlined in red.
(DOC)

S6 Fig Alignment of partial gDNA sequence (gDNA_PF4, GenBank accession no. MN660240) of *PxABCC2* and partial sequence of ABCC2_R4 cDNA from Cry1S1000. Asterisks denote consensus sequences. Exons (yellow) and introns (gray) are shown in the fragment sequence of gDNA_PF4. Insertions in cDNA are highlighted in blue. The primer sequences are underlined in red.
(DOC)

S7 Fig *R*_*A2*_ mutation in intron 6 of Cry1S1000 *PxABCC2*. (**A**) Genotyping of *PxABCC2* in G88 and Cry1S1000 individuals using AS-PCR. (**B**) Alignment of gDNA sequences of the *S*_*A2*_ allele (GenBank accession no. MN660243) and the *R*_*A2*_ allele (GenBank accession no. MN660241). Letter in red indicates the point mutation in 3′ splice site of intron 6. Two sequences highlighted in orange are the primer of 33fPxABCC2, and a sequence matched by the primer of 55PxABCC2.
(TIF)

S8 Fig Alignment of *PxABCC3* cDNA sequences from the strains of G88 (ABCC3_S, GenBank accession no. MN652065) and Cry1S1000 (ABCC3_R, GenBank accession no. MN652071). Asterisks denote consensus sequences. Codons highlighted in red indicate premature stop codon.
(DOC)

S9 Fig Alignment of predicted amino acid sequences of PxABCC3 from the G88 and Cry1S1000 strains. Asterisks denote consensus sequences. Transmembrane domains (TM) (blue), Walker A and B sequences (yellow), and C motif (green) are shown in the sequence of PxABCC3 protein encoded by the susceptible allele. Transmembrane helices, except for TM1 and TM2, were predicted by Phobius.
(DOC)

S10 Fig *R*_*A3*_ mutation in exon 14 of Cry1S1000 *PxABCC3*. (**A**) Genotyping of *PxABCC3* in G88 and Cry1S1000 individuals using AS-PCR. (**B**) Alignment of gDNA sequences of the *S*_*A3*_ allele (GenBank accession no. MN660244) and the *R*_*A2*_ allele (GenBank accession no. MN660242). Letter in red indicates the point mutation in exon 14. Two sequences highlighted in orange are the primer of 58PxABCC3, and a sequence matched by the primer of 37rPxABCC3.
(TIF)

S11 Fig Representative sequencing trace of the PCR fragments from wild type (G88), heterozygotes and homozygous mutant strains containing sgRNA target sites 1 (A), 2 (B) and 3 (C) for *PxABCC2*, respectively. (TIF)

S12 Fig Protein identification of PxABCC2 in different *P*. *xylostella* strains. (**A**) Analysis of PxABCC2 in the wild type G88 strain, four *PxABCC2* knockout strains and G88-No-Indel using the western blotting. PxABCC2 antibody recognizes a region from extracellular loop 3 to partial nucleotide-binding domain 1 as indicated in the amino acid sequence of full-length PxABCC2 protein (S2 Fig). (**B**) SDS-PAGE profile of midgut BBMV protein from G88 and one *PxABCC2* knockout strain, G88-ABCC2^-^-1. The dashed rectangles indicate the region of SDS-PAGE gel excised and analyzed by nano-LC-MS/MS. (**C**) Details of 34 peptides specific to PxABCC2 identified from the G88 strain. None of PxABCC2 peptides were detected from the G88-ABCC2^-^-1 strain. (**D**) Map of the full-length PxABCC2 protein showing the position of 31 peptides (red arrows) specific to PxABCC2 identified from the G88 strain. Black arrow indicates where the protein of G88-ABCC2^-^-1 is expected to be truncated. The predicted tryptic fragments (no detection from the mutant strain using nano-LC-MS/MS analysis) for expected truncated PxABCC2 from G88-ABCC2^-^-1 are indicated with blue arrows. Numbers indicate the position of amino acid residues.
(TIF)

S13 Fig Representative sequencing trace of the PCR fragments containing sgRNA target sites for *PxABCC3* in *PxABCC3* mutant strains. (TIF)

S14 Fig Protein identification of PxABCC3 in different *P*. *xylostella* strains. (**A**) SDS-PAGE profile of BBMV protein from the strain of G88 and two *PxABCC3* knockout strains of G88-ABCC3^-^-1 and G88-ABCC3^-^-2. The dashed rectangles indicate the region of SDS-PAGE gel excised and analyzed by nano-LC-MS/MS. (**B**) Details of the 33 peptides specific to PxABCC3 identified from the G88 strain. None of PxABCC3 peptides were detected from two *PxABCC3* knockout strains. (**C**) Map of the full-length PxABCC3 protein showing the position of 33 peptides (red arrows) specific to PxABCC3 identified from the G88 strain. Black arrows indicate where the proteins of G88-ABCC3^-^-1 and G88-ABCC3^-^-2 are expected to be truncated. The predicted tryptic fragments (no detection from two mutant strains using nano-LC-MS/MS analysis) for expected truncated PxABCC3 proteins from G88-ABCC3^-^-1 and G88-ABCC3^-^-2 are indicated with blue arrows. Numbers indicate the position of amino acid residues.
(TIF)

S15 Fig Protein identification of PxABCC2 and PxABCC3 in different *P*. *xylostella* strains. (**A**) SDS-PAGE profile of BBMV protein from the G88 strain and one *PxABCC2*/*PxABCC3* double mutant strain, G88-A2^-^A3^-^-1. The dashed rectangles indicate the region of SDS-PAGE gel excised and analyzed by nano-LC-MS/MS. (**B**) Details of the 27 peptides specific to PxABCC2 identified from the G88 strain. None of PxABCC2 peptides were detected from the G88-A2^-^A3^-^-1 strain. (**C**) Map of the full-length PxABCC2 protein showing the position of 28 peptides (red arrows) specific to PxABCC2 identified from the G88 strain. Black arrow indicates where the protein of G88-A2^-^A3^-^-1 is expected to be truncated. The predicted tryptic fragments (no detection from the mutant strain using nano-LC-MS/MS analysis) for expected truncated PxABCC2 proteins from G88-A2^-^A3^-^-1 are indicated with blue arrows. Numbers indicate the position of amino acid residues. (**D**) Details of the 40 peptides specific to PxABCC3 identified from the G88 strain. None of PxABCC3 peptides were detected from the G88-A2^-^A3^-^-1 strain. (**E**) Map of the full-length PxABCC3 protein showing the position of 40 peptides (red arrows) specific to PxABCC3 identified from the G88 strain. Black arrow indicates where the protein of G88-A2^-^A3^-^-1 is expected to be truncated. The predicted tryptic fragments (no detection from the mutant strain using nano-LC-MS/MS analysis) for expected truncated PxABCC3 proteins from G88-A2^-^A3^-^-1 are indicated with blue arrows. Numbers indicate the position of amino acid residues.
(TIF)

S16 Fig Schematic strategy of continuous backcrossing for introgression of *R*_*A2*_ and *R*_*A3*_ alleles from the resistant Cry1S1000 strain into the genetic background of the susceptible G88 strain of *P*. *xylostella*. Square and circle represent a male adult and a female adult, respectively. BC1-6: 1–6 generations of backcrossing. BC6F1 and BC6F2 are two additional generations for sibling crosses in single pair to generate a homozygous strain with *R*_*A2*_*R*_*A2*_ (G88-R_A2_). *S*_*A2*_*S*_*A2*_/*R*_*A2*_*S*_*A2*_ underlined with the red line represent the filtered individuals of each generation after molecular identification using AS-PCR.
(TIF)

S17 Fig Schematic strategy for screening of homozygous mutant strains. Each rectangle represents a paired female and male. Red rectangles represent the single-pair families kept for the following generations whose parent(s) harbored the desire frameshift mutations as determined by PCR and direct sequencing. “A” shown in the rectangles is the wild type allele; “a_1_”, “a_2_” and “a_3_” are mutant alleles with different type of indel mutations.
(TIF)
