# Supplementary material for: Resistance to Bacillus thuringiensis Cry1Ac toxin requires mutations in two Plutella xylostella ATP-binding cassette transporter paralogs
Source: PLoS Pathog. 2020 Aug 10;16(8):e1008697. doi: 10.1371/journal.ppat.1008697 (PMC7446926; doi:10.1371/journal.ppat.1008697)
Supplement: S1 Table — (DOC) [file ppat.1008697.s001.doc]

**S1 Table. Susceptibility of different *P. xylostella* strains to Cry1Ac protoxin.**

| Bioassay | *P. xylostella* strain | N*^a^* | Slope (SE) | LC_50_*^b^* (μg/ml) (95% CL*^c^*) | RR*^d^* |
| --- | --- | --- | --- | --- | --- |
| Bioassay 1 | G88 | 300 | 4.487 (0.548) | 0.121 (0.102-0.144) | 1 |
|  | Cry1S1000 | 300 | - | > 1,000 | > 8,000 |
|  | G88-R_A2_ | 300 | - | > 1,000 | > 8,000 |
|  | G88- A2^-^A3^-^-1 | 350 | - | > 1,000 | > 8,000 |
|  | G88- A2^-^A3^-^-2 | 350 | - | > 1,000 | > 8,000 |
|  | G88- A2^-^A3^-^-3 | 350 | - | > 1,000 | > 8,000 |
|  | F_1_ (G88- A2^-^A3^-^-1 × G88-R_A2_) | 350 | - | > 1,000 | > 8,000 |
| Bioassay 2 | G88 | 300 | 3.403 (0.356) | 0.116 (0.101-0.135) | 1 |
|  | G88-No-Indel | 300 | 3.376 (0.341) | 0.106 (0.070-0.156) | 0.91 |
|  | G88-ABCC2^-^-1 | 300 | 4.397 (0.512) | 0.295 (0.260-0.335) | 2.54 |
|  | G88-ABCC2^-^-2 | 350 | 3.850 (0.419) | 0.241 (0.211-0.277) | 2.08 |
|  | G88-ABCC2^-^-3 | 300 | 3.301 (0.430) | 0.312 (0.267-0.374) | 2.69 |
|  | G88-ABCC2^-^-4 | 350 | 2.682 (0.330) | 0.438 (0.366-0.543) | 3.78 |
| Bioassay 3 | G88 | 350 | 2.836 (0.269) | 0.080 (0.069-0.095) | 1 |
|  | G88-ABCC3^-^-1 | 350 | 5.064 (0.760) | 0.021 (0.018-0.024) | 0.26 |
|  | G88-ABCC3^-^-2 | 350 | 2.939 (0.340) | 0.031 (0.026-0.036) | 0.39 |
| Bioassay 4 | G88 | 350 | 3.343 (0.337) | 0.05 (0.043-0.058) | 1 |
|  | F_1_ (G88-ABCC2^-^-1 × G88-R_A2_) | 300 | 2.673 (0.292) | 0.154 (0.126-0.184) | 3.08 |
|  | F_1_ (G88-ABCC3^-^-1 × G88-R_A2_) | 300 | 3.291 (0.374) | 0.041 (0.033-0.049) | 0.82 |

*^a^* Number of 3^rd^-instar larvae tested.

*^b^* Median lethal concentration.

*^c^* 95% confidence limit.

*^d^* Resistance ratio = LC_50_ of each tested strain / LC_50_ of the susceptible G88 strain.
