# Supplementary material for: Resistance to Bacillus thuringiensis Cry1Ac toxin requires mutations in two Plutella xylostella ATP-binding cassette transporter paralogs
Source: PLoS Pathog. 2020 Aug 10;16(8):e1008697. doi: 10.1371/journal.ppat.1008697 (PMC7446926; doi:10.1371/journal.ppat.1008697)
Supplement: S2 Table — (DOC) [file ppat.1008697.s002.doc]

**S2 Table. Resistant response of the Cry1S1000 strain to Cry1Ac protoxin at different generations.**

| Generation | N*^a^* | LC_50_*^b^* (μg/ml) | N*^c^* | Survival*^d^* (%) |
| --- | --- | --- | --- | --- |
| 2 | 240 | > 1,000 | - | - |
| 8 | 300 | > 1,000 | - | - |
| 12 | 300 | > 1,000 | - | - |
| 13 | - | - | 50 | 100 |
| 26 | 400 | > 1,000 | - | - |
| 31 | - | - | 50 | 100 |
| 38 | - | - | 50 | 100 |
| 40 | 300 | > 1,000 | - | - |
| 46 | - | - | 50 | 100 |

*^a^* Number of 3^rd^-instar larvae tested for determination of LC_50_.

*^b^* Median lethal concentration.

*^c^* Number of 3^rd^-instar larvae tested for determination of survival at the diagnostic concentration of Cry1Ac protoxin.

*^d^* Survival at the diagnostic concentration (0.5 μg/ml) of Cry1Ac protoxin.
