# Supplementary material for: Resistance to Bacillus thuringiensis Cry1Ac toxin requires mutations in two Plutella xylostella ATP-binding cassette transporter paralogs
Source: PLoS Pathog. 2020 Aug 10;16(8):e1008697. doi: 10.1371/journal.ppat.1008697 (PMC7446926; doi:10.1371/journal.ppat.1008697)
Supplement: S3 Table — (DOC) [file ppat.1008697.s003.doc]

**S3 Table. Genetic linkage of the *R_A2_* and *R_A3_* alleles with Cry1Ac resistance.**

| Backcross family*^a^* | Untreated control | | |  | Cry1Ac bioassay | | |
| --- | --- | --- | --- | --- | --- | --- | --- |
|  | N*^b^* | Larvae with *R_A2_R_A2_* (%) | Larvae with *R_A3_R_A3_* (%) |  | N*^c^* | Larvae with *R_A2_R_A2_* (%) | Larvae with *R_A3_R_A3_* (%) |
| BCa1 | 36 | 58.3 | 58.3 |  | 20 | 100 | 100 |
| BCa2 | 39 | 42.6 | 42.6 |  | 20 | 100 | 100 |
| BCa3 | 47 | 56.8 | 56.8 |  | 29 | 100 | 100 |
| BCa4 | 44 | 46.2 | 46.2 |  | 24 | 100 | 100 |
| BCa5 | 48 | 56.3 | 56.3 |  | 23 | 100 | 100 |
| BCb1 | 48 | 60.4 | 60.4 |  | 23 | 100 | 100 |
| BCb2 | 44 | 50.0 | 50.0 |  | 24 | 100 | 100 |
| BCb3 | 47 | 46.8 | 46.8 |  | 23 | 100 | 100 |
| BCb4 | 47 | 48.9 | 48.9 |  | 24 | 100 | 100 |
| BCb5 | 47 | 51.1 | 51.1 |  | 14 | 100 | 100 |
| Mean | - | 51.7 | 51.7 |  | - | 100 | 100 |
| Total | 447 | - | - |  | 224 | - | - |

*^a^* BCa1-5 families were produced by single-pair crosses between an F_1_ male (from Cry1S1000♀ × G88♂) and Cry1S1000 female; BCb1-5 families were produced by single-pair crosses between an F_1_ female (from Cry1S1000♀ × G88♂) and Cry1S1000 male.

*^b^* Number of untreated control larvae in each backcross family for genotyping.

*^c^* Number of survived larvae after treatment in each backcross family for genotyping. In the bioassay, 50 larvae from each of 10 backcross families (n = 500) were tested with 0.5 μg/ml of Cry1Ac protoxin.
