# Supplementary material for: Resistance to Bacillus thuringiensis Cry1Ac toxin requires mutations in two Plutella xylostella ATP-binding cassette transporter paralogs
Source: PLoS Pathog. 2020 Aug 10;16(8):e1008697. doi: 10.1371/journal.ppat.1008697 (PMC7446926; doi:10.1371/journal.ppat.1008697)
Supplement: S4 Table — (DOC) [file ppat.1008697.s004.doc]

**S4 Table. Genotype of the *R_A2_* and *R_A3_* alleles in *P. xylostella* during** introgression.

| Population | N*^a^* | Genotype | | | |
| --- | --- | --- | --- | --- | --- |
|  |  | *R_A2_R_A2_* | *R_A2_S_A2_* | *S_A2_S_A2_* | *R_A3_R_A3_* |
| BC1 | 20 | 0 | 20 | 0 | - |
| BC2 | 14 | 0 | 6 | 8 | - |
| BC3 | 24 | 0 | 12 | 12 | - |
| BC4 | 20 | 0 | 10 | 10 | - |
| BC5 | 16 | 0 | 11 | 5 | - |
| BC6 | 18 | 0 | 10 | 8 | - |
| BC6F1 | 62 | 0 | 33 | 29 | - |
| BC6F2 | 96 | 27 | 51 | 18 | - |
| G88-R_A2_ | 24 | 24 | 0 | 0 | 24 |

*^a^* Number of moths checked.
