# Supplementary material for: Resistance to Bacillus thuringiensis Cry1Ac toxin requires mutations in two Plutella xylostella ATP-binding cassette transporter paralogs
Source: PLoS Pathog. 2020 Aug 10;16(8):e1008697. doi: 10.1371/journal.ppat.1008697 (PMC7446926; doi:10.1371/journal.ppat.1008697)
Supplement: S5 Table — (DOC) [file ppat.1008697.s005.doc]

**S5 Table. Inheritance of Cry1Ac resistance in the Cry1S1000 and G88-R_A2_ strains of *P. xylostella*.**

| Population | N*^a^* | Survival*^b^* (%) | *h^c^* | *C^d^* |
| --- | --- | --- | --- | --- |
| Cry1S1000 | 50 | 100 | 0 | - |
| G88-R_A2_ | 50 | 100 | 0 | - |
| G88 | 50 | 0 | - | - |
| F_1_ (Cry1S1000♀ × G88♂) | 50 | 0 | - | - |
| F_1_ (G88♀ × Cry1S1000♂) | 50 | 0 | - | - |
| F_1_ (G88-R_A2_♀ × G88♂) | 50 | 0 | - | - |
| F_1_ (G88♀ × G88-R_A2_♂) | 50 | 0 | - | - |
| F_1_ (G88-R_A2_♀ × Cry1S1000♂) | 385 | 100 | - | 1 |

*^a^* Number of 3^rd^-instar larvae tested.

*^b^* Survival at the diagnostic concentration (0.5 μg/ml) of Cry1Ac protoxin.

*^c^* Parameter *h* indicates the degree of dominance, with a value of 0 representing completely recessive resistance.

*^d^* Index of commonality (*C*) indicates the proportion of two strains sharing the common resistance locus or loci, with a value of 1 representing resistance conferred by alleles at a shared locus or loci.
