# Supplementary material for: Resistance to Bacillus thuringiensis Cry1Ac toxin requires mutations in two Plutella xylostella ATP-binding cassette transporter paralogs
Source: PLoS Pathog. 2020 Aug 10;16(8):e1008697. doi: 10.1371/journal.ppat.1008697 (PMC7446926; doi:10.1371/journal.ppat.1008697)
Supplement: S6 Table — (DOC) [file ppat.1008697.s006.doc]

**S6 Table. Targeted mutagenesis of *PxABCC2* and *PxABCC3*** induced by CRISPR/Cas9.

| Strain for microinjection | Target gene | Target position | Cas9 source | sgRNA | Hatchability*^a^* | Survival rate*^b^* | Mosaic G_0_*^c^* |
| --- | --- | --- | --- | --- | --- | --- | --- |
| G88 | *PxABCC2* | Exon 1 | Cas9 protein | ABCC2-sg1 | 61.1% (55/90) | 81.8% (45/55) | 75.0% (18/24) |
| G88 | *PxABCC2* | Exon 3 | Cas9 mRNA | ABCC2-sg2 | 68.6% (96/140) | 63.5% (61/96) | 70.8% (17/24) |
| G88 | *PxABCC2* | Exon 20 | Cas9 mRNA | ABCC2-sg3 | 77.3% (75/97) | 38.7% (29/75) | 85.7% (18/21) |
| G88 | *PxABCC3* | Exon 1 | Cas9 protein | ABCC3-sg1 | 78.9% (71/90) | 76.1% (54/71) | 7.0% (3/43) |
| G88-ABCC3^-^-1*^d^* | *PxABCC2* | Exon 3 | Cas9 protein | ABCC2-sg2 | 27.9% (12/43) | 50.0% (6/12) | 66.7% (4/6) |
| G88-ABCC3^-^-2*^d^* | *PxABCC2* | Exon 3 | Cas9 protein | ABCC2-sg2 | 51.9% (40/77) | 62.5% (25/40) | 60.0% (15/25) |

*^a^* Hatchability = number of hatched neonates / number of embryos injected.

*^b^* Survival rate = number of adults / number of hatched neonates.

*^c^* Percentage of mosaic G_0_ = number of mutated G_0_ moths among the G_0_ moths detected / number of total G_0_ moths randomly selected for detection.

*^d^* Mutant strains in which *PxABCC3* was knocked out.
