# Supplementary material for: Resistance to Bacillus thuringiensis Cry1Ac toxin requires mutations in two Plutella xylostella ATP-binding cassette transporter paralogs
Source: PLoS Pathog. 2020 Aug 10;16(8):e1008697. doi: 10.1371/journal.ppat.1008697 (PMC7446926; doi:10.1371/journal.ppat.1008697)
Supplement: S11 Table — (DOC) [file ppat.1008697.s011.doc]

**S11 Table. Sequences of primers used to amplify *PxABCC2* and *PxABCC3* in this study.**

| Primer | Sequence (5′-3′) | Direction | Application |
| --- | --- | --- | --- |
| 34fPxABCC2 | ATGGAAAACGGAAGCGGAG | Sense | cDNA PCR amplification of full-length *PxABCC2* CDS  gDNA amplification of partial fragment containing ABCC2-sg1 |
| 3PxABCC2 | TTGAGGATGGTCATCGAAG | Antisense | cDNA PCR amplification of full-length *PxABCC2* CDS |
| 32fPxABCC2 | GCACAAAGGACAGATAAGAG | Sense | gDNA PCR amplification of partial fragment (gDNA_PF1) of *PxABCC2* |
| 39rPxABCC2 | CGTGGTCTCCTTCGTGTA | Antisense | gDNA PCR amplification of partial fragment (gDNA_PF1) of *PxABCC2* |
| 41fPxABCC2 | ACCTCAAGTGGGAGGTGTTC | Sense | gDNA PCR amplification of partial fragment (gDNA_PF2) of *PxABCC2* |
| 41rPxABCC2 | ACGATKATGGTCAGCACCAG | Antisense | gDNA PCR amplification of partial fragment (gDNA_PF2) of *PxABCC2* |
| 71fPxABCC2 | ACTTCAAGCAGTTCCCGTA | Sense | gDNA PCR amplification of partial fragment (gDNA_PF3) of *PxABCC2* |
| 71rPxABCC2 | CAGCAGCGAATTCTCCAG | Antisense | gDNA PCR amplification of partial fragment (gDNA_PF3) of *PxABCC2* |
| 72fPxABCC2 | CCAACATCCACGACCTCA | Sense | gDNA PCR amplification of partial fragment (gDNA_PF4) of *PxABCC2* |
| 72rPxABCC2 | TACCCTCCAGCCTCTTCA | Antisense | gDNA PCR amplification of partial fragment (gDNA_PF4) of *PxABCC2* |
| 33fPxABCC2 | CATGGCTACTGCTACTACG | Sense | Allele specific PCR for detection of the *R_A2_* mutation |
| 55PxABCC2 | CTTTCCAATGAAACCAAC | Antisense | Allele specific PCR for detection of the *R_A2_* mutation |
| 34PxABCC2 | TCAACCACGACCGAAATAG | Antisense | gDNA PCR amplification of partial fragment containing ABCC2-sg1 |
| 1PxABCC2 | TGCACACTGCCAGTCGTCTC | Sense | gDNA PCR amplification of partial fragment containing ABCC2-sg2 |
| 2PxABCC2 | GCCCGCGTACAGCTTCATTC | Antisense | gDNA PCR amplification of partial fragment containing ABCC2-sg2 |
| 13fPxABCC2 | TGTCAGCATCAGCTTCAG | Sense | gDNA PCR amplification of partial fragment containing ABCC2-sg3 |
| 13rPxABCC2 | GTGCGGCAGTTTAGTGTA | Antisense | gDNA PCR amplification of partial fragment containing ABCC2-sg3 |
| 17f2PxABCC3 | GAATGGGGGTGAAGGTTG | Sense | cDNA PCR amplification of full-length *PxABCC3* CDS |
| 17rPxABCC3 | TTTGTGGGAGGAGTGGGT*^a^* | Antisense | cDNA PCR amplification of full-length *PxABCC3* CDS |
| 58PxABCC3 | GGTAAAGTGGAGAATATGGG | Sense | Allele specific PCR for detection of the *R_A3_* mutation |
| 37rPxABCC3 | ACAACAGTCAGGAACAGAG | Antisense | Allele specific PCR for detection of the *R_A3_* mutation |
| 17f1PxABCC3 | AGGTTGCGGAGGATGTGT*^a^* | Sense | gDNA PCR amplification of partial fragment containing ABCC3-sg1 |
| 49PxABCC3 | GCACGATTTGACTACTTAGG | Antisense | gDNA PCR amplification of partial fragment containing ABCC3-sg1 |

*^a^* Primers reported by Guo et al. (PLoS Genet., 2015, 11: e1005124).
