# Supplementary material for: Resistance to Bacillus thuringiensis Cry1Ac toxin requires mutations in two Plutella xylostella ATP-binding cassette transporter paralogs
Source: PLoS Pathog. 2020 Aug 10;16(8):e1008697. doi: 10.1371/journal.ppat.1008697 (PMC7446926; doi:10.1371/journal.ppat.1008697)
Supplement: S12 Table — (DOC) [file ppat.1008697.s012.doc]

**S12 Table. Target sequences of sgRNAs for *PxABCC2* and *PxABCC3*.**

| sgRNA | Target position | Sequence (5′-3′) | Application |
| --- | --- | --- | --- |
| ABCC2-sg1 | Exon 1 | AAGTACAAGTCCGAGTCTCT | sgRNA target sequence for *PxABCC2* |
| ABCC2-sg2 | Exon 3 | CCCAATAGCCGGCCTCCAGT | sgRNA target sequence for *PxABCC2* |
| ABCC2-sg3 | Exon 20 | CAGCAGCACCGTCAGCACCA | sgRNA target sequence for *PxABCC2* |
| ABCC3-sg1 | Exon 1 | ATGTTCCCGCTGTTCTACCA | sgRNA target sequence for *PxABCC3* |
