# Supplementary material for: Resistance to Bacillus thuringiensis Cry1Ac toxin requires mutations in two Plutella xylostella ATP-binding cassette transporter paralogs
Source: PLoS Pathog. 2020 Aug 10;16(8):e1008697. doi: 10.1371/journal.ppat.1008697 (PMC7446926; doi:10.1371/journal.ppat.1008697)
Supplement: S1 Fig — Sequence alignment of PxABCC2 cDNA from G88 (ABCC2_S, GenBank accession no. MN652064) and five isoforms from Cry1S1000 (ABCC2_R1-5, GenBank accession no. MN652066-MN652070). Asterisks denote consensus sequences. Exon regions (yellow and green) are shown in the ABCC2_S. Deletions and insertions are respectively highlighted in gray and blue. Codons highlighted in red indicate the premature stop codons. (DOC) [file ppat.1008697.s013.doc]

**S1 Fig.**

ABCC2_S ATGGAAAACGGAAGCGGAGCCCGGAAAGAGTCGGAAGAGAAGAAAGAGGTGAAGAAGGGC

ABCC2_R1 ATGGAAAACGGAAGCGGAGCCCGGAAAGAGTCGGAAGAGAAGAAAGAGGTGAAGAAGGGC

ABCC2_R2 ATGGAAAACGGAAGCGGAGCCCGGAAAGAGTCGGAAGAGAAGAAAGAGGTGAAGAAGGGC

ABCC2_R3 ATGGAAAACGGAAGCGGAGCCCGGAAAGAGTCGGAAGAGAAGAAAGAGGTGAAGAAGGGC

ABCC2_R4 ATGGAAAACGGAAGCGGAGCCCGGAAAGAGTCGGAAGAGAAGAAAGAGGTGAAGAAGGGC

ABCC2_R5 ATGGAAAACGGAAGCGGAGCCCGGAAAGAGTCGGAAGAGAAGAAAGAGGTGAAGAAGGGC

************************************************************

**exon 1**

ABCC2_S AAGCCGAACGTGCTGTCCCGGCTGTTCATGTGCTGGGTGTGCCCCGTGCTGGTGGGCGGC

ABCC2_R1 AAGCCCAACGTGCTGTCCCGGCTGTTCATGTGCTGGGTGTGCCCCGTGCTGGTGGGCGGC

ABCC2_R2 AAGCCCAACGTGCTGTCCCGGCTGTTCATGTGCTGGGTGTGCCCCGTGCTGGTGGGCGGC

ABCC2_R3 AAGCCCAACGTGCTGTCCCGGCTGTTCATGTGCTGGGTGTGCCCCGTGCTGGTGGGCGGC

ABCC2_R4 AAGCCCAACGTGCTGTCCCGGCTGTTCATGTGCTGGGTGTGCCCCGTGCTGGTGGGCGGC

ABCC2_R5 AAGCCCAACGTGCTGTCCCGGCTGTTCATGTGCTGGGTGTGCCCCGTGCTGGTGGGCGGC

***** ******************************************************

ABCC2_S AACCGGCGCGACGTGGAGGAGCGGGACCTCATACCGCCGCCCAGCGCCAAGTACAAGTCC

ABCC2_R1 AACCGGCGCGACGTGGAGGAGCGGGACCTCATACCGCCGCCCAGCGCCAAGTACAAGTCC

ABCC2_R2 AACCGGCGCGACGTGGAGGAGCGGGACCTCATACCGCCGCCCAGCGCCAAGTACAAGTCC

ABCC2_R3 AACCGGCGCGACGTGGAGGAGCGGGACCTCATACCGCCGCCCAGCGCCAAGTACAAGTCC

ABCC2_R4 AACCGGCGCGACGTGGAGGAGCGGGACCTCATACCGCCGCCCAGCGCCAAGTACAAGTCC

ABCC2_R5 AACCGGCGCGACGTGGAGGAGCGGGACCTCATACCGCCGCCCAGCGCCAAGTACAAGTCC

************************************************************

ABCC2_S GAGTCTCTCGGAGACAAGTTTGAAAGGTACTGGCTAGAAGAGCTGGGTCTGGCGACGCAG

ABCC2_R1 GAGTCTCTCGGAGACAAGTTTGAAAGGTACTGGCTAGAAGAGCTGGGTCTGGCGACGCAG

ABCC2_R2 GAGTCTCTCGGAGACAAGTTTGAAAGGTACTGGCTAGAAGAGCTGGGTCTGGCGACGCAG

ABCC2_R3 GAGTCTCTCGGAGACAAGTTTGAAAGGTACTGGCTAGAAGAGCTGGGTCTGGCGACGCAG

ABCC2_R4 GAGTCTCTCGGAGACAAGTTTGAAAGGTACTGGCTAGAAGAGCTGGGTCTGGCGACGCAG

ABCC2_R5 GAGTCTCTCGGAGACAAGTTTGAAAGGTACTGGCTAGAAGAGCTGGGTCTGGCGACGCAG

************************************************************

**exon 2**

ABCC2_S CGCGGGGTCTCGCCGTCGCTGTGGCGGGCGCTGCGCCGCGCCTTCTGGCTGTCCTACATG

ABCC2_R1 CGCGGGGTGGCACCGTCGCTGTGGCGGGCGCTGCGCCGCGCCTTCTGGCTGTCCTACATG

ABCC2_R2 CGCGGGGTGGCACCGTCGCTGTGGCGGGCGCTGCGCCGCGCCTTCTGGCTGTCCTACATG

ABCC2_R3 CGCGGGGTGGCACCGTCGCTGTGGCGGGCGCTGCGCCGCGCCTTCTGGCTGTCCTACATG

ABCC2_R4 CGCGGGGTGGCACCGTCGCTGTGGCGGGCGCTGCGCCGCGCCTTCTGGCTGTCCTACATG

ABCC2_R5 CGCGGGGTGGCACCGTCGCTGTGGCGGGCGCTGCGCCGCGCCTTCTGGCTGTCCTACATG

******** * ************************************************

ABCC2_S CCCGGCGCGCTGCTGCTGCTCGGGAACGCCATTCCCAGGACCATCCAGCCGCTGCTATTC

ABCC2_R1 CCGGGCGCGCTGCTGTTGCTCGGAAACGCCATTCCTAGGACCATCCAGCCGCTGCTATTC

ABCC2_R2 CCGGGCGCGCTGCTGTTGCTCGGAAACGCCATTCCTAGGACCATCCAGCCGCTGCTATTC

ABCC2_R3 CCGGGCGCGCTGCTGTTGCTCGGAAACGCCATTCCTAGGACCATCCAGCCGCTGCTATTC

ABCC2_R4 CCGGGCGCGCTGCTGTTGCTCGGAAACGCCATTCCTAGGACCATCCAGCCGCTGCTATTC

ABCC2_R5 CCGGGCGCGCTGCTGTTGCTCGGAAACGCCATTCCTAGGACCATCCAGCCGCTGCTATTC

** ************ ******* *********** ************************

ABCC2_S ACGCGACTGCTCTCCTATTGGTCGGCGGACAGCACAATGACCCGACTGGAGGCCGGCTAT

ABCC2_R1 ACGCGGCTGCTCTCCTATTGGTCGGCGGACAGCACCATGACCCGACTGGAGGCCGGCTAT

ABCC2_R2 ACGCGGCTGCTCTCCTATTGGTCGGCGGACAGCACCATGACCCGACTGGAGGCCGGCTAT

ABCC2_R3 ACGCGGCTGCTCTCCTATTGGTCGGCGGACAGCACCATGACCCGACTGGAGGCCGGCTAT

ABCC2_R4 ACGCGGCTGCTCTCCTATTGGTCGGCGGACAGCACCATGACCCGACTGGAGGCCGGCTAT

ABCC2_R5 ACGCGGCTGCTCTCCTATTGGTCGGCGGACAGCACCATGACCCGACTGGAGGCCGGCTAT

***** ***************************** ************************

**exon 3**

ABCC2_S TGGGCCATGGGCATGCTGCTATGCAACTTCCTGGCCATGGTGTGCCACCACCACAACACG

ABCC2_R1 TGGGCCATGGGCATGCTGCTGTGCAACTTCCTGGCCATGGTGTGCCACCACCACAACACG

ABCC2_R2 TGGGCCATGGGCATGCTGCTGTGCAACTTCCTGGCCATGGTGTGCCACCACCACAACACG

ABCC2_R3 TGGGCCATGGGCATGCTGCTGTGCAACTTCCTGGCCATGGTGTGCCACCACCACAACACG

ABCC2_R4 TGGGCCATGGGCATGCTGCTGTGCAACTTCCTGGCCATGGTGTGCCACCACCACAACACG

ABCC2_R5 TGGGCCATGGGCATGCTGCTGTGCAACTTCCTGGCCATGGTGTGCCACCACCACAACACG

******************** ***************************************

ABCC2_S CTATTCGTCGGCCGCTTCGGCATGAAAGTCAGGATTGCCTGTTGCTCGCTGCTCTATCGG

ABCC2_R1 CTGTTCGTCGGCCGCTTCGGCATGAAAGTCAGGATTGCTTGCTGCTCGCTGCTCTATCGG

ABCC2_R2 CTGTTCGTCGGCCGCTTCGGCATGAAAGTCAGGATTGCTTGCTGCTCGCTGCTCTATCGG

ABCC2_R3 CTGTTCGTCGGCCGCTTCGGCATGAAAGTCAGGATTGCTTGCTGCTCGCTGCTCTATCGG

ABCC2_R4 CTGTTCGTCGGCCGCTTCGGCATGAAAGTCAGGATTGCTTGCTGCTCGCTGCTCTATCGG

ABCC2_R5 CTGTTCGTCGGCCGCTTCGGCATGAAAGTCAGGATTGCTTGCTGCTCGCTGCTCTATCGG

** *********************************** ** ******************

ABCC2_S AAGCTGCTCCGCCTTAACCAGAGGTCCCTACAAAGCACGGCAGCAGGCAAGCTGGTGAAC

ABCC2_R1 AAGCTCCTGCGTCTAAACCAGAGATCCCTGCAAAGCACGGCGGCGGGCAAGCTGGTGAAC

ABCC2_R2 AAGCTCCTGCGTCTAAACCAGAGATCCCTGCAAAGCACGGCGGCGGGCAAGCTGGTGAAC

ABCC2_R3 AAGCTCCTGCGTCTAAACCAGAGATCCCTGCAAAGCACGGCGGCGGGCAAGCTGGTGAAC

ABCC2_R4 AAGCTCCTGCGTCTAAACCAGAGATCCCTGCAAAGCACGGCGGCGGGCAAGCTGGTGAAC

ABCC2_R5 AAGCTCCTGCGTCTAAACCAGAGATCCCTGCAAAGCACGGCGGCGGGCAAGCTGGTGAAC

***** ** ** ** ******** ***** *********** ** ***************

ABCC2_S CTGATGTCGAACGACGTGGCGCGCTTCGACTACGCCTTCATGTTCCTGCACTACTTCTGG

ABCC2_R1 CTGATGTCGAACGACGTGGCTCGCTTCGACTACGCCTTCATGTTCCTGCACTATTTCTGG

ABCC2_R2 CTGATGTCGAACGACGTGGCTCGCTTCGACTACGCCTTCATGTTCCTGCACTATTTCTGG

ABCC2_R3 CTGATGTCGAACGACGTGGCTCGCTTCGACTACGCCTTCATGTTCCTGCACTATTTCTGG

ABCC2_R4 CTGATGTCGAACGACGTGGCTCGCTTCGACTACGCCTTCATGTTCCTGCACTATTTCTGG

ABCC2_R5 CTGATGTCGAACGACGTGGCTCGCTTCGACTACGCCTTCATGTTCCTGCACTATTTCTGG

******************** ******************************** ******

**exon 4**

ABCC2_S ATGATCCCGCTGCAGTCGGCTGCGGTGCTGTATTTCATGTTCCGCGCGGCCGGCTGGGCG

ABCC2_R1 ATGATCCCGCTGCAGTCGGCTGCGGTGCTGTACTTCATGTTCCGCGCGGCCGGCTGGGCG

ABCC2_R2 ATGATCCCGCTGCAGTCGGCTGCGGTGCTGTACTTCATGTTCCGCGCGGCCGGCTGGGCG

ABCC2_R3 ATGATCCCGCTGCAGTCGGCTGCGGTGCTGTACTTCATGTTCCGCGCGGCCGGCTGGGCG

ABCC2_R4 ATGATCCCGCTGCAGTCGGCTGCGGTGCTGTACTTCATGTTCCGCGCGGCCGGCTGGGCG

ABCC2_R5 ATGATCCCGCTGCAGTCGGCTGCGGTGCTGTACTTCATGTTCCGCGCGGCCGGCTGGGCG

******************************** ***************************

ABCC2_S CCCATCGTGGGCCTGTTCTCTGTCATGCTGCTCATACTGCCTATACAAGCCGGCCTCACC

ABCC2_R1 CCCATCGTGGGCCTGTTCTCTGTCATGCTGCTCATACTGCCTATACAAGCCGGCCTCACC

ABCC2_R2 CCCATCGTGGGCCTGTTCTCTGTCATGCTGCTCATACTGCCTATACAAGCCGGCCTCACC

ABCC2_R3 CCCATCGTGGGCCTGTTCTCTGTCATGCTGCTCATACTGCCTATACAAGCCGGCCTCACC

ABCC2_R4 CCCATCGTGGGCCTGTTCTCTGTCATGCTGCTCATACTGCCTATACAAGCCGGCCTCACC

ABCC2_R5 CCCATCGTGGGCCTGTTCTCTGTCATGCTGCTCATACTGCCTATACAAGCCGGCCTCACC

************************************************************

**exon 5**

ABCC2_S AAGCTCACAGCTGTCTACAGGAGGGAGACTGCACAAAGGACAGATAAGAGAATTAAACTA

ABCC2_R1 AAGCTCACAGCTGTCTACAGGAGGGAGACTGCACAAAGGACAGATAAGAGAATTAAACTA

ABCC2_R2 AAGCTCACAGCTGTCTACAGGAGGGAGACTGCACAAAGGACAGATAAGAGAATTAAACTA

ABCC2_R3 AAGCTCACAGCTGTCTACAGGAGGGAGACTGCACAAAGGACAGATAAGAGAATTAAACTA

ABCC2_R4 AAGCTCACAGCTGTCTACAGGAGGGAGACTGCACAAAGGACAGATAAGAGAATTAAACTA

ABCC2_R5 AAGCTCACAGCTGTCTACAGGAGGGAGACTGCACAAAGGACAGATAAGAGAATTAAACTA

************************************************************

ABCC2_S ATGGGCGAAATTATCAATGGTATTCAGGTGATAAAGATGTACGCGTGGGAGGTCCCCTTC

ABCC2_R1 ATGAGCGAAATTATCAATG--------GTGATAAAGATGTACGCGTGGGAGGTCCCCTTC

ABCC2_R2 ATGAGCGAAATTATCAATGGTATTCAGGTGATAAAGATGTACGCGTGGGAGGTCCCCTTC

ABCC2_R3 ATGAGCGAAATTATCAATGGTATTCAGGTGATAAAGATGTACGCGTGGGAGGTCCCCTTC

ABCC2_R4 ATGAGCGAAATTATCAATGGTATTCAGGTGATAAAGATGTACGCGTGGGAGGTCCCCTTC

ABCC2_R5 ATGAGCGAAATTATCAATGGTATTCAG---------------------------------

*** ***************

ABCC2_S CAGAAAGTGGTGGGGTCCTCCCGCGCGCACGAGGTGGAGGCGTTGAAGCGGGCGTCCTTC

ABCC2_R1 CAGAAGGTGGTGGGGTCCTCCCGCGCGCACGAGGTGGAGGCGTTGAAGCGGGCGTCCTTC

ABCC2_R2 CAGAAGGTGGTGGGGTCCTCCCGCGCGCACGAGGTGGAGGCGTTGAAGCGGGCGTCCTTC

ABCC2_R3 CAGAAGGTGGTGGGGTCCTCCCGCGCGCACGAGGTGGAGGCGTTGAAGCGGGCGTCCTTC

ABCC2_R4 CAGAAGGTGGTGGGGTCCTCCCGCGCGCACGAGGTGGAGGCGTTGAAGCGGGCGTCCTTC

ABCC2_R5 ------------------------------------------------------------

**exon 6**

ABCC2_S GTGCAGGGCACCTTCGGCGGGTTCATGCTGTTCACGGAGCGCACCTCGCTCTTCCTCACC

ABCC2_R1 GTGCAGGGCACCTTCGGGGGGTTCATGCTGTTCACGGAGCGCACCTCGCTCTTCCTCACC

ABCC2_R2 GTGCAGGGCACCTTCGGGGGGTTCATGCTGTTCACGGAGCGCACCTCGCTCTTCCTCACC

ABCC2_R3 GTGCAGGGCACCTTCGGGGGGTTCATGCTGTTCACGGAGCGCACCTCGCTCTTCCTCACC

ABCC2_R4 GTGCAGGGCACCTTCGGGGGGTTCATGCTGTTCACGGAGCGCACCTCGCTCTTCCTCACC

ABCC2_R5 ------------------------------------------------------------

ABCC2_S GTCATGACGCTCGTGCTGACTGGGAGCATGGCTACTGCTACTACGGTGTATCCAATCCAA

ABCC2_R1 GTCATGACGCTCGTGCTGACTGGGAGCATGGCTACTGCTACTACG---------------

ABCC2_R2 GTCATGACGCTCGTGCTGACTGGGAGCATGGCTACTGCTACTACG---------------

ABCC2_R3 GTCATGACGCTCGTGCTGACTGGGAGCATGGCTACTGCTACTACG---------------

ABCC2_R4 GTCATGACGCTCGTGCTGACTGGGAGCATGGCTACTGCTACTACG---------------

ABCC2_R5 ------------------------------------------------------------

**exon 7**

ABCC2_S CAATACTTTAGTATAATTCAATCCAACTTGGCGCTCATCCTCCCTATCGCCATCGCTCAA

ABCC2_R1 ------------------------------------------------------------

ABCC2_R2 ------------------------------------------------------------

ABCC2_R3 ------------------------------------------------------------

ABCC2_R4 ------------------------------------------------------------

ABCC2_R5 ------------------------------------------------------------

ABCC2_S CTCACTGAGATGTTGGTTTCATTGGAAAGACTTCAGGAGTTTTTGATGTTAGACGAGAGG

ABCC2_R1 ----------------------------------------------------ACGAGAGG

ABCC2_R2 ----------------------------------------------------ACGAGAGG

ABCC2_R3 ----------------------------------------------------ACGAGAGG

ABCC2_R4 ----------------------------------------------------ACGAGAGG

ABCC2_R5 ------------------------------------------------------------

ABCC2_S GAAGACCTGTCGGTGATGCCGGGCGGGCAGGCGGACACCGCGCCCGTGGCCTTCAAGTAC

ABCC2_R1 GAAGACCTGTCGGTGATGCCGGGCGGGCAGGCGGACACCGCGCCCGTGGCCTTCAAGTAC

ABCC2_R2 GAAGACCTGTCGGTGATGCCGGGCGGGCAGGCGGACACCGCGCCCGTGGCCTTCAAGTAC

ABCC2_R3 GAAGACCTGTCGGTGATGCCGGGCGGGCAGGCGGACACCGCGCCCGTGGCCTTCAAGTAC

ABCC2_R4 GAAGACCTGTCGGTGATGCCGGGCGGGCAGGCGGACACCGCGCCCGTGGCCTTCAAGTAC

ABCC2_R5 ------------------------------------------------------------

**exon 8**

ABCC2_S ACGAAGGAGACCACGGCGCCCGCCTACATCGTGTCCAAGAGGTACTCCAAGAAGGAGGAC

ABCC2_R1 ACGAAGGAGACCACGGCGCCCGCCTACATCGTGTCCAAGCGGTACTCCAAAAAGGAGGAC

ABCC2_R2 ACGAAGGAGACCACGGCGCCCGCCTACATCGTGTCCAAGCGGTACTCCAAAAAGGAGGAC

ABCC2_R3 ACGAAGGAGACCACGGCGCCCGCCTACATCGTGTCCAAGCGGTACTCCAAAAAGGAGGAC

ABCC2_R4 ACGAAGGAGACCACGGCGCCCGCCTACATCGTGTCCAAGCGGTACTCCAAAAAGGAGGAC

ABCC2_R5 ------------------------------------------------------------

ABCC2_S GACACCGGCCTGGCTGCGGAGCTGGTGGAGCGCAAATCCACGAGCGAGTTCGCGGTCGAG

ABCC2_R1 GACACTGGCCTAGCTGCGGAGCTAGTGGAGCGCAAGGCTACGAGCGAGTTCGCGGTCGAG

ABCC2_R2 GACACTGGCCTAGCTGCGGAGCTAGTGGAGCGCAAGGCTACGAGCGAGTTCGCGGTCGAG

ABCC2_R3 GACACTGGCCTAGCTGCGGAGCTAGTGGAGCGCAAGGCTACGAGCGAGTTCGCGGTCGAG

ABCC2_R4 GACACTGGCCTAGCTGCGGAGCTAGTGGAGCGCAAGGCTACGAGCGAGTTCGCGGTCGAG

ABCC2_R5 ------------------------------------------------------------

ABCC2_S CTGAACGACGTGAGCGCGTCGTGGGGGGGTGAGGGGGACAAGGACCAGCACACGCTGCGC

ABCC2_R1 CTGAACGACGTGAGTGCTTCGTGGGGGGGTGAGGGGGACAAGGACGAGCATACTCTGCGG

ABCC2_R2 CTGAACGACGTGAGTGCTTCGTGGGGGGGTGAGGGGGACAAGGACGAGCATACTCTGCGG

ABCC2_R3 CTGAACGACGTGAGTGCTTCGTGGGGGGGTGAGGGGGACAAGGACGAGCATACTCTGCGG

ABCC2_R4 CTGAACGACGTGAGTGCTTCGTGGGGGGGTGAGGGGGACAAGGACGAGCATACTCTGCGG

ABCC2_R5 ------------------------------------------------------------

**exon 9**

ABCC2_S GGGGTGTCCATGCGCGTGCGCCGCGGCAAGCTGGCCGCCATCATCGGACCCGTCGGCTCA

ABCC2_R1 GGGGTGTCGATGCGCGTGCGCCGCGGCAAGCTGGCCGCTATCATCGGACCCGTCGGCTCG

ABCC2_R2 GGGGTGTCGATGCGCGTGCGCCGCGGCAAGCTGGCCGCTATCATCGGACCCGTCGGCTCG

ABCC2_R3 GGGGTGTCGATGCGCGTGCGCCGCGGCAAGCTGGCCGCTATCATCGGACCCGTCGGCTCG

ABCC2_R4 GGGGTGTCGATGCGCGTGCGCCGCGGCAAGCTGGCCGCTATCATCGGACCCGTCGGCTCG

ABCC2_R5 ------------------------------------------------------------

**exon 10**

ABCC2_S GGGAAGTCATCGTTGCTCCAAGTGTTGCTGAAAGAGTTGCCGGTGTCGTCGGGCAGCGTG

ABCC2_R1 GGGAAGTCGTCGCTACTTCAAGTGTTGCTGAAAGAGCTTCCGGTGTCTTCCGGCACCGTG

ABCC2_R2 GGGAAGTCGTCGCTACTTCAAGTGTTGCTGAAAGAGCTTCCGGTGTCTTCCGGCACCGTG

ABCC2_R3 GGGAAGTCGTCGCTACTTCAAGTGTTGCTGAAAGAGCTTCCGGTGTCTTCCGGCACCGTG

ABCC2_R4 GGGAAGTCGTCGCTACTTCAAGTGTTGCTGAAAGAGCTTCCGGTGTCTTCCGGCACCGTG

ABCC2_R5 ------------------------------------------------------------

ABCC2_S GGTGTGCACGGCCAGATCTCGTACGCGTGCCAGGAGTCCTGGCTCTTCTCCGCCACCATC

ABCC2_R1 GGTGTGCACGGCCAAATCTCGTACGCATGCCAGGAGTCCTGGCTCTTCTCCGCCACGGTC

ABCC2_R2 GGTGTGCACGGCCAAATCTCGTACGCATGCCAGGAGTCCTGGCTCTTCTCCGCCACGGTC

ABCC2_R3 GGTGTGCACGGCCAAATCTCGTACGCATGCCAGGAGTCCTGGCTCTTCTCCGCCACGGTC

ABCC2_R4 GGTGTGCACGGCCAAATCTCGTACGCATGCCAGGAGTCCTGGCTCTTCTCCGCCACGGTC

ABCC2_R5 ------------------------------------------------------------

ABCC2_S CGGGATAACATCCTCTTCGGACTGCCGTACGACTCCAAGAAATATAAGAAGGTGTGCGAC

ABCC2_R1 CGGGATAACATACTGTTTGGGCTGCCGTATGACTCCAAGAAATATAAGAAGGTGTGCGAC

ABCC2_R2 CGGGATAACATACTGTTTGGGCTGCCGTATGACTCCAAGAAATATAAGAAGGTGTGCGAC

ABCC2_R3 CGGGATAACATACTGTTTGGGCTGCCGTATGACTCCAAGAAATATAAGAAGGTGTGCGAC

ABCC2_R4 CGGGATAACATACTGTTTGGGCTGCCGTATGACTCCAAGAAATATAAGAAGGTGTGCGAC

ABCC2_R5 ---------------------------------------------------GTGTGCGAC

*********

ABCC2_S GCGTGCTGCCTGCAGCCCGACTTCAAGCAGTTCCCGTACGGAGACCTGTCGCTGGTGGGC

ABCC2_R1 GCGTGCTGCCTGCAGCCTGACTTCAAGCAGTTCCCGTACGGAGACCTGTCGCTGGTGGGC

ABCC2_R2 GCGTGCTGCCTGCAGCCTGACTTCAAGCAGTTCCCGTACGGAGACCTGTCGCTGGTGGGC

ABCC2_R3 GCGTGCTGCCTGCAGCCTGACTTCAAGCAGTTCCCGTACGGAGACCTGTCGCTGGTGGGC

ABCC2_R4 GCGTGCTGCCTGCAGCCTGACTTCAAGCAGTTCCCGTACGGAGACCTGTCGCTGGTGGGC

ABCC2_R5 GCGTGCTGCCTGCAGCCTGACTTCAAGCAGTTCCCGTACGGAGACCTGTCGCTGGTGGGC

***************** ******************************************

**exon 11**

ABCC2_S GAGCGCGGCGTGTCGCTGTCCGGGGGGCAACGCGCCCGGATCAACCTCGCGCGCGCCGTG

ABCC2_R1 GAGCGCGGCGTGTCGCTGTCCGGGGGGCAGCGCGCCCGGATCAACCTGGCCCGCGCCGTG

ABCC2_R2 GAGCGCGGCGTGTCGCTGTCCGGGGGGCAGCGCGCCCGGATCAACCTGGCCCGCGCCGTG

ABCC2_R3 GAGCGCGGCGTGTCGCTGTCCGGGGGGCAGCGCGCCCGGATCAACCTGGCCCGCGCCGTG

ABCC2_R4 GAGCGCGGCGTGTCGCTGTCCGGGGGGCAGCGCGCCCGGATCAACCTGGCCCGCGCCGTG

ABCC2_R5 GAGCGCGGCGTGTCGCTGTCCGGGGGGCAGCGCGCCCGGATCAACCTGGCCCGCGCCGTG

***************************** ***************** ** *********

ABCC2_S TACCGGGAC---------------------------------------------------

ABCC2_R1 TACCGGGAC---------------------------------------------------

ABCC2_R2 TACCGGGAC---------------------------------------------------

ABCC2_R3 TACCGGGAC---------------------------------------------------

ABCC2_R4 TACCGGGACGTATGTATACACACAAACACGGAGACACACAGACATCGCACTACCTAGGAA

ABCC2_R5 TACCGGGAC---------------------------------------------------

*********

ABCC2_S ------------------------------------------------------------

ABCC2_R1 ------------------------------------------------------------

ABCC2_R2 ------------------------------------------------------------

ABCC2_R3 ------------------------------------------------------------

ABCC2_R4 CAAAAAGTACCTAATAGAAAAAAAGCTCTAGCTATTATTCTAGAGATGCGACACCTTAAA

ABCC2_R5 ------------------------------------------------------------

ABCC2_S ------------------------------------------------------------

ABCC2_R1 ------------------------------------------------------------

ABCC2_R2 ------------------------------------------------------------

ABCC2_R3 ------------------------------------------------------------

ABCC2_R4 AAATAAACCACTGGAAACTGAATATCTTTGATCTAAGTAAAACTTACTGAAACTATTCCA

ABCC2_R5 ------------------------------------------------------------

ABCC2_S ------------------------------------------------------------

ABCC2_R1 ------------------------------------------------------------

ABCC2_R2 ------------------------------------------------------------

ABCC2_R3 ------------------------------------------------------------

ABCC2_R4 CCCTAAGTTGCTCAATATCCGTGTTATTTTTCTTTATAAAAGTTGAACAGAAGAAAAATA

ABCC2_R5 ------------------------------------------------------------

ABCC2_S ------------------------------------------------------------

ABCC2_R1 ------------------------------------------------------------

ABCC2_R2 ------------------------------------------------------------

ABCC2_R3 ------------------------------------------------------------

ABCC2_R4 CGCGAGGAGAATGGGCTTAGAAAACTTTTAAGTTAGTTATTAAACAATGGCTTAGCAAGT

ABCC2_R5 ------------------------------------------------------------

ABCC2_S ----------------------GCCGACATCTACATATTCGACGACCCGCTGTCGGCGGT

ABCC2_R1 ----------------------GCTGACATCTACATATTCGATGACCCCCTATCGGCGGT

ABCC2_R2 ----------------------GCTGACATCTACATATTCGATGACCCCCTATCGGCGGT

ABCC2_R3 ----------------------GCTGACATCTACATATTCGATGACCCCCTATCGGCGGT

ABCC2_R4 TAGCAACCTATCTACATTTCAGGCTGACATCTACATATTCGATGACCCCCTATCGGCGGT

ABCC2_R5 ----------------------GCTGACATCTACATATTCGATGACCCCCTATCGGCGGT

** ***************** ***** ** ********

ABCC2_S GGACGCGAATGTGGGTCGGCAGCTGTTCGAGGGCTGCATCAACGGCTACCTGCGCGGCCG

ABCC2_R1 GGACGCGAATGTAGGTCGGCAGCTGTTCGAGGGCTGCATCAACGGCTACCTGCGCGGCCG

ABCC2_R2 GGACGCGAATGTAGGTCGGCAGCTGTTCGAGGGCTGCATCAACGGCTACCTGCGCGGCCG

ABCC2_R3 GGACGCGAATGTAGGTCGGCAGCTGTTCGAGGGCTGCATCAACGGCTACCTGCGCGGCCG

ABCC2_R4 GGACGCGAATGTAGGTCGGCAGCTGTTCGAGGGCTGCATCAACGGCTACCTGCGCGGCCG

ABCC2_R5 GGACGCGAATGTAGGTCGGCAGCTGTTCGAGGGCTGCATCAACGGCTACCTGCGCGGCCG

************ ***********************************************

**exon 12**

ABCC2_S CACGCGCGTGCTCGTCACGCATCAGATACACTTCCTCAAGGCCGCAGACTACATAGTCAT

ABCC2_R1 CACGCGCGTGCTCGTCACGCATCAGATACACTTCCTCAAGGCCGCAGACTACATAGTCAT

ABCC2_R2 CACGCGCGTGCTCGTCACGCATCAGATACACTTCCTCAAGGCCGCAGACTACATAGTCAT

ABCC2_R3 CACGCGCGTGCTCGTCACGCATCAGATACACTTCCTCAAGGCCGCAGACTACATAGTCAT

ABCC2_R4 CACGCGCGTGCTCGTCACGCATCAGATACACTTCCTCAAGGCCGCAGACTACATAGTCAT

ABCC2_R5 CACGCGCGTGCTCGTCACGCATCAGATACACTTCCTCAAGGCCGCAGACTACATAGTCAT

************************************************************

ABCC2_S ACTCAACGAG--------------------------------------------------

ABCC2_R1 ACTCAACGAG--------------------------------------------------

ABCC2_R2 ACTCAACGAG--------------------------------------------------

ABCC2_R3 ACTCAACGAGGTAATTGCACACACAATCTTACACGTGTAATGTTAAATGAAGCGTTTAAT

ABCC2_R4 ACTCAACGAG--------------------------------------------------

ABCC2_R5 ACTCAACGAG--------------------------------------------------

**********

ABCC2_S -------------------------GGTGCCATTGAAAATATGGGCACGTACGATGATTT

ABCC2_R1 -------------------------GGTGCCATTGAAAATATGGGCACGTACGATGATTT

ABCC2_R2 -------------------------GGTGCCATTGAAAATATGGGCACGTACGATGATTT

ABCC2_R3 AACATGATTGTTTACCTTGGACCAGGGTGCCATTGAAAATATGGGCACGTACGATGATTT

ABCC2_R4 -------------------------GGTGCCATTGAAAATATGGGCACGTACGATGATTT

ABCC2_R5 -------------------------GGTGCCATTGAAAATATGGGCACGTACGATGATTT

***********************************

**exon 13**

ABCC2_S AACGAAGCTGGAGAATTCGCTGCTGTTGCCGAAGCAACAGGAGGGTTCCGGCGATGACAG

ABCC2_R1 AACGAAGCTGGAGAATTCGCTGCTGCTGCCCAAGCAACAGGAGGGTTCTGGCGATGATAG

ABCC2_R2 AACGAAGCTGGAGAATTCGCTGCTGCTGCCCAAGCAACAGGAGGGTTCTGGCGATGATAG

ABCC2_R3 AACGAAGCTGGAGAATTCGCTGCTGCTGCCCAAGCAACAGGAGGGTTCTGGCGATGATAG

ABCC2_R4 AACGAAGCTGGAGAATTCGCTGCTGCTGCCCAAGCAACAGGAGGGTTCTGGCGATGATAG

ABCC2_R5 AACGAAGCTGGAGAATTCGCTGCTGCTGCCCAAGCAACAGGAGGGTTCTGGCGATGATAG

************************* **** ***************** ******** **

ABCC2_S TAAGGATGAACTAGCTATTCCCAATGCAGCAAAGAAGCCAATTATGGAACGGGGGGTATC

ABCC2_R1 TAAGGGTGAACTAGCTATTCCCAATGCAGCGAAGAAGCCAATAGTGGAACGGGGGATATC

ABCC2_R2 TAAGGGTGAACTAGCTATTCCCAATGCAGCGAAGAAGCCAATAGTGGAACGGGGGATATC

ABCC2_R3 TAAGGGTGAACTAGCTATTCCCAATGCAGCGAAGAAGCCAATAGTGGAACGGGGGATATC

ABCC2_R4 TAAGGGTGAACTAGCTATTCCCAATGCAGCGAAGAAGCCAATAGTGGAACGGGGGATATC

ABCC2_R5 TAAGGGTGAACTAGCTATTCCCAATGCAGCGAAGAAGCCAATAGTGGAACGGGGGATATC

***** ************************ *********** *********** ****

ABCC2_S AGTGATCTCAGTGAAGAGCGAAGACAACGGTGAGGCTCGCAAGGAACAAGTGCAGGCGGC

ABCC2_R1 AGTGATCTCAGTGAAGAGCGAAGACAACGGTGAGGCTCGCAAGGAGCAAATCCAGGCGGC

ABCC2_R2 AGTGATCTCAGTGAAGAGCGAAGACAACGGTGAGGCTCGCAAGGAGCAAATCCAGGCGGC

ABCC2_R3 AGTGATCTCAGTGAAGAGCGAAGACAACGGTGAGGCTCGCAAGGAGCAAATCCAGGCGGC

ABCC2_R4 AGTGATCTCAGTGAAGAGCGAAGACAACGGTGAGGCTCGCAAGGAGCAAATCCAGGCGGC

ABCC2_R5 AGTGATCTCAGTGAAGAGCGAAGACAACGGTGAGGCTCGCAAGGAGCAAATCCAGGCGGC

********************************************* *** * ********

**exon 14**

ABCC2_S GGAGGAGCGCGCCTCCGGGAACCTCAAGTGGGAGGTGTTCGCGAGGTACCTGGTCTCCGT

ABCC2_R1 GGAGGAGCGCGCCTCCGGGAACCTCAAGTGGGAGGTGTTCGCGAGGTACCTGGTCTCCGT

ABCC2_R2 GGAGGAGCGCGCCTCCGGGAACCTCAAGTGGGAGGTGTTCGCGAGGTACCTGGTCTCCGT

ABCC2_R3 GGAGGAGCGCGCCTCCGGGAACCTCAAGTGGGAGGTGTTCGCGAGGTACCTGGTCTCCGT

ABCC2_R4 GGAGGAGCGCGCCTCCGGGAACCTCAAGTGGGAGGTGTTCGCGAGGTACCTGGTCTCCGT

ABCC2_R5 GGAGGAGCGCGCCTCCGGGAACCTCAAGTGGGAGGTGTTCGCGAGGTACCTGGTCTCCGT

************************************************************

ABCC2_S GGACTCCTGGGCCATCGTGGCGCTCACGCTCACGGCGATGCTCATCACCCAAGGCGCGGC

ABCC2_R1 GGACTCCTGGGCCATCGTGGCGCTCACGCTCACCGCGATGCTCATCACCCAGGGGGCGGC

ABCC2_R2 GGACTCCTGGGCCATCGTGGCGCTCACGCTCACCGCGATGCTCATCACCCAGGGGGCGGC

ABCC2_R3 GGACTCCTGGGCCATCGTGGCGCTCACGCTCACCGCGATGCTCATCACCCAGGGGGCGGC

ABCC2_R4 GGACTCCTGGGCCATCGTGGCGCTCACGCTCACCGCGATGCTCATCACCCAGGGGGCGGC

ABCC2_R5 GGACTCCTGGGCCATCGTGGCGCTCACGCTCACCGCGATGCTCATCACCCAGGGGGCGGC

********************************* ***************** ** *****

**exon 15**

ABCC2_S CTCCTCCACCGATTATTGGCTGAGTTTCTGGACAAATCAAGTTGATGGATACATACAAGA

ABCC2_R1 GTCGTCCACCGACTACTGGCTTAGCTTCTGGACAAATCAAGTTGATGGATACATACAAGA

ABCC2_R2 GTCGTCCACCGACTACTGGCTTAGCTTCTG-----------------------------A

ABCC2_R3 GTCGTCCACCGACTACTGGCTTAGCTTCTGGACAAATCAAGTTGATGGATACATACAAGA

ABCC2_R4 GTCGTCCACCGACTACTGGCTTAGCTTCTGGACAAATCAAGTTGATGGATACATACAAGA

ABCC2_R5 GTCGTCCACCGACTACTGGCTTAGCTTCTGGACAAATCAAGTTGATGGATACATACAAGA

** ******** ** ***** ** ****** *

ABCC2_S CCTGCCAGATGGGGAGGAGCCAG-------------------------------------

ABCC2_R1 CCTGCCAGATGGGGAGGAACCAG-------------------------------------

ABCC2_R2 CCTGCCAGATGGGGAGGAACCAG-------------------------------------

ABCC2_R3 CCTGCCAGATGGGGAGGAACCAG-------------------------------------

ABCC2_R4 CCTGCCAGATGGGGAGGAACCAGGTGAGTTCTTACTTGTTATACCTGAAAGAGCCAAAAG

ABCC2_R5 CCTGCCAGATGGGGAGGAACCAG-------------------------------------

****************** ****

ABCC2_S ------------------------------------------------------------

ABCC2_R1 ------------------------------------------------------------

ABCC2_R2 ------------------------------------------------------------

ABCC2_R3 ------------------------------------------------------------

ABCC2_R4 GGTAGTGATACCAATACCAGAATATCATCGCCTTGGAGATCAATTACTCTGACCCACAAA

ABCC2_R5 ------------------------------------------------------------

ABCC2_S ------------------------------------------------------------

ABCC2_R1 ------------------------------------------------------------

ABCC2_R2 ------------------------------------------------------------

ABCC2_R3 ------------------------------------------------------------

ABCC2_R4 TTTTGTGAAAAATAAGTTAGGTATACCAAACGATTCAAAATCGATTAAGTGTACAGTGGA

ABCC2_R5 ------------------------------------------------------------

ABCC2_S ------------------------------------------------------------

ABCC2_R1 ------------------------------------------------------------

ABCC2_R2 ------------------------------------------------------------

ABCC2_R3 ------------------------------------------------------------

ABCC2_R4 CAATCAATCTAAGCCTCAAAAAAAATTGTGGGTTGGACTAATTGACCTCCAAGGCGAGGA

ABCC2_R5 ------------------------------------------------------------

ABCC2_S ---------------------------------------ACCCAAGTCTCGGCACACAAA

ABCC2_R1 ---------------------------------------ATCCAAGTCTCGGCACGCAAA

ABCC2_R2 ---------------------------------------ATCCAAGTCTCGGCACGCAAA

ABCC2_R3 ---------------------------------------ATCCAAGTCTCGGCACGCAAA

ABCC2_R4 CATGAAAGTAAAATTTTGTACAATATGTTGTATTCCCAGATCCAAGTCTCGGCACGCAAA

ABCC2_R5 ---------------------------------------ATCCAAGTCTCGGCACGCAAA

* ************** ****

ABCC2_S CGGGCATCCTCCAGACCGGCCAGTACGTGTACATCTACGGCGCGCTGGTGCTGACCATCA

ABCC2_R1 CAGGCATCCTGGAGACGGGCCAGTACGTGTACATCTACGGCGCGCTGGTGCTGACCATAA

ABCC2_R2 CAGGCATCCTGGAGACGGGCCAGTACGTGTACATCTACGGCGCGCTGGTGCTGACCATAA

ABCC2_R3 CAGGCATCCTGGAGACGGGCCAGTACGTGTACATCTACGGCGCGCTGGTGCTGACCATAA

ABCC2_R4 CAGGCATCCTGGAGACGGGCCAGTACGTGTACATCTACGGCGCGCTGGTGCTGACCATAA

ABCC2_R5 CAGGCATCCTGGAGACGGGCCAGTACGTGTACATCTACGGCGCGCTGGTGCTGACCATAA

* ******** **** ***************************************** *

**exon 16**

ABCC2_S TCGTGATGTCGTTCATGCGTCTGTTCGGCTTCGTGACCATGACCATGCGCGCCGCCGCCA

ABCC2_R1 TCGTGATGTCCTTCATGCGGCTGTTCGGCTTCGTGACCATGACCATGCGCGCCGCCGCCA

ABCC2_R2 TCGTGATGTCCTTCATGCGGCTGTTCGGCTTCGTGACCATGACCATGCGCGCCACCGCCA

ABCC2_R3 TCGTGATGTCCTTCATGCGGCTGTTCGGCTTCGTGACCATGACCATGCGCGCCGCCGCCA

ABCC2_R4 TCGTGATGTCCTTCATGCGGCTGTTCGGCTTCGTGACCATGACCATGCGCGCCGCCGCCA

ABCC2_R5 TCGTGATGTCCTTCATGCGGCTGTTCGGCTTCGTGACCATGACCATGCGCGCCGCCGCCA

********** ******** ********************************* ******

ABCC2_S ACATCCACGACCTCATGTTCCGCAACCTCATACGCGCCACCATGCGCTTCTTTGACACCA

ABCC2_R1 ACATCCACGACCTCATGTTCCGCAACCTCATCCGCGCCACCATGCGCTTCTTTGACACCA

ABCC2_R2 ACATCCACGACCTCATGTTCCGCAACCTCATCCGCGCCACCATGCGCTTCTTTGACACCA

ABCC2_R3 ACATCCACGACCTCATGTTCCGCAACCTCATCCGCGCCACCATGCGCTTCTTTGACACCA

ABCC2_R4 ACATCCACGACCTCATGTTCCGCAACCTCATCCGCGCCACCATGCGCTTCTTTGACACCA

ABCC2_R5 ACATCCACGACCTCATGTTCCGCAACCTCATCCGCGCCACCATGCGCTTCTTTGACACCA

******************************* ****************************

ABCC2_S ATCCTTCAG---------------------------------------------------

ABCC2_R1 ATCCTTCGG---------------------------------------------------

ABCC2_R2 ATCCTTCGG---------------------------------------------------

ABCC2_R3 ATCCTTCGG---------------------------------------------------

ABCC2_R4 ATCCTTCGGGTACTGTATTGGTTTATTCTGTCGTTCTGATGACGTACTTATCTAAATTCA

ABCC2_R5 ATCCTTCGG---------------------------------------------------

******* *

ABCC2_S ------------------------------------------------------------

ABCC2_R1 ------------------------------------------------------------

ABCC2_R2 ------------------------------------------------------------

ABCC2_R3 ------------------------------------------------------------

ABCC2_R4 TGCTGGGAATGCAGCGCACTGGACACTAAGATAAAAGACCTGGAAGACCTAACTAACTGA

ABCC2_R5 ------------------------------------------------------------

ABCC2_S ------------------------------------------------------------

ABCC2_R1 ------------------------------------------------------------

ABCC2_R2 ------------------------------------------------------------

ABCC2_R3 ------------------------------------------------------------

ABCC2_R4 CGAATCCTTTCACAACGATGAAACTAACTCCGAATGTATTTGCGTACACAAATACCCTCA

ABCC2_R5 ------------------------------------------------------------

ABCC2_S ------------------------------------------------------------

ABCC2_R1 ------------------------------------------------------------

ABCC2_R2 ------------------------------------------------------------

ABCC2_R3 ------------------------------------------------------------

ABCC2_R4 AAAGGTACAGGTTATAATAAGTTTCCATCAAAATGACAAAGATGATTTAAATTCACATAG

ABCC2_R5 ------------------------------------------------------------

ABCC2_S ------------------------------------------------------------

ABCC2_R1 ------------------------------------------------------------

ABCC2_R2 ------------------------------------------------------------

ABCC2_R3 ------------------------------------------------------------

ABCC2_R4 TCATCAGACCTAACCACCCGGAAGAACCGTTATCATCATCTCACACACAACCAATTCACA

ABCC2_R5 ------------------------------------------------------------

ABCC2_S ------------------------------------------------------GTCGCG

ABCC2_R1 ------------------------------------------------------GTCGCG

ABCC2_R2 ------------------------------------------------------GTCGCG

ABCC2_R3 ------------------------------------------------------GTCGCG

ABCC2_R4 GATCTTATACAGTATGCAGCTCTTATTCACTTGTTTACAACACTCCATCTATAGGTCGCG

ABCC2_R5 ------------------------------------------------------GTCGCG

******

ABCC2_S TGCTAAACCGGTTCTCCAAAGACATGGGCGGCATGGACGAGCTGCTGCCCAGGTCCATCC

ABCC2_R1 TGCTAAACCGGTTCTCCAAAGACATGGGCGGTATGGACGAGCTGCTGCCGAGGTCCATCC

ABCC2_R2 TGCTAAACCGGTTCTCCAAAGACATGGGCGGTATGGACGAGCTGCTGCCGAGGTCCATCC

ABCC2_R3 TGCTAAACCGGTTCTCCAAAGACATGGGCGGTATGGACGAGCTGCTGCCGAGGTCCATCC

ABCC2_R4 TGCTAAACCGGTTCTCCAAAGACATGGGCGGTATGGACGAGCTGCTGCCGAGGTCCATCC

ABCC2_R5 TGCTAAACCGGTTCTCCAAAGACATGGGCGGTATGGACGAGCTGCTGCCGAGGTCCATCC

******************************* ***************** **********

**exon 17**

ABCC2_S TGCAGGCCTTCCAGATGTACCTGTCCATGGCGAGCGTGCTCACGCTGAACGCCGTCTCCC

ABCC2_R1 TGCAGGCCTTCCAGATGTACCTGTCCATGGCGAGCGTGCTCACGCTGAACGCCGTCTCCC

ABCC2_R2 TGCAGGCCTTCCAGATGTACCTGTCCATGGCGAGCGTGCTCACGCTGAACGCCGTCTCCC

ABCC2_R3 TGCAGGCCTTCCAGATGTACCTGTCCATGGCGAGCGTGCTCACGCTGAACGCCGTCTCCC

ABCC2_R4 TGCAGGCCTTCCAGATGTACCTGTCCATGGCGAGCGTGCTCACGCTGAACGCCGTCTCCC

ABCC2_R5 TGCAGGCCTTCCAGATGTACCTGTCCATGGCGAGCGTGCTCACGCTGAACGCCGTCTCCC

************************************************************

ABCC2_S TGCCCTGGACCCTCATACCCACGGTGCTGCTGCTGGGGCTCTTCATCAGGTACCTCAAGT

ABCC2_R1 TGCCCTGGACCCTCATACCCACGGTGCTGCTGCTGGGCCTCTTCATCAGGTACCTCAAGT

ABCC2_R2 TGCCCTGGACCCTCATACCCACGGTGCTGCTGCTGGGCCTCTTCATCAGGTACCTCAAGT

ABCC2_R3 TGCCCTGGACCCTCATACCCACGGTGCTGCTGCTGGGCCTCTTCATCAGGTACCTCAAGT

ABCC2_R4 TGCCCTGGACCCTCATACCCACGGTGCTGCTGCTGGGCCTCTTCATCAGGTACCTCAAGT

ABCC2_R5 TGCCCTGGACCCTCATACCCACGGTGCTGCTGCTGGGCCTCTTCATCAGGTACCTCAAGT

************************************* **********************

ABCC2_S GGTACCTGAACGCTGCGCAGTCTGTGAAGAGGCTGGAGGGTACAACTAAGAGTCCAGTGT

ABCC2_R1 GGTACCTGAACGCTGCGCAGTCTGTGAAGAGGCTGGAGGGTACAACTAAGAGTCCAGTGT

ABCC2_R2 GGTACCTGAACGCTGCGCAGTCTGTGAAGAGGCTGGAGGGTACAACTAAGAGTCCAGTGT

ABCC2_R3 GGTACCTGAACGCTGCGCAGTCTGTGAAGAGGCTGGAGGGTACAACTAAGAGTCCAGTGT

ABCC2_R4 GGTACCTGAACGCTGCGCAGTCTGTGAAGAGGCTGGAGGGTACAACTAAGAGTCCAGTGT

ABCC2_R5 GGTACCTGAACGCTGCGCAGTCTGTGAAGAGGCTGGAGGGTACAACTAAGAGTCCAGTGT

************************************************************

**exon 18**

ABCC2_S TTGGAATGATCGGCTCTACTCTATCGGGAATGTCGACAATCAGAAGTTCAGACTCACAGG

ABCC2_R1 TTGGAATGATCGGCTCTACTCTATCGGGAATGTCGACAATCAGAAGTTCAGACTCACAGG

ABCC2_R2 TTGGAATGATCGGCTCTACTCTATCGGGAATGTCGACAATCAGAAGTTCAGACTCACAGG

ABCC2_R3 TTGGAATGATCGGCTCTACTCTATCGGGAATGTCGACAATCAGAAGTTCAGACTCACAGG

ABCC2_R4 TTGGAATGATCGGCTCTACTCTATCGGGAATGTCGACAATCAGAAGTTCAGACTCACAGG

ABCC2_R5 TTGGAATGATCGGCTCTACTCTATCGGGAATGTCGACAATCAGAAGTTCAGACTCACAGG

************************************************************

ABCC2_S ACAGACTAATCAAAAGCTTCGACGACTGCCAGAATCTCCATACTTCTGCATTCCACACTT

ABCC2_R1 ACAGACTAATCAAAAACTTCGACGACTGCCAGAATCTCCATACTTCTGCCTTCCACACTT

ABCC2_R2 ACAGACTAATCAAAAACTTCGACGACTGCCAGAATCTCCATACTTCTGCCTTCCACACTT

ABCC2_R3 ACAGACTAATCAAAAACTTCGACGACTGCCAGAATCTCCATACTTCTGCCTTCCACACTT

ABCC2_R4 ACAGACTAATCAAAAACTTCGACGACTGCCAGAATCTCCATACTTCTGCCTTCCACACTT

ABCC2_R5 ACAGACTAATCAAAAACTTCGACGACTGCCAGAATCTCCATACTTCTGCCTTCCACACTT

*************** ********************************* **********

**exon 19**

ABCC2_S ACATCGGGGGAGCCACGGCCTTCGGATTCTATTTGGATATGATATGCCTCGTCTACCTCG

ABCC2_R1 ACATCGGGGGAGCCACGGCCTTCGGATTCTATTTGGATATGATATGCCTCGTCTACCTCG

ABCC2_R2 ACATCGGGGGAGCCACGGCCTTCGGATTCTATTTGGATATGATATGCCTCGTCTACCTCG

ABCC2_R3 ACATCGGGGGAGCCACGGCCTTCGGATTCTATTTGGATATGATATGCCTCGTCTACCTCG

ABCC2_R4 ACATCGGGGGAGCCACGGCCTTCGGATTCTATTTGGATATGATATGCCTCGTCTACCTCG

ABCC2_R5 ACATCGGGGGAGCCACGGCCTTCGGATTCTATTTGGATATGATATGCCTCGTCTACCTCG

************************************************************

ABCC2_S CATCTATATTATCAATTTTCATCCTTATCGACTTCGCGGACGTGATCCCGGTGGGCAGCG

ABCC2_R1 CATCTATATTATCAATTTTCATCCTTATCGACTTCGCGGACGTGATCCCGGTGGGCAGCG

ABCC2_R2 CATCTATATTATCAATTTTCATCCTTATCGACTTCGCGGACGTGATCCCGGTGGGCAGCG

ABCC2_R3 CATCTATATTATCAATTTTCATCCTTATCGACTTCGCGGACGTGATCCCGGTGGGCAGCG

ABCC2_R4 CATCTATATTATCAATTTTCATCCTTATCGACTTCGCGGACGTGATCCCGGTGGGCAGCG

ABCC2_R5 CATCTATATTATCAATTTTCATCCTTATCGACTTCGCGGACGTGATCCCGGTGGGCAGCG

************************************************************

ABCC2_S TGGGGCTGGCGGTGAGCCAGTCCATGGTGCTGACGGTGCTGCTGCAGCTCGCGGCGCGGT

ABCC2_R1 TGGGGCTGGCGGTGAGCCAGTCCATGGTGCTGACGGTGCTGCTGCAGCTGGCCGCGCGGT

ABCC2_R2 TGGGGCTGGCGGTGAGCCAGTCCATGGTGCTGACGGTGCTGCTGCAGCTGGCCGCGCGGT

ABCC2_R3 TGGGGCTGGCGGTGAGCCAGTCCATGGTGCTGACGGTGCTGCTGCAGCTGGCCGCGCGGT

ABCC2_R4 TGGGGCTGGCGGTGAGCCAGTCCATGGTGCTGACGGTGCTGCTGCAGCTGGCCGCGCGGT

ABCC2_R5 TGGGGCTGGCGGTGAGCCAGTCCATGGTGCTGACGGTGCTGCTGCAGCTGGCCGCGCGGT

************************************************* ** *******

**exon 20**

ABCC2_S TCACCAGCGACTTCCTGGCGCAGATGACCGCCGTGGAGCGCGTGCTCGAGTACACTAAAC

ABCC2_R1 TCACCAGCGACTTCCTGGCGCAGATGACCGCCGTGGAGCGCGTGCTCGAGTACACTAAAC

ABCC2_R2 TCACCAGCGACTTCCTGGCGCAGATGACCGCCGTGGAGCGCGTGCTCGAGTACACTAAAC

ABCC2_R3 TCACCAGCGACTTCCTGGCGCAGATGACCGCCGTGGAGCGCGTGCTCGAGTACACTAAAC

ABCC2_R4 TCACCAGCGACTTCCTGGCGCAGATGACCGCCGTGGAGCGCGTGCTCGAGTACACTAAAC

ABCC2_R5 TCACCAGCGACTTCCTGGCGCAGATGACCGCCGTGGAGCGCGTGCTCGAGTACACTAAAC

************************************************************

ABCC2_S TGCCGCACGAGGAGAATATTAATGATGGCCCTACGCAGCCGCCCAAGACATGGCCTGCTG

ABCC2_R1 TGCCGCACGAGGAGAATATTAATGATGGACCTACGCATCCGCCCAAGACATGGCCTGCTG

ABCC2_R2 TGCCGCACGAGGAGAATATTAATGATGGACCTACGCATCCGCCCAAGACATGGCCTGCTG

ABCC2_R3 TGCCGCACGAGGAGAATATTAATGATGGACCTACGCATCCGCCCAAGACATGGCCTGCTG

ABCC2_R4 TGCCGCACGAGGAGAATATTAATGATGGACCTACGCATCCGCCCAAGACATGGCCTGCTG

ABCC2_R5 TGCCGCACGAGGAGAATATTAATGATGGACCTACGCATCCGCCCAAGACATGGCCTGCTG

**************************** ******** **********************

**exon 21**

ABCC2_S AAGGTAACATCAAATTTGAAAACGTTTTCCTGACTTACTCGTTGGAGGACCCACCCGTGC

ABCC2_R1 AAGGGAACATCAAATTTGAAAACGTCTTCCTGACTTACTCGTTGGAGGACCCACCCGTGC

ABCC2_R2 AAGGGAACATCAAATTTGAAAACGTCTTCCTGACTTACTCGTTGGAGGACCCACCCGTGC

ABCC2_R3 AAGGGAACATCAAATTTGAAAACGTCTTCCTGACTTACTCGTTGGAGGACCCACCCGTGC

ABCC2_R4 AAGGGAACATCAAATTTGAAAACGTCTTCCTGACTTACTCGTTGGAGGACCCACCCGTGC

ABCC2_R5 AAGGGAACATCAAATTTGAAAACGTCTTCCTGACTTACTCGTTGGAGGACCCACCCGTGC

**** ******************** **********************************

ABCC2_S TCAAAAATATCAACTTCGAGATCCAAAGCGGGTGGAAGGTGGGCGTGGTCGGCAGGACGG

ABCC2_R1 TCAAAAATATCAACTTCGAGATCCAAAGCGGTTGGAAGGTGGGTGTGGTCGGCAGGACCG

ABCC2_R2 TCAAAAATATCAACTTCGAGATCCAAAGCGGTTGGAAGGTGGGTGTGGTCGGCAGGACCG

ABCC2_R3 TCAAAAATATCAACTTCGAGATCCAAAGCGGTTGGAAGGTGGGTGTGGTCGGCAGGACCG

ABCC2_R4 TCAAAAATATCAACTTCGAGATCCAAAGCGGTTGGAAGGTGGGTGTGGTCGGCAGGACCG

ABCC2_R5 TCAAAAATATCAACTTCGAGATCCAAAGCGGTTGGAAGGTGGGTGTGGTCGGCAGGACCG

******************************* *********** ************** *

**exon 22**

ABCC2_S GGGCCGGGAAGTCATCTCTCATATCTGCCCTTTTCCGATTGACCAATTTAGACGGAAGCA

ABCC2_R1 GGGCCGGGAAGTCATCTCTCATATCTGCCCTTTTCCGATTGACCAATTTAGACGGAAGCA

ABCC2_R2 GGGCCGGGAAGTCATCTCTCATATCTGCCCTTTTCCGATTGACCAATTTAGACGGAAGCA

ABCC2_R3 GGGCCGGGAAGTCATCTCTCATATCTGCCCTTTTCCGATTGACCAATTTAGACGGAAGCA

ABCC2_R4 GGGCCGGGAAGTCATCTCTCATATCTGCCCTTTTCCGATTGACCAATTTAGACGGAAGCA

ABCC2_R5 GGGCCGGGAAGTCATCTCTCATATCTGCCCTTTTCCGATTGACCAATTTAGACGGAAGCA

************************************************************

ABCC2_S TTAAAATTGATGGGATCGATACAATAGGAATAGCTAAGCAGGAGCTCAGGGCGAAGATTT

ABCC2_R1 TTAAAATTGATGGGATCGATACAATAGGAATAGCTAAGCAGGAGCTCAGGGCAAAGATTT

ABCC2_R2 TTAAAATTGATGGGATCGATACAATAGGAATAGCTAAGCAGGAGCTCAGGGCAAAGATTT

ABCC2_R3 TTAAAATTGATGGGATCGATACAATAGGAATAGCTAAGCAGGAGCTCAGGGCAAAGATTT

ABCC2_R4 TTAAAATTGATGGGATCGATACAATAGGAATAGCTAAGCAGGAGCTCAGGGCAAAGATTT

ABCC2_R5 TTAAAATTGATGGGATCGATACAATAGGAATAGCTAAGCAGGAGCTCAGGGCAAAGATTT

**************************************************** *******

**exon 23**

ABCC2_S CAATTATCCCGCAAGAGCCTGTTCTGTTCTCGGCCACGCTGCGCTACAACCTAGACCCCT

ABCC2_R1 CAATTATACCGCAGGAGCCTGTTCTGTTCTCGGCCACGCTGCGCTACAACCTGGACCCCT

ABCC2_R2 CAATTATACCGCAGGAGCCTGTTCTGTTCTCGGCCACGCTGCGCTACAACCTGGACCCCT

ABCC2_R3 CAATTATACCGCAGGAGCCTGTTCTGTTCTCGGCCACGCTGCGCTACAACCTGGACCCCT

ABCC2_R4 CAATTATACCGCAGGAGCCTGTTCTGTTCTCGGCCACGCTGCGCTACAACCTGGACCCCT

ABCC2_R5 CAATTATACCGCAGGAGCCTGTTCTGTTCTCGGCCACGCTGCGCTACAACCTGGACCCCT

******* ***** ************************************** *******

ABCC2_S TCGACTTGTACAGTGACGACGACATTTGGAGAGCTTTGGAGCAGGTGGAGTTGAAAGACG

ABCC2_R1 TCGACTTGTACAGTGATGACGATATTTGGAGAGCTTTGGAACAGGTGGAGTTGAAAGACG

ABCC2_R2 TCGACTTGTACAGTGATGACGATATTTGGAGAGCTTTGGAACAGGTGGAGTTGAAAGACG

ABCC2_R3 TCGACTTGTACAGTGATGACGATATTTGGAGAGCTTTGGAACAGGTGGAGTTGAAAGACG

ABCC2_R4 TCGACTTGTACAGTGATGACGATATTTGGAGAGCTTTGGAACAGGTGGAGTTGAAAGACG

ABCC2_R5 TCGACTTGTACAGTGATGACGATATTTGGAGAGCTTTGGAACAGGTGGAGTTGAAAGACG

**************** ***** ***************** *******************

ABCC2_S TGGTTCCGGCGCTCGACTACAAAGTGTCGGAAGGGGGTAGCAATTTCTCCGTGGGACAGC

ABCC2_R1 TGGTTCCGGCGCTCGACTACAAAGTGTCGGAAGGAGGTAGCAATTTCTCCGTGGGACAGC

ABCC2_R2 TGGTTCCGGCGCTCGACTACAAAGTGTCGGAAGGAGGTAGCAATTTCTCCGTGGGACAGC

ABCC2_R3 TGGTTCCGGCGCTCGACTACAAAGTGTCGGAAGGAGGTAGCAATTTCTCCGTGGGACAGC

ABCC2_R4 TGGTTCCGGCGCTCGACTACAAAGTGTCGGAAGGAGGTAGCAATTTCTCCGTGGGACAGC

ABCC2_R5 TGGTTCCGGCGCTCGACTACAAAGTGTCGGAAGGAGGTAGCAATTTCTCCGTGGGACAGC

********************************** *************************

**exon 24**

ABCC2_S GGCAGCTGCTGTGCCTGGCGCGCGCCGTGCTGCGCTCTAACAAGATACTAGTCATGGACG

ABCC2_R1 GGCAGCTGCTGTGCCTGGCGCGCGCCGTGCTGCGCTCCAACAAGATACTAGTCATGGACG

ABCC2_R2 GGCAGCTGCTGTGCCTGGCGCGCGCCGTGCTGCGCTCCAACAAGATACTAGTCATGGACG

ABCC2_R3 GGCAGCTGCTGTGCCTGGCGCGCGCCGTGCTGCGCTCCAACAAGATACTAGTCATGGACG

ABCC2_R4 GGCAGCTGCTGTGCCTGGCGCGCGCCGTGCTGCGCTCCAACAAGATACTAGTCATGGACG

ABCC2_R5 GGCAGCTGCTGTGCCTGGCGCGCGCCGTGCTGCGCTCCAACAAGATACTAGTCATGGACG

************************************* **********************

**exon 25**

ABCC2_S AGGCCACCGCCAATGTCGACCCGCAGACGGACGCGCTGATCCAGTCGACGATCCGGCGGC

ABCC2_R1 AGGCCACCGCCAATGTCGACCCTCAGACGGACGCACTGATCCAGTCGACGATCCGGCGGC

ABCC2_R2 AGGCCACCGCCAATGTCGACCCTCAGACGGACGCACTGATCCAGTCGACGATCCGGCGGC

ABCC2_R3 AGGCCACCGCCAATGTCGACCCTCAGACGGACGCACTGATCCAGTCGACGATCCGGCGGC

ABCC2_R4 AGGCCACCGCCAATGTCGACCCTCAGACGGACGCACTGATCCAGTCGACGATCCGGCGGC

ABCC2_R5 AGGCCACCGCCAATGTCGACCCTCAGACGGACGCACTGATCCAGTCGACGATCCGGCGGC

********************** *********** *************************

ABCC2_S AGTTCGCGGCCTGCACCGTGCTCACCATCGCGCACCGGCTTAACACCGTCATGGACTCCG

ABCC2_R1 AGTTCGCGGCCTGCACCGTGCTCACCATCGCGCATCGGCTTAACACCGTCATGGACTCCG

ABCC2_R2 AGTTCGCGGCCTGCACCGTGCTCACCATCGCGCATCGGCTTAACACCGTCATGGACTCCG

ABCC2_R3 AGTTCGCGGCCTGCACCGTGCTCACCATCGCGCATCGGCTTAACACCGTCATGGACTCCG

ABCC2_R4 AGTTCGCGGCCTGCACCGTGCTCACCATCGCGCATCGGCTTAACACCGTCATGGACTCCG

ABCC2_R5 AGTTCGCGGCCTGCACCGTGCTCACCATCGCGCATCGGCTTAACACCGTCATGGACTCCG

********************************** *************************

ABCC2_S ACCGAGTGCTCGTCATGGACAAGGGTGAAGTGGTCGAGTTCGACCACCCCTACACGCTGC

ABCC2_R1 ACCGAGTGCTCGTCATGGACAAGGGTGAAGTGGTGGAGTTCGACCACCCGTACACGCTGC

ABCC2_R2 ACCGAGTGCTCGTCATGGACAAGGGTGAAGTGGTGGAGTTCGACCACCCGTACACGCTGC

ABCC2_R3 ACCGAGTGCTCGTCATGGACAAGGGTGAAGTGGTGGAGTTCGACCACCCGTACACGCTGC

ABCC2_R4 ACCGAGTGCTCGTCATGGACAAGGGTGAAGTGGTGGAGTTCGACCACCCGTACACGCTGC

ABCC2_R5 ACCGAGTGCTCGTCATGGACAAGGGTGAAGTGGTGGAGTTCGACCACCCGTACACGCTGC

********************************** ************** **********

**exon 26**

ABCC2_S TGTCAGCCGCCGGCAGCCACCTCAACTTCATGGTGGAGGAGACGGGAGACAACATGAGCA

ABCC2_R1 TGTCAGCCCCCGGCAGCCACCTCAACTTCATGGTGGAAGAAACCGGAGACAACATGAGCA

ABCC2_R2 TGTCAGCCCCCGGCAGCCACCTCAACTTCATGGTGGAAGAAACCGGAGACAACATGAGCA

ABCC2_R3 TGTCAGCCCCCGGCAGCCACCTCAACTTCATGGTGGAAGAAACCGGAGACAACATGAGCA

ABCC2_R4 TGTCAGCCCCCGGCAGCCACCTCAACTTCATGGTGGAAGAAACCGGAGACAACATGAGCA

ABCC2_R5 TGTCAGCCCCCGGCAGCCACCTCAACTTCATGGTGGAAGAAACCGGAGACAACATGAGCA

******** **************************** ** ** ****************

ABCC2_S AGGCCCTTTATGACATGGCCAAGAAGAAATACTTCGATGACCATCCTCAATGA 4044

ABCC2_R1 AGGCCCTTTATGACATGGCCAAGAAGAAATACTTCGATGACCATCCTCAATGA 3909

ABCC2_R2 AGGCCCTTTATGACATGGCCAAGAAGAAATACTTCGATGACCATCCTCAATGA 3888

ABCC2_R3 AGGCCCTTTATGACATGGCCAAGAAGAAATACTTCGACGACCATCCTCAATGA 3992

ABCC2_R4 AGGCCCTTTATGACATGGCCAAGAAGAAATACTTCGACGACCATCCTCAATGA 4831

ABCC2_R5 AGGCCCTTTATGACATGGCCAAGAAGAAATACTTCGACGACCATCCTCAATGA 3240

************************************* ***************
