# Supplementary material for: Resistance to Bacillus thuringiensis Cry1Ac toxin requires mutations in two Plutella xylostella ATP-binding cassette transporter paralogs
Source: PLoS Pathog. 2020 Aug 10;16(8):e1008697. doi: 10.1371/journal.ppat.1008697 (PMC7446926; doi:10.1371/journal.ppat.1008697)
Supplement: S2 Fig — Asterisks denote consensus sequences. Transmembrane domains (TM) (blue), Walker A and B sequences (yellow), C motif (green) and region for antibody recognition (underlined) are shown in the sequence of PxABCC2 protein encoded by ABCC2_S. Deletions are highlighted in gray. (DOC) [file ppat.1008697.s014.doc]

**S2 Fig.**

ABCC2_S MENGSGARKESEEKKEVKKGKPNVLSRLFMCWVCPVLVGGNRRDVEERDLIPPPSAKYKS 60

ABCC2_R1 MENGSGARKESEEKKEVKKGKPNVLSRLFMCWVCPVLVGGNRRDVEERDLIPPPSAKYKS 60

ABCC2_R2 MENGSGARKESEEKKEVKKGKPNVLSRLFMCWVCPVLVGGNRRDVEERDLIPPPSAKYKS 60

ABCC2_R3 MENGSGARKESEEKKEVKKGKPNVLSRLFMCWVCPVLVGGNRRDVEERDLIPPPSAKYKS 60

ABCC2_R4 MENGSGARKESEEKKEVKKGKPNVLSRLFMCWVCPVLVGGNRRDVEERDLIPPPSAKYKS 60

ABCC2_R5 MENGSGARKESEEKKEVKKGKPNVLSRLFMCWVCPVLVGGNRRDVEERDLIPPPSAKYKS 60

************************************************************

**TM1**

ABCC2_S ESLGDKFERYWLEELGLATQRGVSPSLWRALRRAFWLSYMPGALLLLGNAIPRTIQPLLF 120

ABCC2_R1 ESLGDKFERYWLEELGLATQRGVAPSLWRALRRAFWLSYMPGALLLLGNAIPRTIQPLLF 120

ABCC2_R2 ESLGDKFERYWLEELGLATQRGVAPSLWRALRRAFWLSYMPGALLLLGNAIPRTIQPLLF 120

ABCC2_R3 ESLGDKFERYWLEELGLATQRGVAPSLWRALRRAFWLSYMPGALLLLGNAIPRTIQPLLF 120

ABCC2_R4 ESLGDKFERYWLEELGLATQRGVAPSLWRALRRAFWLSYMPGALLLLGNAIPRTIQPLLF 120

ABCC2_R5 ESLGDKFERYWLEELGLATQRGVAPSLWRALRRAFWLSYMPGALLLLGNAIPRTIQPLLF 120

***********************:************************************

**TM2**

ABCC2_S TRLLSYWSADSTMTRLEAGYWAMGMLLCNFLAMVCHHHNTLFVGRFGMKVRIACCSLLYR 180

ABCC2_R1 TRLLSYWSADSTMTRLEAGYWAMGMLLCNFLAMVCHHHNTLFVGRFGMKVRIACCSLLYR 180

ABCC2_R2 TRLLSYWSADSTMTRLEAGYWAMGMLLCNFLAMVCHHHNTLFVGRFGMKVRIACCSLLYR 180

ABCC2_R3 TRLLSYWSADSTMTRLEAGYWAMGMLLCNFLAMVCHHHNTLFVGRFGMKVRIACCSLLYR 180

ABCC2_R4 TRLLSYWSADSTMTRLEAGYWAMGMLLCNFLAMVCHHHNTLFVGRFGMKVRIACCSLLYR 180

ABCC2_R5 TRLLSYWSADSTMTRLEAGYWAMGMLLCNFLAMVCHHHNTLFVGRFGMKVRIACCSLLYR 180

************************************************************

**TM3**

ABCC2_S KLLRLNQRSLQSTAAGKLVNLMSNDVARFDYAFMFLHYFWMIPLQSAAVLYFMFRAAGWA 240

ABCC2_R1 KLLRLNQRSLQSTAAGKLVNLMSNDVARFDYAFMFLHYFWMIPLQSAAVLYFMFRAAGWA 240

ABCC2_R2 KLLRLNQRSLQSTAAGKLVNLMSNDVARFDYAFMFLHYFWMIPLQSAAVLYFMFRAAGWA 240

ABCC2_R3 KLLRLNQRSLQSTAAGKLVNLMSNDVARFDYAFMFLHYFWMIPLQSAAVLYFMFRAAGWA 240

ABCC2_R4 KLLRLNQRSLQSTAAGKLVNLMSNDVARFDYAFMFLHYFWMIPLQSAAVLYFMFRAAGWA 240

ABCC2_R5 KLLRLNQRSLQSTAAGKLVNLMSNDVARFDYAFMFLHYFWMIPLQSAAVLYFMFRAAGWA 240

************************************************************

**TM4**

ABCC2_S PIVGLFSVMLLILPIQAGLTKLTAVYRRETAQRTDKRIKLMGEIINGIQVIKMYAWEVPF 300

ABCC2_R1 PIVGLFSVMLLILPIQAGLTKLTAVYRRETAQRTDKRIKLMSEIINGDKDVRVGGPLPEG 300

ABCC2_R2 PIVGLFSVMLLILPIQAGLTKLTAVYRRETAQRTDKRIKLMSEIINGIQVIKMYAWEVPF 300

ABCC2_R3 PIVGLFSVMLLILPIQAGLTKLTAVYRRETAQRTDKRIKLMSEIINGIQVIKMYAWEVPF 300

ABCC2_R4 PIVGLFSVMLLILPIQAGLTKLTAVYRRETAQRTDKRIKLMSEIINGIQVIKMYAWEVPF 300

ABCC2_R5 PIVGLFSVMLLILPIQAGLTKLTAVYRRETAQRTDKRIKLMSEIINGIQ----------- 289

*****************************************.*****

**TM5**

ABCC2_S QKVVGSSRAHEVEALKRASFVQGTFGGFMLFTERTSLFLTVMTLVLTGSMATATTVYPIQ 360

ABCC2_R1 GGVLPRARGGGVE---AGVLRAGHLRGVH------------------------------- 326

ABCC2_R2 QKVVGSSRAHEVEALKRASFVQGTFGGFMLFTERTSLFLTVMTLVLTGSMATATTTRGKT 360

ABCC2_R3 QKVVGSSRAHEVEALKRASFVQGTFGGFMLFTERTSLFLTVMTLVLTGSMATATTTRGKT 360

ABCC2_R4 QKVVGSSRAHEVEALKRASFVQGTFGGFMLFTERTSLFLTVMTLVLTGSMATATTTRGKT 360

ABCC2_R5 ------------------------------------------------------------ 289

**TM6**

ABCC2_S QYFSIIQSNLALILPIAIAQLTEMLVSLERLQEFLMLDEREDLSVMPGGQADTAPVAFKY 420

ABCC2_R1 ---AVHGA--HLALPH------RHDARADWEHGYCYYDEREDLSVMPGGQADTAPVAFKY 375

ABCC2_R2 CR---------------------------------------------------------- 362

ABCC2_R3 CR---------------------------------------------------------- 362

ABCC2_R4 CR---------------------------------------------------------- 362

ABCC2_R5 ------------------------------------------------------------ 289

ABCC2_S TKETTAPAYIVSKRYSKKEDDTGLAAELVERKSTSEFAVELNDVSASWGGEGDKDQHTLR 480

ABCC2_R1 TKETTAPAYIVSKRYSKKEDDTGLAAELVERKATSEFAVELNDVSASWGGEGDKDEHTLR 435

ABCC2_R2 ------------------------------------------------------------ 362

ABCC2_R3 ------------------------------------------------------------ 362

ABCC2_R4 ------------------------------------------------------------ 362

ABCC2_R5 ------------------------------------------------------------ 289

**Walker A**

ABCC2_S GVSMRVRRGKLAAIIGPVGSGKSSLLQVLLKELPVSSGSVGVHGQISYACQESWLFSATI 540

ABCC2_R1 GVSMRVRRGKLAAIIGPVGSGKSSLLQVLLKELPVSSGTVGVHGQISYACQESWLFSATV 495

ABCC2_R2 ------------------------------------------------------------ 362

ABCC2_R3 ------------------------------------------------------------ 362

ABCC2_R4 ------------------------------------------------------------ 362

ABCC2_R5 ------------------------------------------------------------ 289

**C motif**

ABCC2_S RDNILFGLPYDSKKYKKVCDACCLQPDFKQFPYGDLSLVGERGVSLSGGQRARINLARAV 600

ABCC2_R1 RDNILFGLPYDSKKYKKVCDACCLQPDFKQFPYGDLSLVGERGVSLSGGQRARINLARAV 555

ABCC2_R2 ------------------------------------------------------------ 362

ABCC2_R3 ------------------------------------------------------------ 362

ABCC2_R4 ------------------------------------------------------------ 362

ABCC2_R5 -----------------VCDACCLQPDFKQFPYGDLSLVGERGVSLSGGQRARINLARAV 332

**Walker B**

ABCC2_S YRDADIYIFDDPLSAVDANVGRQLFEGCINGYLRGRTRVLVTHQIHFLKAADYIVILNEG 660

ABCC2_R1 YRDADIYIFDDPLSAVDANVGRQLFEGCINGYLRGRTRVLVTHQIHFLKAADYIVILNEG 615

ABCC2_R2 ------------------------------------------------------------ 362

ABCC2_R3 ------------------------------------------------------------ 362

ABCC2_R4 ------------------------------------------------------------ 362

ABCC2_R5 YRDADIYIFDDPLSAVDANVGRQLFEGCINGYLRGRTRVLVTHQIHFLKAADYIVILNEG 392

ABCC2_S AIENMGTYDDLTKLENSLLLPKQQEGSGDDSKDELAIPNAAKKPIMERGVSVISVKSEDN 720

ABCC2_R1 AIENMGTYDDLTKLENSLLLPKQQEGSGDDSKGELAIPNAAKKPIVERGISVISVKSEDN 675

ABCC2_R2 ------------------------------------------------------------ 362

ABCC2_R3 ------------------------------------------------------------ 362

ABCC2_R4 ------------------------------------------------------------ 362

ABCC2_R5 AIENMGTYDDLTKLENSLLLPKQQEGSGDDSKGELAIPNAAKKPIVERGISVISVKSEDN 452

**TM7**

ABCC2_S GEARKEQVQAAEERASGNLKWEVFARYLVSVDSWAIVALTLTAMLITQGAASSTDYWLSF 780

ABCC2_R1 GEARKEQIQAAEERASGNLKWEVFARYLVSVDSWAIVALTLTAMLITQGAASSTDYWLSF 735

ABCC2_R2 ------------------------------------------------------------ 362

ABCC2_R3 ------------------------------------------------------------ 362

ABCC2_R4 ------------------------------------------------------------ 362

ABCC2_R5 GEARKEQIQAAEERASGNLKWEVFARYLVSVDSWAIVALTLTAMLITQGAASSTDYWLSF 512

**TM8**

ABCC2_S WTNQVDGYIQDLPDGEEPDPSLGTQTGILQTGQYVYIYGALVLTIIVMSFMRLFGFVTMT 840

ABCC2_R1 WTNQVDGYIQDLPDGEEPDPSLGTQTGILETGQYVYIYGALVLTIIVMSFMRLFGFVTMT 795

ABCC2_R2 ------------------------------------------------------------ 362

ABCC2_R3 ------------------------------------------------------------ 362

ABCC2_R4 ------------------------------------------------------------ 362

ABCC2_R5 WTNQVDGYIQDLPDGEEPDPSLGTQTGILETGQYVYIYGALVLTIIVMSFMRLFGFVTMT 572

**TM9**

ABCC2_S MRAAANIHDLMFRNLIRATMRFFDTNPSGRVLNRFSKDMGGMDELLPRSILQAFQMYLSM 900

ABCC2_R1 MRAAANIHDLMFRNLIRATMRFFDTNPSGRVLNRFSKDMGGMDELLPRSILQAFQMYLSM 855

ABCC2_R2 ------------------------------------------------------------ 362

ABCC2_R3 ------------------------------------------------------------ 362

ABCC2_R4 ------------------------------------------------------------ 362

ABCC2_R5 MRAAANIHDLMFRNLIRATMRFFDTNPSGRVLNRFSKDMGGMDELLPRSILQAFQMYLSM 632

**TM10**

ABCC2_S ASVLTLNAVSLPWTLIPTVLLLGLFIRYLKWYLNAAQSVKRLEGTTKSPVFGMIGSTLSG 960

ABCC2_R1 ASVLTLNAVSLPWTLIPTVLLLGLFIRYLKWYLNAAQSVKRLEGTTKSPVFGMIGSTLSG 915

ABCC2_R2 ------------------------------------------------------------ 362

ABCC2_R3 ------------------------------------------------------------ 362

ABCC2_R4 ------------------------------------------------------------ 362

ABCC2_R5 ASVLTLNAVSLPWTLIPTVLLLGLFIRYLKWYLNAAQSVKRLEGTTKSPVFGMIGSTLSG 692

**TM11**

ABCC2_S MSTIRSSDSQDRLIKSFDDCQNLHTSAFHTYIGGATAFGFYLDMICLVYLASILSIFILI 1020

ABCC2_R1 MSTIRSSDSQDRLIKNFDDCQNLHTSAFHTYIGGATAFGFYLDMICLVYLASILSIFILI 975

ABCC2_R2 ------------------------------------------------------------ 362

ABCC2_R3 ------------------------------------------------------------ 362

ABCC2_R4 ------------------------------------------------------------ 362

ABCC2_R5 MSTIRSSDSQDRLIKNFDDCQNLHTSAFHTYIGGATAFGFYLDMICLVYLASILSIFILI 752

**TM12**

ABCC2_S DFADVIPVGSVGLAVSQSMVLTVLLQLAARFTSDFLAQMTAVERVLEYTKLPHEENINDG 1080

ABCC2_R1 DFADVIPVGSVGLAVSQSMVLTVLLQLAARFTSDFLAQMTAVERVLEYTKLPHEENINDG 1035

ABCC2_R2 ------------------------------------------------------------ 362

ABCC2_R3 ------------------------------------------------------------ 362

ABCC2_R4 ------------------------------------------------------------ 362

ABCC2_R5 DFADVIPVGSVGLAVSQSMVLTVLLQLAARFTSDFLAQMTAVERVLEYTKLPHEENINDG 812

**Walker A**

ABCC2_S PTQPPKTWPAEGNIKFENVFLTYSLEDPPVLKNINFEIQSGWKVGVVGRTGAGKSSLISA 1140

ABCC2_R1 PTHPPKTWPAEGNIKFENVFLTYSLEDPPVLKNINFEIQSGWKVGVVGRTGAGKSSLISA 1095

ABCC2_R2 ------------------------------------------------------------ 362

ABCC2_R3 ------------------------------------------------------------ 362

ABCC2_R4 ------------------------------------------------------------ 362

ABCC2_R5 PTHPPKTWPAEGNIKFENVFLTYSLEDPPVLKNINFEIQSGWKVGVVGRTGAGKSSLISA 872

ABCC2_S LFRLTNLDGSIKIDGIDTIGIAKQELRAKISIIPQEPVLFSATLRYNLDPFDLYSDDDIW 1200

ABCC2_R1 LFRLTNLDGSIKIDGIDTIGIAKQELRAKISIIPQEPVLFSATLRYNLDPFDLYSDDDIW 1155

ABCC2_R2 ------------------------------------------------------------ 362

ABCC2_R3 ------------------------------------------------------------ 362

ABCC2_R4 ------------------------------------------------------------ 362

ABCC2_R5 LFRLTNLDGSIKIDGIDTIGIAKQELRAKISIIPQEPVLFSATLRYNLDPFDLYSDDDIW 932

**C motif Walker B**

ABCC2_S RALEQVELKDVVPALDYKVSEGGSNFSVGQRQLLCLARAVLRSNKILVMDEATANVDPQT 1260

ABCC2_R1 RALEQVELKDVVPALDYKVSEGGSNFSVGQRQLLCLARAVLRSNKILVMDEATANVDPQT 1215

ABCC2_R2 ------------------------------------------------------------ 362

ABCC2_R3 ------------------------------------------------------------ 362

ABCC2_R4 ------------------------------------------------------------ 362

ABCC2_R5 RALEQVELKDVVPALDYKVSEGGSNFSVGQRQLLCLARAVLRSNKILVMDEATANVDPQT 992

ABCC2_S DALIQSTIRRQFAACTVLTIAHRLNTVMDSDRVLVMDKGEVVEFDHPYTLLSAAGSHLNF 1320

ABCC2_R1 DALIQSTIRRQFAACTVLTIAHRLNTVMDSDRVLVMDKGEVVEFDHPYTLLSAPGSHLNF 1275

ABCC2_R2 ------------------------------------------------------------ 362

ABCC2_R3 ------------------------------------------------------------ 362

ABCC2_R4 ------------------------------------------------------------ 362

ABCC2_R5 DALIQSTIRRQFAACTVLTIAHRLNTVMDSDRVLVMDKGEVVEFDHPYTLLSAPGSHLNF 1052

ABCC2_S MVEETGDNMSKALYDMAKKKYFDDHPQ 1347

ABCC2_R1 MVEETGDNMSKALYDMAKKKYFDDHPQ 1302

ABCC2_R2 --------------------------- 362

ABCC2_R3 --------------------------- 362

ABCC2_R4 --------------------------- 362

ABCC2_R5 MVEETGDNMSKALYDMAKKKYFDDHPQ 1079
