# Supplementary material for: Resistance to Bacillus thuringiensis Cry1Ac toxin requires mutations in two Plutella xylostella ATP-binding cassette transporter paralogs
Source: PLoS Pathog. 2020 Aug 10;16(8):e1008697. doi: 10.1371/journal.ppat.1008697 (PMC7446926; doi:10.1371/journal.ppat.1008697)
Supplement: S3 Fig — Asterisks denote consensus sequences. Exons (yellow) and introns (gray) are shown in the sequence of gDNA_PF1. Deletions in cDNA are highlighted in green. Letter highlighted in red indicates the point mutation in gDNA. The primer sequences are underlined in red. (DOC) [file ppat.1008697.s015.doc]

**S3 Fig.**

**exon 5**

gDNA_PF1 GCACAAAGGACAGATAAGAGAATTAAACTAATGAGCGAAATTATCAATGGTATTCAGGTA

ABCC2_R1 GCACAAAGGACAGATAAGAGAATTAAACTAATGAGCGAAATTATCAATG-----------

*************************************************

gDNA_PF1 TTGTTACTTTATTAAATTAATATGGTTGTGCTTATTTTATATCTATCTGAGTCAGAACTT

ABCC2_R1 ------------------------------------------------------------

**intron 5**

gDNA_PF1 ACTAACTAAATTTATTAACACTCAAAGAATATAAACATGACAAATAACTATTTTAAGACT

ABCC2_R1 ------------------------------------------------------------

gDNA_PF1 AGCTAATCTTTTCACCACTGTGTCCAGCTCTATAAACCAACTAAGACCACCTATACCATA

ABCC2_R1 ------------------------------------------------------------

gDNA_PF1 CAATCTTTACAACCGACCAACTGTTTCCAGGTGATAAAGATGTACGCGTGGGAGGTCCCC

ABCC2_R1 ------------------------------GTGATAAAGATGTACGCGTGGGAGGTCCCC

******************************

gDNA_PF1 TTCCAGAAGGTGGTGGGGTCCTCCCGCGCGCACGAGGTGGAGGCGTTGAAGCGGGCGTCC

ABCC2_R1 TTCCAGAAGGTGGTGGGGTCCTCCCGCGCGCACGAGGTGGAGGCGTTGAAGCGGGCGTCC ************************************************************

**exon 6**

gDNA_PF1 TTCGTGCAGGGCACCTTCGGGGGGTTCATGCTGTTCACGGAGCGCACCTCGCTCTTCCTC

ABCC2_R1 TTCGTGCAGGGCACCTTCGGGGGGTTCATGCTGTTCACGGAGCGCACCTCGCTCTTCCTC ************************************************************

gDNA_PF1 ACCGTCATGACGCTCGTGCTGACTGGGAGCATGGCTACTGCTACTACGGTTAGTGTTACT

ABCC2_R1 ACCGTCATGACGCTCGTGCTGACTGGGAGCATGGCTACTGCTACTACG------------

************************************************

gDNA_PF1 TACTAATTACATATAATACACATACTAATAGACTCACAAGAGTCACGAGCGATAGTATTA

ABCC2_R1 ------------------------------------------------------------

gDNA_PF1 TGGTACTGAAGTGGCTGGTGGAACTTTTTCATTGCACGTGAGTGTATAATGAATCCCTAA

ABCC2_R1 ------------------------------------------------------------

gDNA_PF1 GTATGTGTGCACTTATAAGTATAGTTAACTCTTGCATGCCTTACATGCATTCGTAGATGT

ABCC2_R1 ------------------------------------------------------------

**intron 6**

gDNA_PF1 GCTGCTGGTGCAAACTGCCGAACGATATCAGATTAAACTTTTCAGTTTTACAGAGCAGTG

ABCC2_R1 ------------------------------------------------------------

gDNA_PF1 ACTCACTTATTTGACCAAAAATGTGTGGCGATACAATATTGTTAATAGAGCAGTGCAAGA

ABCC2_R1 ------------------------------------------------------------

gDNA_PF1 GTCGAAAGTAATCAATGATTCTGTTCACGCTACATCGATTAATATACTTATGTGCTGTAG

ABCC2_R1 ------------------------------------------------------------

gDNA_PF1 GCTGAAAAAAATGGACTAAATTGTTATTACGTACATCACATTTATGTTTTGTTACATGTG

ABCC2_R1 ------------------------------------------------------------

**exon 7**

gDNA_PF1 TATCCAATCCAACAATACTTTAGTATAATCCAATCCAATTTGGCGCTCATCCTCCCCATC

ABCC2_R1 ------------------------------------------------------------

gDNA_PF1 GCCATCGCTCAACTGACAGAGATGTTGGTTTCATTGGAAAGACTTCAGGAGTTTCTGATG

ABCC2_R1 ------------------------------------------------------------

gDNA_PF1 TTGGGTAAGCCATTTCTCAGCTTATCTGAACCAGTAAAGACTAATTACTTATTACTTCTG

ABCC2_R1 ------------------------------------------------------------

gDNA_PF1 GATTACACTCTATGGTTGATTGGTTTGACGTACTCATCGATAACGATTCTATACCGCGCT

ABCC2_R1 ------------------------------------------------------------

**intron 7**

gDNA_PF1 ATATCATTTCCGGGCTTGGATAAAACATGCTGCACACGTAGGTTTTGGCTTAGTATACCC

ABCC2_R1 ------------------------------------------------------------

gDNA_PF1 TTTTTTCCTGTACACCTCATAATTAGTCGCTTTATAATGAACCTGTAATTATCATTCTAG

ABCC2_R1 ------------------------------------------------------------

**exon 8**

gDNA_PF1 ACGAGAGGGAAGACCTGTCGGTGATGCCGGGCGGGCAGGCGGACACCGCGCCCGTGGCCT

ABCC2_R1 ACGAGAGGGAAGACCTGTCGGTGATGCCGGGCGGGCAGGCGGACACCGCGCCCGTGGCCT ************************************************************

gDNA_PF1 TCAAGTACACGAAGGAGACCACG

ABCC2_R1 TCAAGTACACGAAGGAGACCACG

***********************
