# Supplementary material for: Resistance to Bacillus thuringiensis Cry1Ac toxin requires mutations in two Plutella xylostella ATP-binding cassette transporter paralogs
Source: PLoS Pathog. 2020 Aug 10;16(8):e1008697. doi: 10.1371/journal.ppat.1008697 (PMC7446926; doi:10.1371/journal.ppat.1008697)
Supplement: S4 Fig — Asterisks denote consensus sequences. Exons (yellow) and introns (gray) are shown in the fragment sequence of gDNA_PF2. Deletions and insertions in cDNA are respectively highlighted in green and blue. The primer sequences are underlined in red. (DOC) [file ppat.1008697.s016.doc]

**S4 Fig.**

gDNA_PF2 ACCTCAAGTGGGAGGTGTTCGCGAGGTACCTGGTCTCCGTGGACTCCTGGGCCATCGTGG

ABCC2_R2 ACCTCAAGTGGGAGGTGTTCGCGAGGTACCTGGTCTCCGTGGACTCCTGGGCCATCGTGG

ABCC2_R4 ACCTCAAGTGGGAGGTGTTCGCGAGGTACCTGGTCTCCGTGGACTCCTGGGCCATCGTGG

************************************************************

**exon 14**

gDNA_PF2 CGCTCACGCTCACCGCGATGCTCATCACCCAGGGGGCGGCGTCGTCCACCGACTACTGGC

ABCC2_R2 CGCTCACGCTCACCGCGATGCTCATCACCCAGGGGGCGGCGTCGTCCACCGACTACTGGC

ABCC2_R4 CGCTCACGCTCACCGCGATGCTCATCACCCAGGGGGCGGCGTCGTCCACCGACTACTGGC

************************************************************

**intron 14**

gDNA_PF2 TTAGCTTCTGGTAAATATGAATCAAGAGTTGTGTTAGTGATACTGAAGTATTAGCTGAGA

ABCC2_R2 TTAGCTTCTG--------------------------------------------------

ABCC2_R4 TTAGCTTCTG--------------------------------------------------

**********

**exon 15**

gDNA_PF2 TTAATGTGTTTTTGTTTTTAGGACAAATCAAGTTGATGGATACATACAAGACCTGCCAGA

ABCC2_R2 --------------------------------------------------ACCTGCCAGA

ABCC2_R4 ---------------------GACAAATCAAGTTGATGGATACATACAAGACCTGCCAGA

**********

gDNA_PF2 TGGGGAGGAACCAGGTGAGTTCTTACTTGTTATACCTGAAAGAGCCAAAAGGGTAGTGAT

ABCC2_R2 TGGGGAGGAACCAG----------------------------------------------

ABCC2_R4 TGGGGAGGAACCAGGTGAGTTCTTACTTGTTATACCTGAAAGAGCCAAAAGGGTAGTGAT

**************

gDNA_PF2 ACCAATACCAGAATATCATCGCCTTGGAGATCAATTACTCTGACCCACAAATTTTGTGAA

ABCC2_R2 ------------------------------------------------------------

ABCC2_R4 ACCAATACCAGAATATCATCGCCTTGGAGATCAATTACTCTGACCCACAAATTTTGTGAA

**intron 15**

gDNA_PF2 AAATAAGTTAGGTATACCAAACGATTCAAAATCGATTAAGTGTACAGTGGACAATCAATC

ABCC2_R2 ------------------------------------------------------------

ABCC2_R4 AAATAAGTTAGGTATACCAAACGATTCAAAATCGATTAAGTGTACAGTGGACAATCAATC

gDNA_PF2 TAAGCCTCAAAAAAAATTGTGGGTTGGACTAATTGACCTCCAAGGCGAGGACATGAAAGT

ABCC2_R2 ------------------------------------------------------------

ABCC2_R4 TAAGCCTCAAAAAAAATTGTGGGTTGGACTAATTGACCTCCAAGGCGAGGACATGAAAGT

gDNA_PF2 AAAATTTTGTACAATATGTTGTATTCCCAGATCCAAGTCTCGGCACGCAAACAGGCATCC

ABCC2_R2 ------------------------------ATCCAAGTCTCGGCACGCAAACAGGCATCC

ABCC2_R4 AAAATTTTGTACAATATGTTGTATTCCCAGATCCAAGTCTCGGCACGCAAACAGGCATCC

******************************

**exon 16**

gDNA_PF2 TGGAGACGGGCCAGTACGTGTACATCTACGGCGCGCTGGTGCTGACCATAATCGT

ABCC2_R2 TGGAGACGGGCCAGTACGTGTACATCTACGGCGCGCTGGTGCTGACCATAATCGT

ABCC2_R4 TGGAGACGGGCCAGTACGTGTACATCTACGGCGCGCTGGTGCTGACCATAATCGT

*******************************************************
