# Supplementary material for: Resistance to Bacillus thuringiensis Cry1Ac toxin requires mutations in two Plutella xylostella ATP-binding cassette transporter paralogs
Source: PLoS Pathog. 2020 Aug 10;16(8):e1008697. doi: 10.1371/journal.ppat.1008697 (PMC7446926; doi:10.1371/journal.ppat.1008697)
Supplement: S5 Fig — Exons (yellow) and introns (gray) are shown in the sequence of gDNA_PF3. Insertions in cDNA are highlighted in blue. The primer sequences are underlined in red. (DOC) [file ppat.1008697.s017.doc]

**S5 Fig.**

**exon 11**

gDNA_PF3 ACTTCAAGCAGTTCCCGTACGGAGACCTGTCGCTGGTGGGCGAGCGCGGCGTGTCGCTGT

ABCC2_R3 ACTTCAAGCAGTTCCCGTACGGAGACCTGTCGCTGGTGGGCGAGCGCGGCGTGTCGCTGT

ABCC2_R4 ACTTCAAGCAGTTCCCGTACGGAGACCTGTCGCTGGTGGGCGAGCGCGGCGTGTCGCTGT

************************************************************

gDNA_PF3 CCGGGGGGCAGCGCGCCCGGATCAACCTGGCCCGCGCCGTGTACCGGGACGTATGTATAC

ABCC2_R3 CCGGGGGGCAGCGCGCCCGGATCAACCTGGCCCGCGCCGTGTACCGGGAC----------

ABCC2_R4 CCGGGGGGCAGCGCGCCCGGATCAACCTGGCCCGCGCCGTGTACCGGGACGTATGTATAC

***************************************************

gDNA_PF3 ACACAAACACGGAGACACACAGACATCGCACTACCTAGGAACAAAAAGTACCTAATAGAA

ABCC2_R3 ------------------------------------------------------------

ABCC2_R4 ACACAAACACGGAGACACACAGACATCGCACTACCTAGGAACAAAAAGTACCTAATAGAA

gDNA_PF3 AAAAAGCTCTAGCTATTATTCTAGAGATGCGACACCTTAAAAAATAAACCACTGGAAACT

ABCC2_R3 ------------------------------------------------------------

ABCC2_R4 AAAAAGCTCTAGCTATTATTCTAGAGATGCGACACCTTAAAAAATAAACCACTGGAAACT

**intron 11**

gDNA_PF3 GAATATCTTTGATCTAAGTAAAACTTACTGAAACTATTCCACCCTAAGTTGCTCAATATC

ABCC2_R3 ------------------------------------------------------------

ABCC2_R4 GAATATCTTTGATCTAAGTAAAACTTACTGAAACTATTCCACCCTAAGTTGCTCAATATC

gDNA_PF3 CGTGTTATTTTTCTTTATAAAAGTTGAACAGAAGAAAAATACGCGAGGAGAATGGGCTTA

ABCC2_R3 ------------------------------------------------------------

ABCC2_R4 CGTGTTATTTTTCTTTATAAAAGTTGAACAGAAGAAAAATACGCGAGGAGAATGGGCTTA

gDNA_PF3 GAAAACTTTTAAGTTAGTTATTAAACAATGGCTTAGCAAGTTAGCAACCTATCTACATTT

ABCC2_R3 ------------------------------------------------------------

ABCC2_R4 GAAAACTTTTAAGTTAGTTATTAAACAATGGCTTAGCAAGTTAGCAACCTATCTACATTT

gDNA_PF3 CAGGCTGACATCTACATATTCGATGACCCCCTATCGGCGGTGGACGCGAATGTAGGTCGG

ABCC2_R3 ---GCTGACATCTACATATTCGATGACCCCCTATCGGCGGTGGACGCGAATGTAGGTCGG

ABCC2_R4 CAGGCTGACATCTACATATTCGATGACCCCCTATCGGCGGTGGACGCGAATGTAGGTCGG

********************************************************

**exon 12**

gDNA_PF3 CAGCTGTTCGAGGGCTGCATCAACGGCTACCTGCGCGGCCGCACGCGCGTGCTCGTCACG

ABCC2_R3 CAGCTGTTCGAGGGCTGCATCAACGGCTACCTGCGCGGCCGCACGCGCGTGCTCGTCACG

ABCC2_R4 CAGCTGTTCGAGGGCTGCATCAACGGCTACCTGCGCGGCCGCACGCGCGTGCTCGTCACG

************************************************************

gDNA_PF3 CATCAGATACACTTCCTCAAGGCCGCAGACTACATAGTCATACTCAACGAGGTAATTGCA

ABCC2_R3 CATCAGATACACTTCCTCAAGGCCGCAGACTACATAGTCATACTCAACGAGGTAATTGCA

ABCC2_R4 CATCAGATACACTTCCTCAAGGCCGCAGACTACATAGTCATACTCAACGAG---------

***************************************************

**intron 12**

gDNA_PF3 CACACAATCTTACACGTGTAATGTTAAATGAAGCGTTTAATAACATGATTGTTTACCTTG

ABCC2_R3 CACACAATCTTACACGTGTAATGTTAAATGAAGCGTTTAATAACATGATTGTTTACCTTG

ABCC2_R4 ------------------------------------------------------------

**exon 13**

gDNA_PF3 GACCAGGGTGCCATTGAAAATATGGGCACGTACGATGATTTAACGAAGCTGGAGAATTCG

ABCC2_R3 GACCAGGGTGCCATTGAAAATATGGGCACGTACGATGATTTAACGAAGCTGGAGAATTCG

ABCC2_R4 ------GGTGCCATTGAAAATATGGGCACGTACGATGATTTAACGAAGCTGGAGAATTCG

******************************************************

gDNA_PF3 CTGCTG

ABCC2_R3 CTGCTG

ABCC2_R4 CTGCTG

******
