# Supplementary material for: Resistance to Bacillus thuringiensis Cry1Ac toxin requires mutations in two Plutella xylostella ATP-binding cassette transporter paralogs
Source: PLoS Pathog. 2020 Aug 10;16(8):e1008697. doi: 10.1371/journal.ppat.1008697 (PMC7446926; doi:10.1371/journal.ppat.1008697)
Supplement: S6 Fig — Asterisks denote consensus sequences. Exons (yellow) and introns (gray) are shown in the fragment sequence of gDNA_PF4. Insertions in cDNA are highlighted in blue. The primer sequences are underlined in red. (DOC) [file ppat.1008697.s018.doc]

**S6 Fig.**

**exon 16**

gDNA_PF4 CCAACATCCACGACCTCATGTTCCGCAACCTCATCCGCGCCACCATGCGCTTCTTTGACA

ABCC2_R4 CCAACATCCACGACCTCATGTTCCGCAACCTCATCCGCGCCACCATGCGCTTCTTTGACA

************************************************************

gDNA_PF4 CCAATCCTTCGGGTACTGTATTGGTTTATTCTGTCGTTCTGATGACGTACTTATCTAAAT

ABCC2_R4 CCAATCCTTCGGGTACTGTATTGGTTTATTCTGTCGTTCTGATGACGTACTTATCTAAAT

************************************************************

gDNA_PF4 TCATGCTGGGAATGCAGCGCACTGGACACTAAGATAAAAGACCTGGAAGACCTAACTAAC

ABCC2_R4 TCATGCTGGGAATGCAGCGCACTGGACACTAAGATAAAAGACCTGGAAGACCTAACTAAC

************************************************************

gDNA_PF4 TGACGAATCCTTTCACAACGATGAAACTAACTCCGAATGTATTTGCGTACACAAATACCC

ABCC2_R4 TGACGAATCCTTTCACAACGATGAAACTAACTCCGAATGTATTTGCGTACACAAATACCC

************************************************************

**intron 16**

gDNA_PF4 TCAAAAGGTACAGGTTATAATAAGTTTCCATCAAAATGACAAAGATGATTTAAATTCACA

ABCC2_R4 TCAAAAGGTACAGGTTATAATAAGTTTCCATCAAAATGACAAAGATGATTTAAATTCACA

************************************************************

gDNA_PF4 TAGTCATCAGACCTAACCACCCGGAAGAACCGTTATCATCATCTCACACACAACCAATTC

ABCC2_R4 TAGTCATCAGACCTAACCACCCGGAAGAACCGTTATCATCATCTCACACACAACCAATTC

************************************************************

gDNA_PF4 ACAGATCTTATACAGTATGCAGCTCTTATTCACTTGTTTACAACACTCCATCTATAGGTC

ABCC2_R4 ACAGATCTTATACAGTATGCAGCTCTTATTCACTTGTTTACAACACTCCATCTATAGGTC

************************************************************

gDNA_PF4 GCGTGCTAAACCGGTTCTCCAAAGACATGGGCGGTATGGACGAGCTGCTGCCGAGGTCCA

ABCC2_R4 GCGTGCTAAACCGGTTCTCCAAAGACATGGGCGGTATGGACGAGCTGCTGCCGAGGTCCA

************************************************************

**exon 17**

gDNA_PF4 TCCTGCAGGCCTTCCAGATGTACCTGTCCATGGCGAGCGTGCTCACGCTGAACGCCGTCT

ABCC2_R4 TCCTGCAGGCCTTCCAGATGTACCTGTCCATGGCGAGCGTGCTCACGCTGAACGCCGTCT

************************************************************

gDNA_PF4 CCCTGCCCTGGACCCTCATACCCACGGTGCTGCTGCTGGGCCTCTTCATCAGGTACCTCA

ABCC2_R4 CCCTGCCCTGGACCCTCATACCCACGGTGCTGCTGCTGGGCCTCTTCATCAGGTACCTCA

************************************************************

gDNA_PF4 AGTGGTACCTGAACGCTGCGCAGTCTGTGAAGAGGCTGGAGGGTA

ABCC2_R4 AGTGGTACCTGAACGCTGCGCAGTCTGTGAAGAGGCTGGAGGGTA

*********************************************
