# Supplementary figures and images for: Resistance to Bacillus thuringiensis Cry1Ac toxin requires mutations in two Plutella xylostella ATP-binding cassette transporter paralogs
Source: PLoS Pathog. 2020 Aug 10;16(8):e1008697. doi: 10.1371/journal.ppat.1008697 (PMC7446926; doi:10.1371/journal.ppat.1008697)

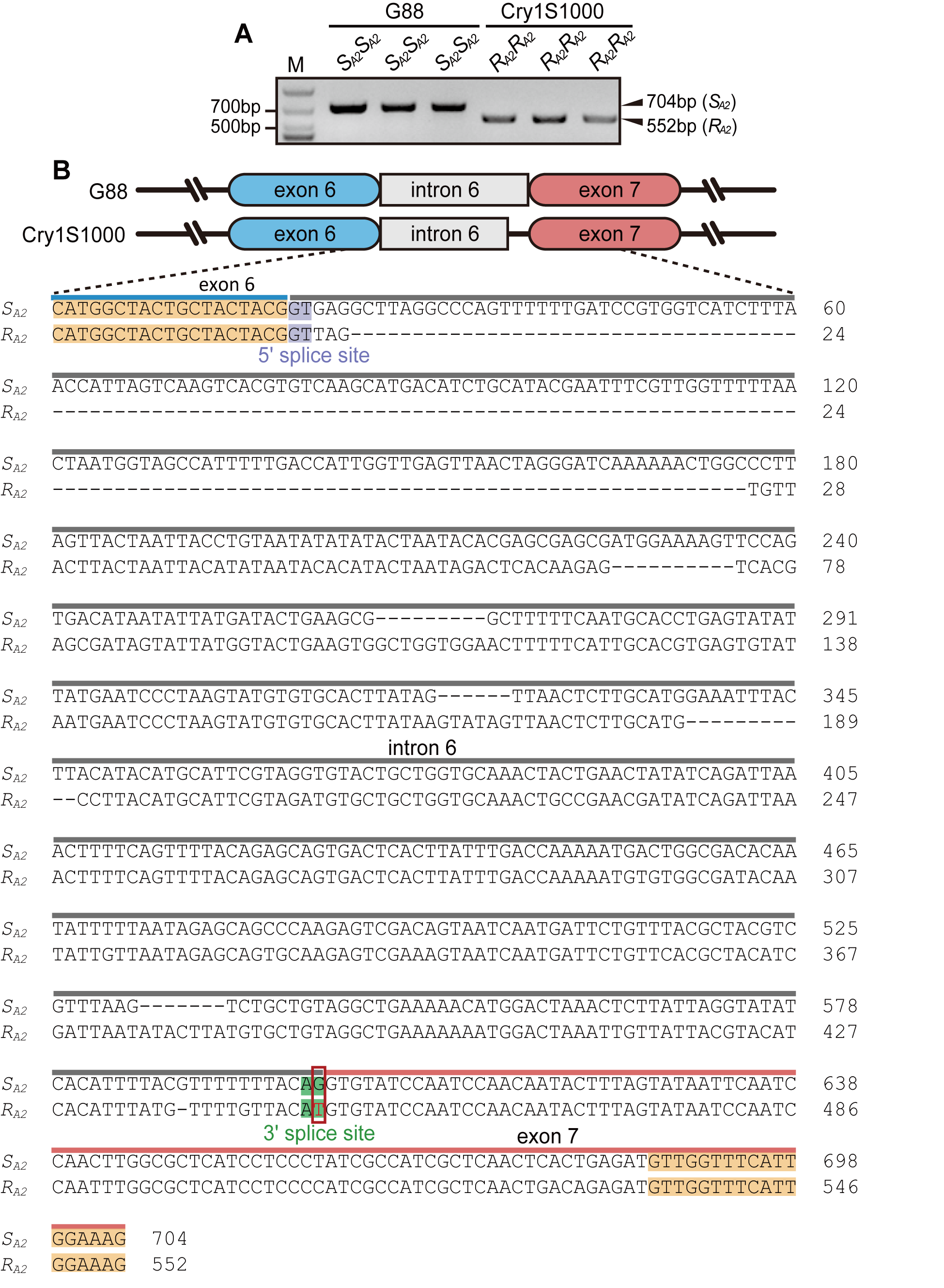

Supplement: S7 Fig — (A) Genotyping of PxABCC2 in G88 and Cry1S1000 individuals using AS-PCR. (B) Alignment of gDNA sequences of the SA2 allele (GenBank accession no. MN660243) and the RA2 allele (GenBank accession no. MN660241). Letter in red indicates the point mutation in 3′ splice site of intron 6. Two sequences highlighted in orange are the primer of 33fPxABCC2, and a sequence matched by the primer of 55PxABCC2. (TIF) [file ppat.1008697.s019.tif]

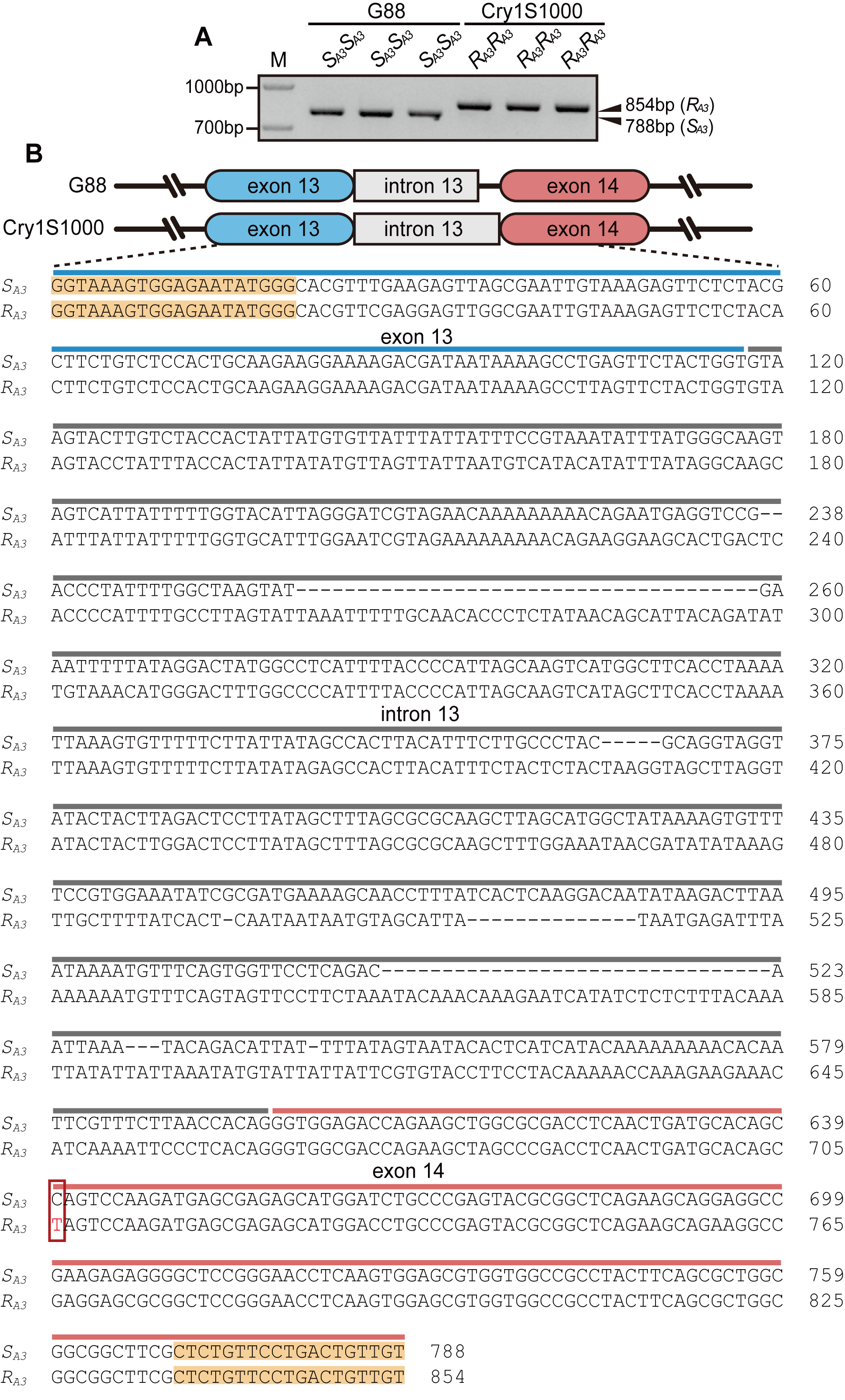

Supplement: S10 Fig — (A) Genotyping of PxABCC3 in G88 and Cry1S1000 individuals using AS-PCR. (B) Alignment of gDNA sequences of the SA3 allele (GenBank accession no. MN660244) and the RA2 allele (GenBank accession no. MN660242). Letter in red indicates the point mutation in exon 14. Two sequences highlighted in orange are the primer of 58PxABCC3, and a sequence matched by the primer of 37rPxABCC3. (TIF) [file ppat.1008697.s022.tif]

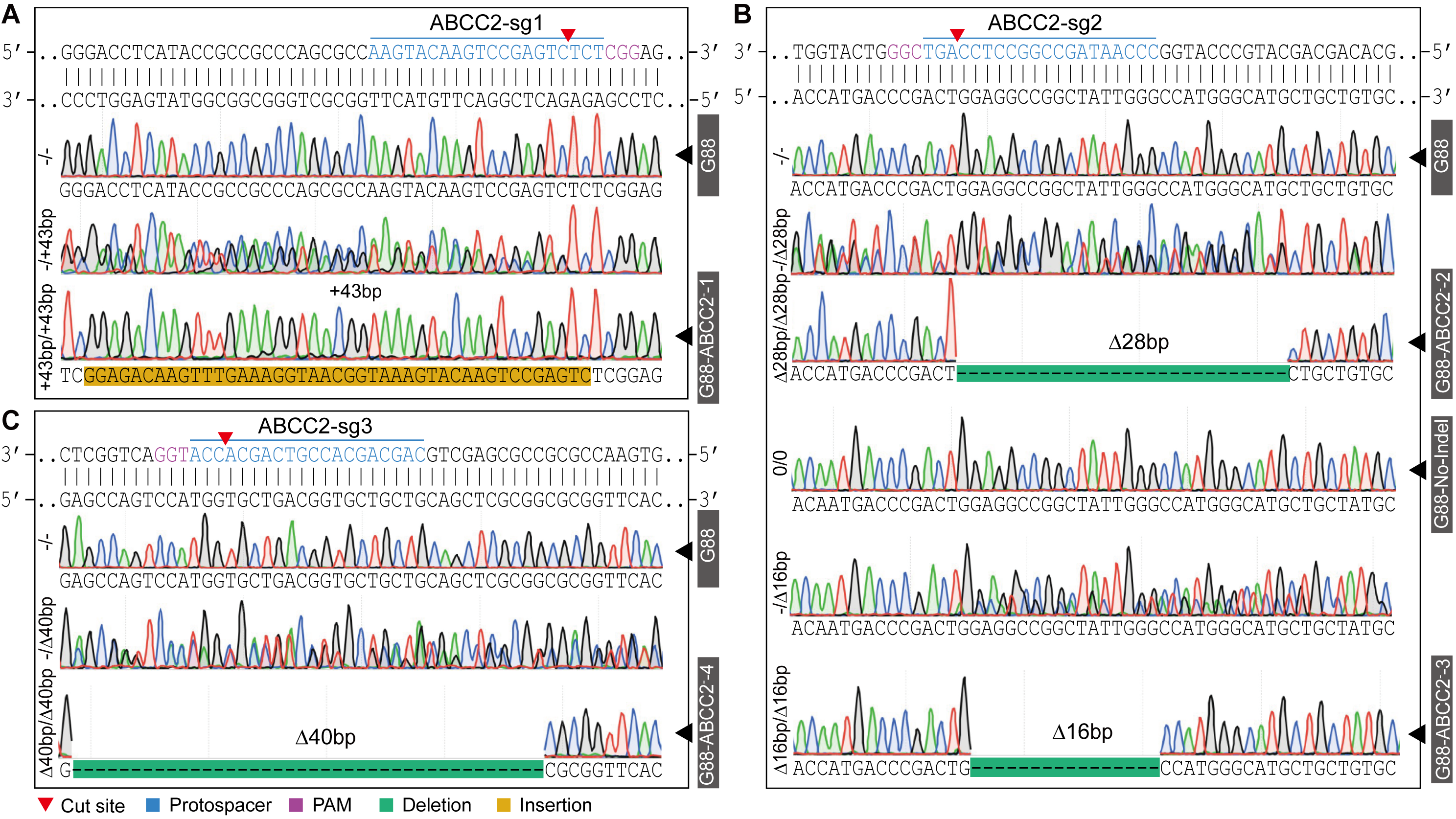

Supplement: S11 Fig — (TIF) [file ppat.1008697.s023.tif]

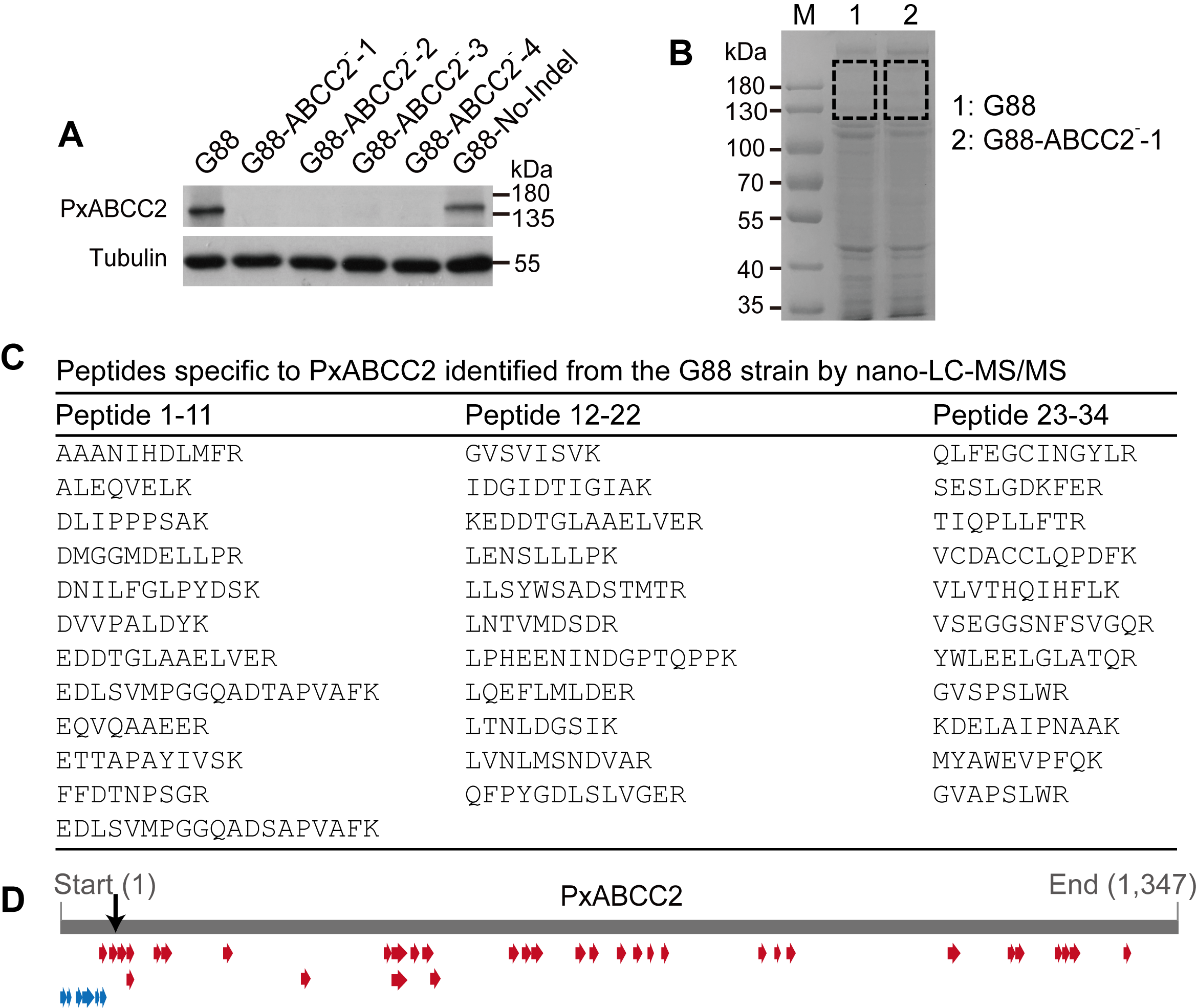

Supplement: S12 Fig — (A) Analysis of PxABCC2 in the wild type G88 strain, four PxABCC2 knockout strains and G88-No-Indel using the western blotting. PxABCC2 antibody recognizes a region from extracellular loop 3 to partial nucleotide-binding domain 1 as indicated in the amino acid sequence of full-length PxABCC2 protein (S2 Fig). (B) SDS-PAGE profile of midgut BBMV protein from G88 and one PxABCC2 knockout strain, G88-ABCC2--1. The dashed rectangles indicate the region of SDS-PAGE gel excised and analyzed by nano-LC-MS/MS. (C) Details of 34 peptides specific to PxABCC2 identified from the G88 strain. None of PxABCC2 peptides were detected from the G88-ABCC2--1 strain. (D) Map of the full-length PxABCC2 protein showing the position of 31 peptides (red arrows) specific to PxABCC2 identified from the G88 strain. Black arrow indicates where the protein of G88-ABCC2--1 is expected to be truncated. The predicted tryptic fragments (no detection from the mutant strain using nano-LC-MS/MS analysis) for expected truncated PxABCC2 from G88-ABCC2--1 are indicated with blue arrows. Numbers indicate the position of amino acid residues. (TIF) [file ppat.1008697.s024.tif]

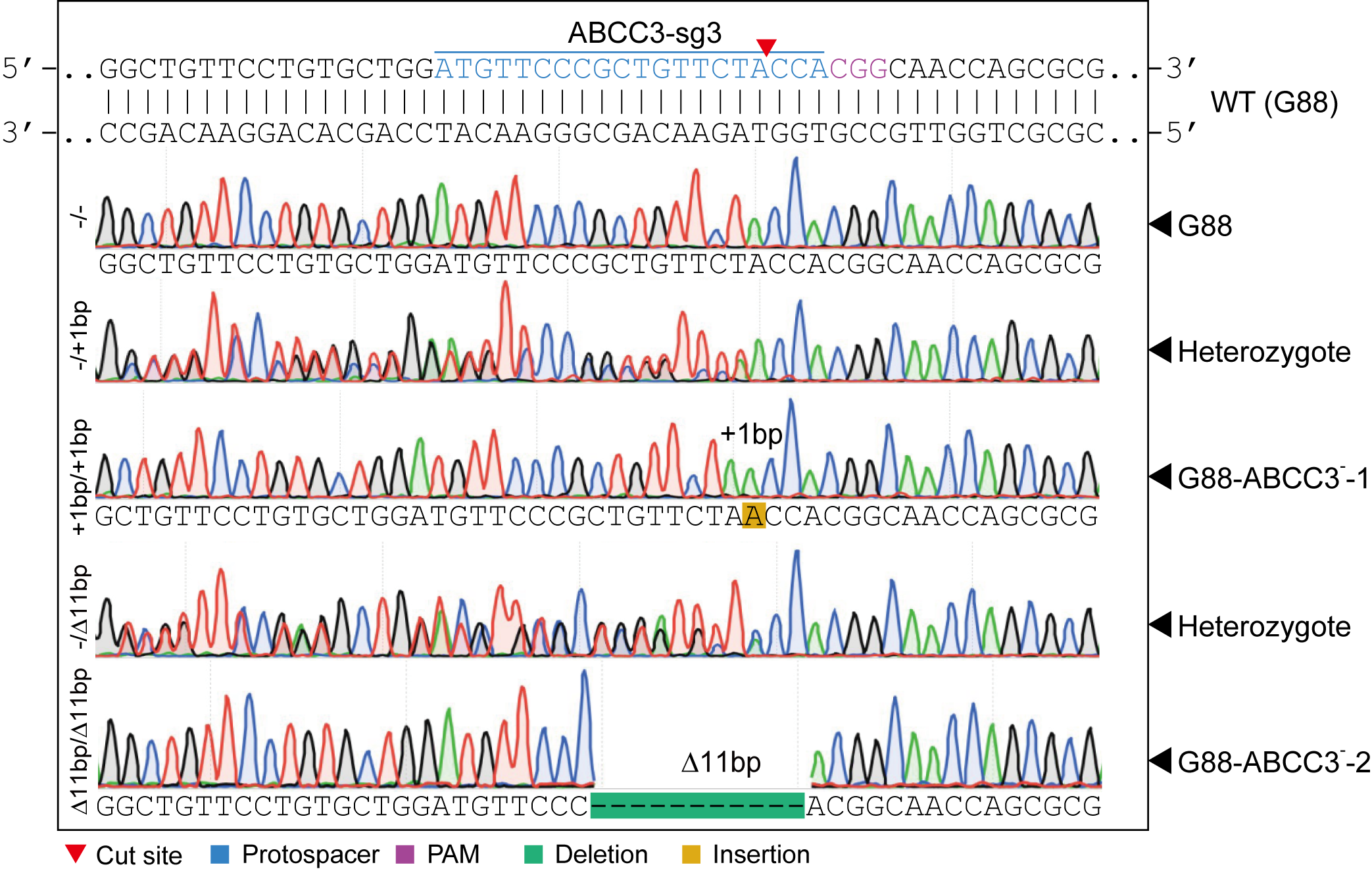

Supplement: S13 Fig — (TIF) [file ppat.1008697.s025.tif]

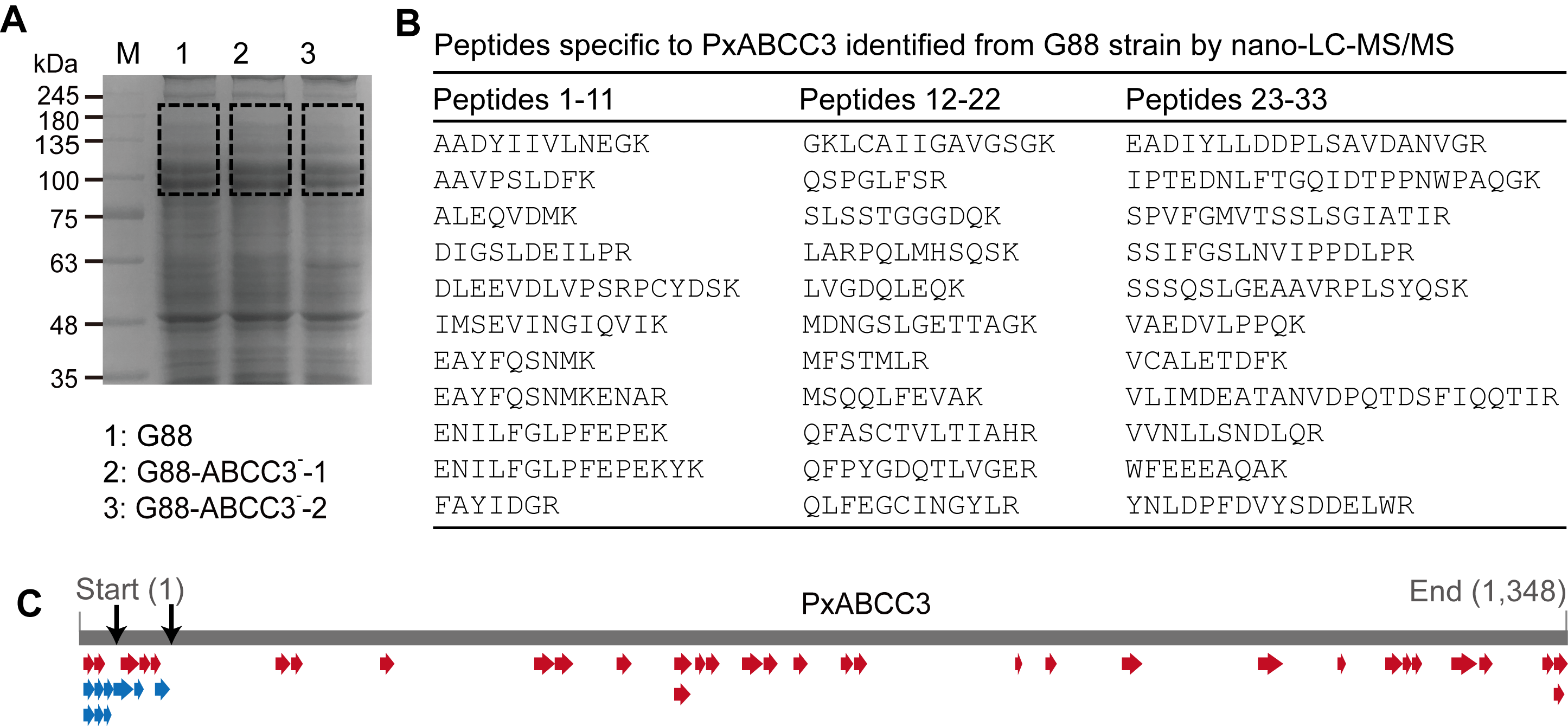

Supplement: S14 Fig — (A) SDS-PAGE profile of BBMV protein from the strain of G88 and two PxABCC3 knockout strains of G88-ABCC3--1 and G88-ABCC3--2. The dashed rectangles indicate the region of SDS-PAGE gel excised and analyzed by nano-LC-MS/MS. (B) Details of the 33 peptides specific to PxABCC3 identified from the G88 strain. None of PxABCC3 peptides were detected from two PxABCC3 knockout strains. (C) Map of the full-length PxABCC3 protein showing the position of 33 peptides (red arrows) specific to PxABCC3 identified from the G88 strain. Black arrows indicate where the proteins of G88-ABCC3--1 and G88-ABCC3--2 are expected to be truncated. The predicted tryptic fragments (no detection from two mutant strains using nano-LC-MS/MS analysis) for expected truncated PxABCC3 proteins from G88-ABCC3--1 and G88-ABCC3--2 are indicated with blue arrows. Numbers indicate the position of amino acid residues. (TIF) [file ppat.1008697.s026.tif]

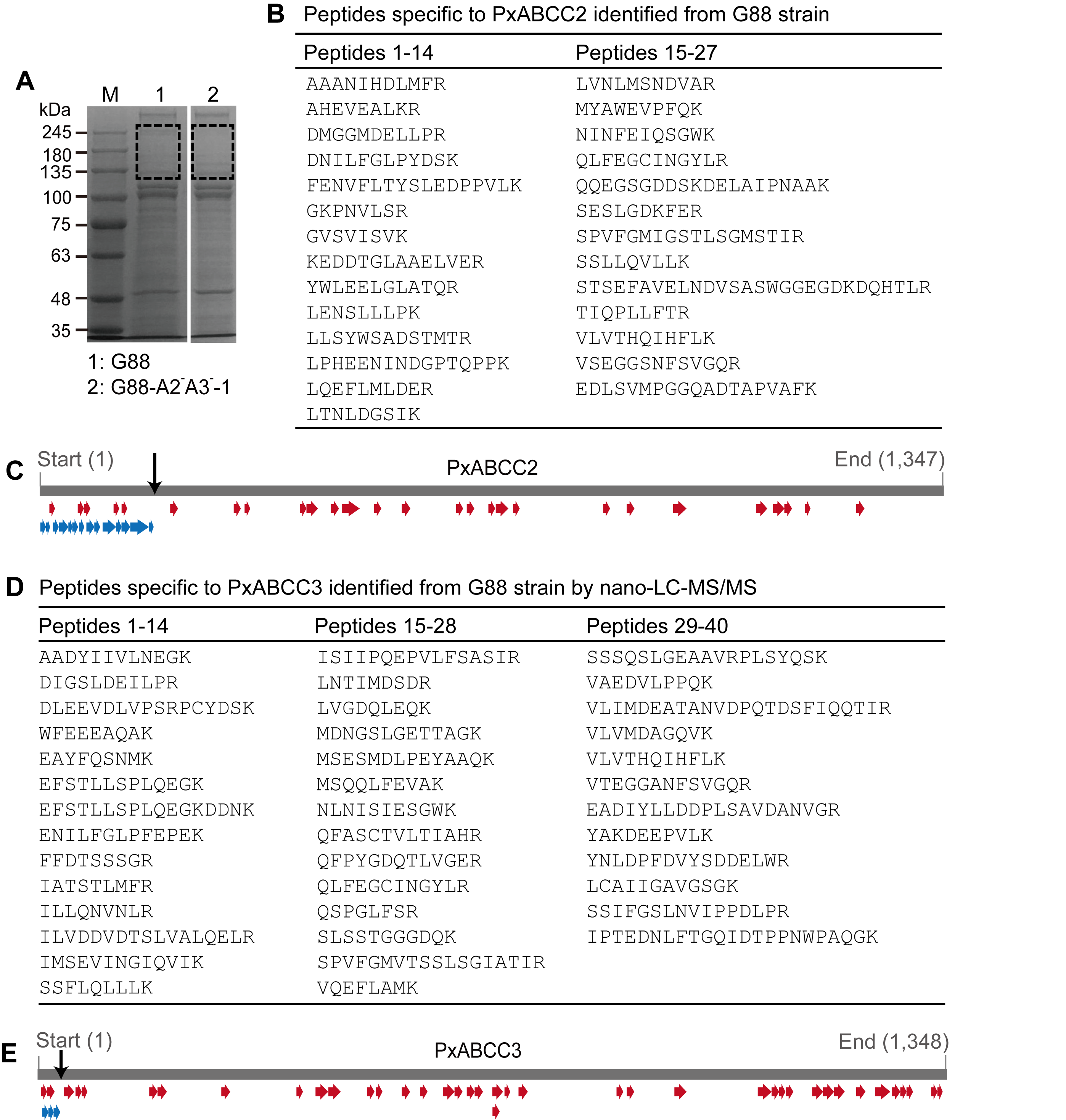

Supplement: S15 Fig — (A) SDS-PAGE profile of BBMV protein from the G88 strain and one PxABCC2/PxABCC3 double mutant strain, G88-A2-A3--1. The dashed rectangles indicate the region of SDS-PAGE gel excised and analyzed by nano-LC-MS/MS. (B) Details of the 27 peptides specific to PxABCC2 identified from the G88 strain. None of PxABCC2 peptides were detected from the G88-A2-A3--1 strain. (C) Map of the full-length PxABCC2 protein showing the position of 28 peptides (red arrows) specific to PxABCC2 identified from the G88 strain. Black arrow indicates where the protein of G88-A2-A3--1 is expected to be truncated. The predicted tryptic fragments (no detection from the mutant strain using nano-LC-MS/MS analysis) for expected truncated PxABCC2 proteins from G88-A2-A3--1 are indicated with blue arrows. Numbers indicate the position of amino acid residues. (D) Details of the 40 peptides specific to PxABCC3 identified from the G88 strain. None of PxABCC3 peptides were detected from the G88-A2-A3--1 strain. (E) Map of the full-length PxABCC3 protein showing the position of 40 peptides (red arrows) specific to PxABCC3 identified from the G88 strain. Black arrow indicates where the protein of G88-A2-A3--1 is expected to be truncated. The predicted tryptic fragments (no detection from the mutant strain using nano-LC-MS/MS analysis) for expected truncated PxABCC3 proteins from G88-A2-A3--1 are indicated with blue arrows. Numbers indicate the position of amino acid residues. (TIF) [file ppat.1008697.s027.tif]

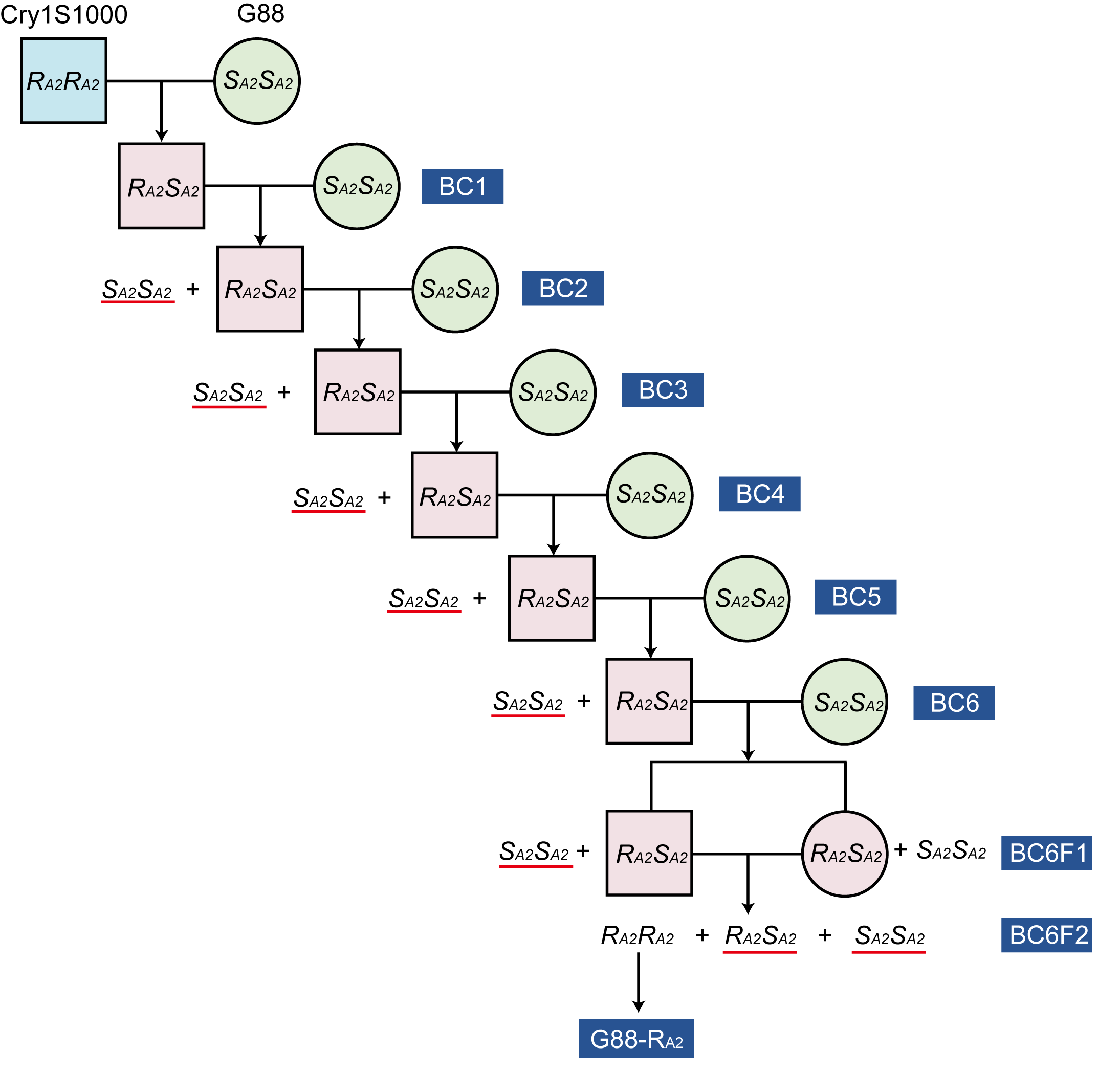

Supplement: S16 Fig — Square and circle represent a male adult and a female adult, respectively. BC1-6: 1–6 generations of backcrossing. BC6F1 and BC6F2 are two additional generations for sibling crosses in single pair to generate a homozygous strain with RA2RA2 (G88-RA2). SA2SA2/RA2SA2 underlined with the red line represent the filtered individuals of each generation after molecular identification using AS-PCR. (TIF) [file ppat.1008697.s028.tif]

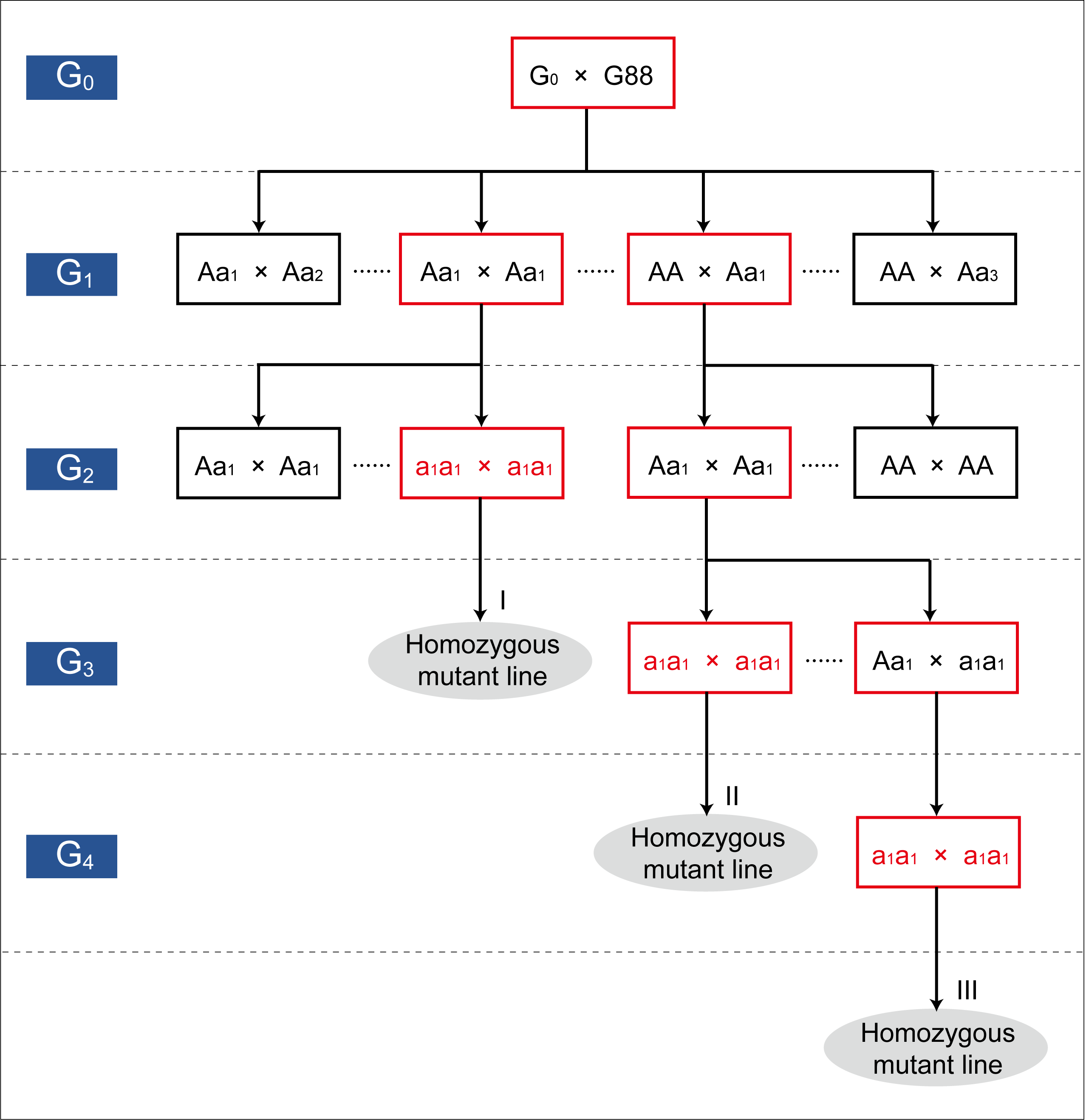

Supplement: S17 Fig — Each rectangle represents a paired female and male. Red rectangles represent the single-pair families kept for the following generations whose parent(s) harbored the desire frameshift mutations as determined by PCR and direct sequencing. “A” shown in the rectangles is the wild type allele; “a1”, “a2” and “a3” are mutant alleles with different type of indel mutations. (TIF) [file ppat.1008697.s029.tif]
