# Supplementary material for: Resistance to Bacillus thuringiensis Cry1Ac toxin requires mutations in two Plutella xylostella ATP-binding cassette transporter paralogs
Source: PLoS Pathog. 2020 Aug 10;16(8):e1008697. doi: 10.1371/journal.ppat.1008697 (PMC7446926; doi:10.1371/journal.ppat.1008697)
Supplement: S8 Fig — Asterisks denote consensus sequences. Codons highlighted in red indicate premature stop codon. (DOC) [file ppat.1008697.s020.doc]

**S8 Fig.**

ABCC3_S ATGGGGGTGAAGGTTGCGGAGGATGTGTTGCCGCCTCAGAAGCAGTCTCCGGGGTTGTTT 60

ABCC3_R ATGGGGGTGAAGGTTGCGGAGGATGTGTTGCCGCCTCAGAAGCAGTCTCCGGGGTTGTTT 60

************************************************************

ABCC3_S TCGAGGCTGTTCCTGTGCTGGATGTTCCCGCTGTTCTACCACGGCAACCAGCGCGACCTG 120

ABCC3_R TCGAGGCTGTTCCTGTGCTGGATGTTCCCGCTGTTCTACCACGGCAACCAGCGCGACCTG 120

************************************************************

ABCC3_S GAGGAGGTGGACCTGGTGCCCTCGCGGCCCTGCTACGATTCCAAGCTGGTGGGCGACCAG 180

ABCC3_R GAGGAGGTGGACCTGGTGCCCTCGCGGCCCTGCTACGACTCCAAGCTGGTGGGCGACCAG 180

************************************** *********************

ABCC3_S CTCGAACAAAAATGGTTCGAAGAAGAAGCTCAAGCCAAGCTGGAGGGTCGGGAGCCGTCG 240

ABCC3_R CTCGAACAAAAATGGTTCGAAGAAGAAGCTCAAGCAAAGCTGGAGGGCCGGGAGCCGTCG 240

*********************************** *********** ************

ABCC3_S TACCCCAAGGTGTTATTTAGGACCTTCTTCTGGTCCTACGTGCCTGGGGGCCTTATGCAG 300

ABCC3_R TACCCCAAGGTGTTATTTAGGACCTTCTTCTGGTCCTACGTGCCTGGGGGCCTTATGCAG 300

************************************************************

ABCC3_S TTTGGATATATCACTTTTCGCACACTATCCCCGCTGCTGTTCGCGGAGCTGCTGAGCTAC 360

ABCC3_R TTTGGATATATCACGTTTCGCACCCTATCCCCGCTACTGTTCGCGGAGCTGCTGAGCTAC 360

************** ******** *********** ************************

ABCC3_S TGGACGGTGGACAGCACCATGACCCGCACGACGGCCACGTACTACACGGTGGGCATGGTG 420

ABCC3_R TGGACGGTGGACAGCACCATGACCCGCACGACGGCCACGTACTACACGGTGGGCATGGTG 420

************************************************************

ABCC3_S GCGTGCAACTGGAGCGCCGCCTACCTCAACCACCAGGGCAACTTCTACTGCCAGCAGTTC 480

ABCC3_R GCGTGCAACTGGAGCGCCGCCTACCTCAACCACCAGGGCAACTTCTACTGCCAGCAGTTC 480

************************************************************

ABCC3_S GGCATGAAGCTGCGCATTGCTACTAGCACGCTCATGTTTAGAAAGATAATGCGCATGGAC 540

ABCC3_R GGCATGAAGCTGCGCATTGCTACCAGCACGCTCATGTTTAGAAAGATAATGCGCATGGAC 540

*********************** ************************************

ABCC3_S AACGGCTCCCTCGGCGAGACCACAGCCGGCAAGGTCGTCAACCTGCTGTCCAACGACCTG 600

ABCC3_R AACGGCTCGCTCGGCGAGACCACAGCCGGCAAGGTAGTCAACCTGCTCTCCAACGACCTG 600

******** ************************** *********** ************

ABCC3_S CAGCGCTTCGACCTGGCGTTCCTGTTCCTGCACTACGTGTGGATCATCCCGCTGCAGCTG 660

ABCC3_R CAGCGCTTCGACCTGGCGTTCCTGTTCCTGCACTACGTGTGGATCATCCCGCTGCAGCTC 660

***********************************************************

ABCC3_S ACGGCCGTGTGCTACCTCGGCTACCGGCAGGCCGGGTTGGCGGCTCTCATCGGCCTCGCC 720

ABCC3_R ACCGCCGTGTGCTACCTCGGCTACCGGCAGGCCGGCGTGGCCGCTCTGATTGGTCTGGCT 720

** ******************************** **** ***** ** ** ** **

ABCC3_S GCGCTGGTGGTCATCGCGCTGCCGATGCAAGGTGGGTTGGGCCGGCTGCTCGGCACGCTG 780

ABCC3_R GCGCTGGTAGTCATCGCGCTGCCTATGCAAGGTGGGCTGGGCCGGCTGCTCGGCACGCTG 780

******** ************** ************ ***********************

ABCC3_S CGCATGAAGACCGCAGAGAAAACCGACGCCCGGATCAAAATTATGAGCGAAGTCATTAAC 840

ABCC3_R CGCATGAAGACCGCAGAGAAAACCGACGCCCGGATCAAAATTATGAGCGAAGTCATTAAC 840

************************************************************

ABCC3_S GGGATCCAGGTGATCAAGATGTACGCGTGGGAGATCCCGTTCGAGAAGGTGGTGGGCGCG 900

ABCC3_R GGAATCCAGGTGATCAAGATGTACGCGTGGGAGATCCCGTTCGAGAAGGTGGTGGGCGCG 900

** *********************************************************

ABCC3_S CGGCGCTGGGAGGAGATGGTGGTGGTGCGCGCGGCCACGCGGATCCGCGCCGTGTTCCTC 960

ABCC3_R CGGCGCTGGGAGGAGATGGTGGTGGTGCGCGCGGCCACGCGGATCCGCGCCGTGTTCCTC 960

************************************************************

ABCC3_S GGCTTCATGGTATTCACGGAGCGGACGGCGCTCTTCCTCACCATCGTCACCTACGTGCTG 1020

ABCC3_R GGCTTCATGGTGTTCACGGAGCGGACCGCGCTCTTCCTCACCATCGTCACCTACGTGCTG 1020

*********** ************** *********************************

ABCC3_S CTCGGCAACACCGTCTCCGCCACCGTCATCTACCCGCTGCAGCAGTTCATGGCGGCGGCG 1080

ABCC3_R CTCGGCAACACCGTCTCCGCCACCGTCATCTACCCGCTGCAGCAGTTCATGGCGGCGGCG 1080

************************************************************

ABCC3_S CAGGTGAACATCACGCTGATCCTGCCCATGGTGCTGTCCTTCACCGCCGAGCTGTTCGTC 1140

ABCC3_R CAGGTGAACATCACGCTGATCCTGCCCATGGTGCTGTCCTTCACCGCCGAGCTGTTCGTC 1140

************************************************************

ABCC3_S TCGCTCCGACGCGTGCAGGAGTTCCTGGCCATGAAGGACCGTTCCGATCTGGTTATAAAG 1200

ABCC3_R TCGCTCCGACGCGTGCAGGAGTTCCTGGCCATGAAGGACCGTTCCGATCTGGTTATAAAG 1200

************************************************************

ABCC3_S AACGTGTCCGGCGGCCAGAAGAAAATGTTCCGCAAGTCGAGCAGCCAGTCCCTGGGCGAG 1260

ABCC3_R AACGTGTCTGGCGGCCAGAAGAAAATGTTCCGCAAGTCGAGCAGCCAGTCCCTGGGCGAG 1260

******** ***************************************************

ABCC3_S GCTGCGGTCCGGCCCTTGTCTTATCAGTCCAAGTCCTCCATATTCGGAAGCCTCAACGTG 1320

ABCC3_R GCTGCGGTCCGGCCCTTGTCTTATCAATCCAAGTCTTCCATATTCGGCAGCCTCAACGTG 1320

************************** ******** *********** ************

ABCC3_S ATCCCCCCTGACTTGCCTCGTCGCCGCAGCATGTCGTACCCGGGCGAACTGGCCGTGGAG 1380

ABCC3_R ATCCCGCCCGACTTGCCTCGGCGCCGCAGCATGTCGTACCCGGGCGAGCTGGCGGTGGAG 1380

***** ** *********** ************************** ***** ******

ABCC3_S GTGCGCGACGTGAGCTGCAGCTGGGTGGGCGACGCCAACGTGCTGGCTCTGAAGAATGTC 1440

ABCC3_R GTGCGTGACGTGAGCTGCAGCTGGGTGGGCGACGCCAACGTGCTGGCGCTGAAGAACGTG 1440

***** ***************************************** ******** **

ABCC3_S TCCGTGCGCCTGGCGCGGGGCAAGCTGTGCGCCATAATTGGCGCTGTTGGGTCTGGAAAG 1500

ABCC3_R TCCGTGCGCCTGGCCCGGGGCAAGCTGTGCGCGATTATTGGCGCTGTCGGCTCGGGAAAG 1500

************** ***************** ** *********** ** ** ******

ABCC3_S TCGTCGTTTTTGCAACTGCTCCTAAAGGAACTGCCCGCGGCGTCTGGCACAGTTTCAATA 1560

ABCC3_R TCGTCGTTTTTGCAACTGCTCCTAAAGGAACTGCCCGCGGCGTCTGGCACAGTTTCAATA 1560

************************************************************

ABCC3_S TTCGGCAAGATCTCGTACGCCTGCCAAGAGGCGTGGCTCTTCCCCAATACCGTCCGAGAG 1620

ABCC3_R TTCGGCAAGATCTCGTACGCCTGCCAAGAGGCGTGGCTCTTCCCCAATACCGTCCGAGAG 1620

************************************************************

ABCC3_S AACATACTCTTTGGCCTGCCATTTGAACCGGAAAAATATAAAAGGGTCTGTCGAGTGTGT 1680

ABCC3_R AACATACTCTTTGGCCTGCCATTTGAACCGGAAAAATATAAAAGGGTGTGTCGAGTATGT 1680

*********************************************** ******** ***

ABCC3_S GCCCTGGAGACGGACTTCAAGCAGTTCCCGTACGGAGACCAGACCCTGGTGGGCGAGCGT 1740

ABCC3_R GCCCTGGAGACGGACTTCAAGCAGTTCCCGTACGGAGACCAGACCCTGGTGGGCGAGCGC 1740

***********************************************************

ABCC3_S GGGGTCTCGCTGTCTGGAGGGCAGCGCGCGCGAATCAACCTCGCCAGATCTGTCTATAGA 1800

ABCC3_R GGGGTCTCACTGTCCGGGGGGCAGCGCGCGCGGATCAACCTCGCCAGATCCGTCTATAGA 1800

******** ***** ** ************** ***************** *********

ABCC3_S GAGGCAGATATCTACCTGCTGGACGACCCGCTGTCGGCGGTGGACGCGAATGTGGGTCGG 1860

ABCC3_R GAGGCTGACATCTACCTGCTGGACGACCCCCTATCGGCGGTGGACGCGAATGTGGGTCGG 1860

***** ** ******************** ** ***************************

ABCC3_S CAGCTGTTCGAGGGCTGCATCAACGGCTACCTGCGCGGCCGCACGCGCGTGCTCGTCACG 1920

ABCC3_R CAGCTGTTCGAGGGCTGCATCAACGGCTACCTGCGCGGCCGCACGCGCGTGCTCGTCACG 1920

************************************************************

ABCC3_S CATCAGATACACTTCCTCAAGGCCGCAGACTACATCATTGTGCTTAATGAGGGTAAAGTG 1980

ABCC3_R CATCAGATACACTTCCTCAAGGCCGCAGACTACATCATTGTGCTTAATGAGGGTAAAGTG 1980

************************************************************

ABCC3_S GAGAATATGGGCACGTTTGAAGAGTTAGCGAATTGTAAAGAGTTCTCTACGCTTCTGTCT 2040

ABCC3_R GAGAATATGGGCACGTTCGAGGAGTTGGCGAATTGTAAAGAGTTCTCTACACTTCTGTCT 2040

***************** ** ***** *********************** *********

ABCC3_S CCACTGCAAGAAGGAAAAGACGATAATAAAAGCCTGAGTTCTACTGGTGGTGGAGACCAG 2100

ABCC3_R CCACTGCAAGAAGGAAAAGACGATAATAAAAGCCTTAGTTCTACTGGTGGTGGCGACCAG 2100

*********************************** ***************** ******

ABCC3_S AAGCTGGCGCGACCTCAACTGATGCACAGCCAGTCCAAGATGAGCGAGAGCATGGATCTG 2160

ABCC3_R AAGCTAGCCCGACCTCAACTGATGCACAGCTAGTCCAAGATGAGCGAGAGCATGGACCTG 2160

***** ** ********************* ************************* ***

ABCC3_S CCCGAGTACGCGGCTCAGAAGCAGGAGGCCGAAGAGAGGGGCTCCGGGAACCTCAAGTGG 2220

ABCC3_R CCCGAGTACGCGGCTCAGAAGCAGAAGGCCGAGGAGCGCGGCTCCGGGAACCTCAAGTGG 2220

************************ ******* *** * *********************

ABCC3_S AGCGTGGTGGCCGCCTACTTCAGCGCTGGCGGCGGCTTCGCTCTGTTCCTGACTGTTGTT 2280

ABCC3_R AGCGTGGTGGCCGCCTACTTCAGCGCTGGCGGCGGCTTCGCTCTGTTCCTGACTGTTGTT 2280

************************************************************

ABCC3_S TGCATCTTCGGCGCGGCTGCCGCGGCTGCTGGTGCTGACTTCTGGGTCAGCTATTGGACA 2340

ABCC3_R TGCATCTTCGGTGCGGCTGCCGCGGCCGCTGGTGCTGACTTCTGGGTCAGCTATTGGACA 2340

*********** ************** *********************************

ABCC3_S AACCAAGTGGCGATTCATGAAGAACAGTTAGCAGGAGCTGAATTAGAGCCAGGCCTGGAC 2400

ABCC3_R AACCAAGTGGCGATTCATGAAGAACAGTTAGCAGGAGCTGAATTAGAGGCAGGCCTGGAC 2400

************************************************ ***********

ABCC3_S GTGCAGATGGGCCGGTTCACGACGCGCACGTACATCATCTTCCACGGCTGCATCGTGGGC 2460

ABCC3_R GTGCAGATGGGCCGGTTCACGACGCGCACGTACATCATCTTCCACGGCTGCATCGTGGGC 2460

************************************************************

ABCC3_S GCGTGCCTGCTGCTGACCAAGCTGAGGGTGTTCCCCTTCGCGCACGTGTGTGTGACGGCC 2520

ABCC3_R GCGTGCCTGCTGCTGACCAAGCTGAGGGTGTTCCCCTTCGCGCACGTGTGTGTGACGGCC 2520

************************************************************

ABCC3_S TCCGCCAACCTGCACAACCGCATGTTCAGCACCATGCTGCGCGGCGTCATGAGGTTCTTC 2580

ABCC3_R TCCGCCAACCTGCACAACCGCATGTTCAGCACCATGCTGCGCGGCGTCATGAGGTTCTTC 2580

************************************************************

ABCC3_S GATACCAGCTCGTCAGGTCGCATCCTCAACCGTTTCACCAAGGACATCGGCTCCCTGGAC 2640

ABCC3_R GATACTAGCTCGTCAGGTCGCATCCTCAACCGCTTCACCAAGGACATCGGTTCCCTGGAC 2640

***** ************************** ***************** *********

ABCC3_S GAGATCCTGCCGCGGACGCTCCTCGACGTGTTCCAGATCTACAGCACCCTGCTGGCGATC 2700

ABCC3_R GAGATCCTGCCGCGGACGCTCCTCGACGTGTTCCAGATCTACAGCACCCTGCTGGCGATC 2700

************************************************************

ABCC3_S CTGGTGCTGAACGCCGTGGCGCTGTACTGGACGCTGGTGCCGTCGGCCGTGCTGCTCGTG 2760

ABCC3_R CTGGTGCTGAACGCCGTGGCACTGTACTGGACGCTGGTGCCCTCGGCCGTGCTGCTCGTG 2760

******************** ******************** ******************

ABCC3_S ATCTTTGGCTTCGCGGTCAATGTTTATATGAAGGCGGCGCAAAGCATTAAGCGGTTGGAG 2820

ABCC3_R ATCTTTGGCTTCGCGGTCAATGTGTATATGAAGGCGGCGCAAAGCATTAAGCGGCTGGAG 2820

*********************** ****************************** *****

ABCC3_S GGAACTACGAAGAGCCCGGTGTTTGGCATGGTGACGTCATCGCTGAGCGGCATCGCCACC 2880

ABCC3_R GGGACTACGAAGAGCCCGGTGTTCGGCATGGTGACGTCTTCACTCAGCGGCATCGCCACC 2880

** ******************** ************** ** ** ***************

ABCC3_S ATCAGGTCGTCGGGTGCCGAGCAGAGACTCATCGACGAGTTCGACAAGCACCAGGATCTG 2940

ABCC3_R ATCCGGTCCTCGGGCGCCGAGCAGCGACTCATCGACGAGTTCGATAAGCACCAGGATCTC 2940

*** **** ***** ********* ******************* **************

ABCC3_S CACACGTACGCATGGAACGGCTACCTGGGTGGGGGCACCACATTCGGGCTCTACCTGGAC 3000

ABCC3_R CACACCTACGCATGGAACGGCTACCTGGGTGGAGGTACCACATTTGGACTCTACTTGGAC 3000

***** ************************** ** ******** ** ****** *****

ABCC3_S ACCATCTGTCTCGTTTACATGACCACCGTCATCTTCGTGTTTCTCTACATTGATTTCGGC 3060

ABCC3_R ACCATCTGTCTGGTGTACATGACCACCGTCATCTTCGTGTTTCTCTACATTGATTTCGGC 3060

*********** ** *********************************************

ABCC3_S GACGCAGTAGCAGTGGGCAGCGTGGGGCTGGCGGTGACGCAGAGCAACCTGCTGACGTTC 3120

ABCC3_R GATGCAGTAGCAGTGGGCAGCGTGGGGCTGGCGGTGACGCAGAGCAACCTGCTGACGTTC 3120

** *********************************************************

ABCC3_S ATCCTGCAGCACGGCGCGCGCATGCTGGTGGAGTTCCTGGCGCAGCTCACCAGCGTGGAG 3180

ABCC3_R ATCCTGCAGCACGGCGCTCGCATGCTGGTGGAGTTCCTGGCGCAGCTCACCAGCGTGGAG 3180

***************** ******************************************

ABCC3_S CGCGTGCTGGACTACACGCGGATACCGACTGAGGACAACCTGTTCACTGGACAAATTGAT 3240

ABCC3_R CGCGTGCTGGACTACACAAGGATACCGACTGAGGACAACCTGTTCACTGGACAAATTGAT 3240

***************** *****************************************

ABCC3_S ACACCTCCAAACTGGCCAGCTCAAGGAAAGATATTGCTTCAAAACGTGAATCTGCGATAT 3300

ABCC3_R ACACCTCCAAACTGGCCAGCTCAAGGGAAGATATTGCTTCAAAACGTGAATCTGCGGTAC 3300

************************** ***************************** **

ABCC3_S GCTAAGGACGAGGAGCCCGTTCTGAAAAACTTGAATATTTCCATTGAAAGCGGATGGAAG 3360

ABCC3_R GCTAAAGACGAGGAGCCCGTGCTGAAAAACTTGAATATTTCAATTGAAAGCGGATGGAAG 3360

***** ************** ******************** ******************

ABCC3_S GTGGGCATCGTGGGGCGCACGGGCGCGGGCAAGTCGTCGCTGATCTCGGCGCTGTTCCGC 3420

ABCC3_R GTGGGCATCGTGGGGCGCACGGGCGCGGGCAAGTCGTCGCTGATCTCGGCGCTGTTCCGC 3420

************************************************************

ABCC3_S TTCGCCTACATCGACGGACGCATCCTCGTGGATGATGTCGACACCTCGCTCGTCGCGCTA 3480

ABCC3_R TTCGCCTACATCGACGGACGCATCCTCGTGGACGATGTTGACACCTCGCTCGTGGCACTG 3480

******************************** ***** ************** ** **

ABCC3_S CAGGAGCTGCGGTCGAAGATCTCCATCATCCCGCAGGAGCCGGTGCTGTTCTCAGCCTCC 3540

ABCC3_R CAGGAGCTGCGGTCGAAGATCTCCATCATCCCGCAGGAGCCGGTGCTGTTCTCCGCCTCC 3540

***************************************************** ******

ABCC3_S ATCCGCTACAACCTCGACCCCTTCGACGTCTACAGCGACGATGAGTTGTGGCGGGCGCTC 3600

ABCC3_R ATCCGCTACAACCTCGACCCCTTCGACGTCTACAGTGATGATGAACTGTGGCGGGCGCTC 3600

*********************************** ** ***** **************

ABCC3_S GAGCAGGTGGACATGAAGGCGGCGGTGCCCTCGCTGGACTTCAAGGTGACGGAGGGCGGC 3660

ABCC3_R GAGCAGGTGGACATGAAGGCGGCGGTGCCCTCGCTGGACTTCAAGGTGACGGAGGGCGGC 3660

************************************************************

ABCC3_S GCCAACTTCTCCGTGGGGCAGCGGCAGCTCGTGTGCCTCGCGCGCGCCGTGCTGCGCTCC 3720

ABCC3_R GCCAACTTCTCCGTGGGGCAGCGGCAGCTGGTGTGCCTGGCGCGCGCCGTGCTGCGCTCC 3720

***************************** ******** *********************

ABCC3_S AACAAGGTGCTCATCATGGACGAGGCCACCGCCAACGTGGACCCGCAAACCGATAGCTTC 3780

ABCC3_R AACAAGGTGCTCATCATGGACGAGGCCACCGCCAACGTGGACCCACAGACCGACAGCTTC 3780

******************************************** ** ***** ******

ABCC3_S ATCCAGCAGACGATCCGACGCCAGTTCGCGTCCTGCACCGTGCTGACCATCGCGCATCGG 3840

ABCC3_R ATCCAGCAGACGATCCGGCGCCAATTCGCCTCCTGCACAGTGCTCACCATCGCTCACCGA 3840

***************** ***** ***** ******** ***** ******** ** **

ABCC3_S CTTAACACCATCATGGACTCCGACCGAGTGCTGGTGATGGACGCAGGCCAGGTGAAGGAG 3900

ABCC3_R CTTAACACGATCATGGACTCCGACCGAGTGCTGGTGATGGACGCGGGTCAGGTTAAGGAG 3900

******** *********************************** ** ***** ******

ABCC3_S TTCGACCACCCCTACCACCTGCTCTCCGACCCCAACAGCCAGCTCACCGCCATGGTCAAG 3960

ABCC3_R TTCGACCACCCTTACCACCTGCTGTCCGACCCGCACAGCCAGCTCACCGCCATGGTCAAG 3960

*********** *********** ******** **************************

ABCC3_S GAGACCAGCGAGAAGATGTCGCAGCAACTCTTCGAGGTCGCGAAGGAAGCGTATTTTCAA 4020

ABCC3_R GAGACCAGCGAGAAGATGTCGCAGCAACTCTTCGAGGTCGCGAAGGAAGCGTATTTTCAA 4020

************************************************************

ABCC3_S AGCAATATGAAAGAGAACGCTAGGTGA 4047

ABCC3_R AGCAATATGAAAGAGAACGCTAGGTGA 4047

***************************
