# Supplementary material for: Resistance to Bacillus thuringiensis Cry1Ac toxin requires mutations in two Plutella xylostella ATP-binding cassette transporter paralogs
Source: PLoS Pathog. 2020 Aug 10;16(8):e1008697. doi: 10.1371/journal.ppat.1008697 (PMC7446926; doi:10.1371/journal.ppat.1008697)
Supplement: S9 Fig — Asterisks denote consensus sequences. Transmembrane domains (TM) (blue), Walker A and B sequences (yellow), and C motif (green) are shown in the sequence of PxABCC3 protein encoded by the susceptible allele. Transmembrane helices, except for TM1 and TM2, were predicted by Phobius. (DOC) [file ppat.1008697.s021.doc]

**S9 Fig.**

ABCC3_S MGVKVAEDVLPPQKQSPGLFSRLFLCWMFPLFYHGNQRDLEEVDLVPSRPCYDSKLVGDQ 60

ABCC3_R MGVKVAEDVLPPQKQSPGLFSRLFLCWMFPLFYHGNQRDLEEVDLVPSRPCYDSKLVGDQ 60

************************************************************

**TM1**

ABCC3_S LEQKWFEEEAQAKLEGREPSYPKVLFRTFFWSYVPGGLMQFGYITFRTLSPLLFAELLSY 120

ABCC3_R LEQKWFEEEAQAKLEGREPSYPKVLFRTFFWSYVPGGLMQFGYITFRTLSPLLFAELLSY 120

************************************************************

**TM2**

ABCC3_S WTVDSTMTRTTATYYTVGMVACNWSAAYLNHQGNFYCQQFGMKLRIATSTLMFRKIMRMD 180

ABCC3_R WTVDSTMTRTTATYYTVGMVACNWSAAYLNHQGNFYCQQFGMKLRIATSTLMFRKIMRMD 180

************************************************************

**TM3**

ABCC3_S NGSLGETTAGKVVNLLSNDLQRFDLAFLFLHYVWIIPLQLTAVCYLGYRQAGLAALIGLA 240

ABCC3_R NGSLGETTAGKVVNLLSNDLQRFDLAFLFLHYVWIIPLQLTAVCYLGYRQAGVAALIGLA 240

****************************************************:*******

**TM4**

ABCC3_S ALVVIALPMQGGLGRLLGTLRMKTAEKTDARIKIMSEVINGIQVIKMYAWEIPFEKVVGA 300

ABCC3_R ALVVIALPMQGGLGRLLGTLRMKTAEKTDARIKIMSEVINGIQVIKMYAWEIPFEKVVGA 300

************************************************************

**TM5**

ABCC3_S RRWEEMVVVRAATRIRAVFLGFMVFTERTALFLTIVTYVLLGNTVSATVIYPLQQFMAAA 360

ABCC3_R RRWEEMVVVRAATRIRAVFLGFMVFTERTALFLTIVTYVLLGNTVSATVIYPLQQFMAAA 360

************************************************************

**TM6**

ABCC3_S QVNITLILPMVLSFTAELFVSLRRVQEFLAMKDRSDLVIKNVSGGQKKMFRKSSSQSLGE 420

ABCC3_R QVNITLILPMVLSFTAELFVSLRRVQEFLAMKDRSDLVIKNVSGGQKKMFRKSSSQSLGE 420

************************************************************

ABCC3_S AAVRPLSYQSKSSIFGSLNVIPPDLPRRRSMSYPGELAVEVRDVSCSWVGDANVLALKNV 480

ABCC3_R AAVRPLSYQSKSSIFGSLNVIPPDLPRRRSMSYPGELAVEVRDVSCSWVGDANVLALKNV 480

************************************************************

**Walker A**

ABCC3_S SVRLARGKLCAIIGAVGSGKSSFLQLLLKELPAASGTVSIFGKISYACQEAWLFPNTVRE 540

ABCC3_R SVRLARGKLCAIIGAVGSGKSSFLQLLLKELPAASGTVSIFGKISYACQEAWLFPNTVRE 540

************************************************************

**C Motif**

ABCC3_S NILFGLPFEPEKYKRVCRVCALETDFKQFPYGDQTLVGERGVSLSGGQRARINLARSVYR 600

ABCC3_R NILFGLPFEPEKYKRVCRVCALETDFKQFPYGDQTLVGERGVSLSGGQRARINLARSVYR 600

************************************************************

**Walker B**

ABCC3_S EADIYLLDDPLSAVDANVGRQLFEGCINGYLRGRTRVLVTHQIHFLKAADYIIVLNEGKV 660

ABCC3_R EADIYLLDDPLSAVDANVGRQLFEGCINGYLRGRTRVLVTHQIHFLKAADYIIVLNEGKV 660

************************************************************

ABCC3_S ENMGTFEELANCKEFSTLLSPLQEGKDDNKSLSSTGGGDQKLARPQLMHSQSKMSESMDL 720

ABCC3_R ENMGTFEELANCKEFSTLLSPLQEGKDDNKSLSSTGGGDQKLARPQLMHS---------- 710

**************************************************

**TM7**

ABCC3_S PEYAAQKQEAEERGSGNLKWSVVAAYFSAGGGFALFLTVVCIFGAAAAAAGADFWVSYWT 780

ABCC3_R ------------------------------------------------------------ 710

**TM8**

ABCC3_S NQVAIHEEQLAGAELEPGLDVQMGRFTTRTYIIFHGCIVGACLLLTKLRVFPFAHVCVTA 840

ABCC3_R ------------------------------------------------------------ 710

**TM9**

ABCC3_S SANLHNRMFSTMLRGVMRFFDTSSSGRILNRFTKDIGSLDEILPRTLLDVFQIYSTLLAI 900

ABCC3_R ------------------------------------------------------------ 710

**TM10**

ABCC3_S LVLNAVALYWTLVPSAVLLVIFGFAVNVYMKAAQSIKRLEGTTKSPVFGMVTSSLSGIAT 960

ABCC3_R ------------------------------------------------------------ 710

**TM11**

ABCC3_S IRSSGAEQRLIDEFDKHQDLHTYAWNGYLGGGTTFGLYLDTICLVYMTTVIFVFLYIDFG 1020

ABCC3_R ------------------------------------------------------------ 710

**TM12**

ABCC3_S DAVAVGSVGLAVTQSNLLTFILQHGARMLVEFLAQLTSVERVLDYTRIPTEDNLFTGQID 1080

ABCC3_R ------------------------------------------------------------ 710

**Walker A**

ABCC3_S TPPNWPAQGKILLQNVNLRYAKDEEPVLKNLNISIESGWKVGIVGRTGAGKSSLISALFR 1140

ABCC3_R ------------------------------------------------------------ 710

ABCC3_S FAYIDGRILVDDVDTSLVALQELRSKISIIPQEPVLFSASIRYNLDPFDVYSDDELWRAL 1200

ABCC3_R ------------------------------------------------------------ 710

**C Motif** **Walker B**

ABCC3_S EQVDMKAAVPSLDFKVTEGGANFSVGQRQLVCLARAVLRSNKVLIMDEATANVDPQTDSF 1260

ABCC3_R ------------------------------------------------------------ 710

ABCC3_S IQQTIRRQFASCTVLTIAHRLNTIMDSDRVLVMDAGQVKEFDHPYHLLSDPNSQLTAMVK 1320

ABCC3_R ------------------------------------------------------------ 710

ABCC3_S ETSEKMSQQLFEVAKEAYFQSNMKENAR 1348

ABCC3_R ---------------------------- 710
